# Barriers to care linkage and educational impact on unnecessary MASLD referrals

**DOI:** 10.3389/fmed.2024.1407389

**Published:** 2024-07-25

**Authors:** Jun-Hyuk Lee, Eileen Laurel Yoon, Ju Hyun Oh, Kyunam Kim, Sang Bong Ahn, Dae Won Jun

**Affiliations:** ^1^Department of Family Medicine, School of Medicine, Nowon Eulji Medical Center, Eulji University, Seoul, Republic of Korea; ^2^Department of Internal Medicine, College of Medicine, Hanyang University, Seoul, Republic of Korea; ^3^Hanyang Institute of Bioscience and Biotechnology, Hanyang University, Seoul, Republic of Korea; ^4^Department of Internal Medicine, College of Medicine, Nowon Eulji Medical Center, Eulji University, Seoul, Republic of Korea; ^5^Department of Family Medicine, College of Medicine, Sanggye Paik Hospital, Inje University, Seoul, Republic of Korea

**Keywords:** metabolic dysfunction-associated steatotic liver disease, physicians, educational program, linkage to care, unmet need

## Abstract

**Background:**

The importance of primary care physicians (PCPs) in managing metabolic dysfunction-associated steatotic liver disease (MASLD) has increased. This study aimed to assess the effectiveness of an online educational program on MASLD among physicians.

**Methods:**

In total, 869 physicians (72 physicians at referral centers and 797 PCPs) participated in this study. They completed an initial survey regarding their clinical practices for patients with MASLD, followed by a second online survey 8 weeks after receiving a series of seven weekly sets of educational materials on MASLD.

**Results:**

In the baseline survey, most PCPs did not routinely evaluate the stage of hepatic fibrosis in MASLD; they typically initiated assessments based on elevated liver enzyme levels. Only a limited number of PCPs used vibration-controlled transient elastography. The main hurdles in managing MASLD were “the absence of a fee for patient education” for PCPs and “short consultation time” for referral-center physicians. In the follow-up survey, the percentage of liver fibrosis assessments using noninvasive tests increased from 7.0 to 11.2%. Additionally, evaluations for cardiovascular disease increased from 3.9 to 8.2%, and the risk of ischemic stroke increased from 13.7 to 16.9%. The percentage of immediate referrals of patients to specialists after an MASLD diagnosis decreased from 15.4 to 12.3%.

**Conclusion:**

The discrepancies in management strategies and viewpoints regarding MASLD between PCPs and referral-center physicians can hinder efforts to mitigate the disease burden. Increasing awareness among PCPs regarding MASLD through a 7-week education program led to a reduction in unnecessary referral rates and an increase in cardiovascular evaluations.

## 1 Introduction

Metabolic dysfunction-associated steatotic liver disease (MASLD) is an important non-communicable disease. MASLD is the most common chronic liver disease worldwide, with an estimated prevalence of 32% (1, 2). In addition, between 1999 and 2022, the age-adjusted mortality rate related to MASLD increased significantly, exhibiting an average annual percentage change of 10.0%. This rate rose from 0.2 to 1.7 per 100,000 persons (3). In the United States, two major causes of death in patients with MASLD are liver-related complications and cardiovascular disease (CVD), accounting for 45.83 and 10.33% of all-cause mortality, respectively (4). The financial burden of MASLD is also on the rise, with its economic toll in the US reaching a significant $103 billion, emphasizing its growing economic impact (5). It is imperative for physicians to conduct medical evaluations to assess liver fibrosis and CVD risk in patients with MASLD and to actively engage in providing concrete education and interventions for more intensive management of MASLD, particularly for those identified as high risk (6).

The introduction of the new concept of MASLD allows for more intuitive communication between patients and primary care physicians (PCPs) regarding the cause of the disease and future treatment directions (7). Specifically, MASLD emphasizes metabolic disorders as the etiology of the disease, conveying a direct message to PCPs and patients that effective treatment requires the management of metabolic parameters. However, limited research has explored whether the MASLD concept helps PCPs reduce unnecessary referral rates and comprehensively manage patients’ care. A survey of 479 physicians revealed that only 31% recognized MASLD as clinically significant, and only 33% referred suspected cases to gastroenterologists, especially general practitioners (8). Additionally, 83% acknowledged the need for more education on MASLD. Another global survey of 488 physicians showed that 64% underestimated MASLD prevalence, 65% reported using evidence-based guidelines, and 72% faced challenges in providing lifestyle advice (9). Similarly, in a study of 250 primary practitioners, 83% understood the importance of managing MASLD, but only 46% screened patients with obesity and diabetes mellitus (DM) (10). This evidence suggests that the medical recommendations provided to patients with MASLD are often inaccurate, potentially contributing to an increased disease burden. Recently, a continuing medical education program on MASLD for 28 European primary clinical practitioners revealed significant changes in clinical practices related to MASLD/metabolic dysfunction-associated steatohepatitis (MASH) (11). However, the limited scope of this study makes it challenging to generalize the results. Large-scale interventional data have not yet been compiled on how raising awareness among PCPs regarding MAFLD impacts their practice behaviors related to this disease.

However, data regarding the obstacles faced by PCPs in the treatment of patients with MASLD and the application status of screening tests for with MASLD at high risk are lacking. Furthermore, there is a scarcity of evidence regarding the increasing awareness of MASLD among PCPs, which can lead to a reduction in unnecessary referrals.

To address these gaps, this study aimed to provide comprehensive educational materials on MASLD to a large number of PCPs and assess any resulting changes in their clinical practices.

## 2 Methods

### 2.1 Study design

This was a prospective longitudinal study. All participants provided informed consent. This study was approved by the Institutional Review Board (IRB) of Hanyang University Hospital (IRB number: 2022-08-01-49). All methods were performed in accordance with the relevant regulations and guidelines. This study was designed to send seven weekly sets of online educational materials regarding MASLD to physicians and then conduct online surveys to assess the changes that occurred in their clinical practice before and after reviewing these materials.

### 2.2 Recruiting and initial survey

**Figure 1:**
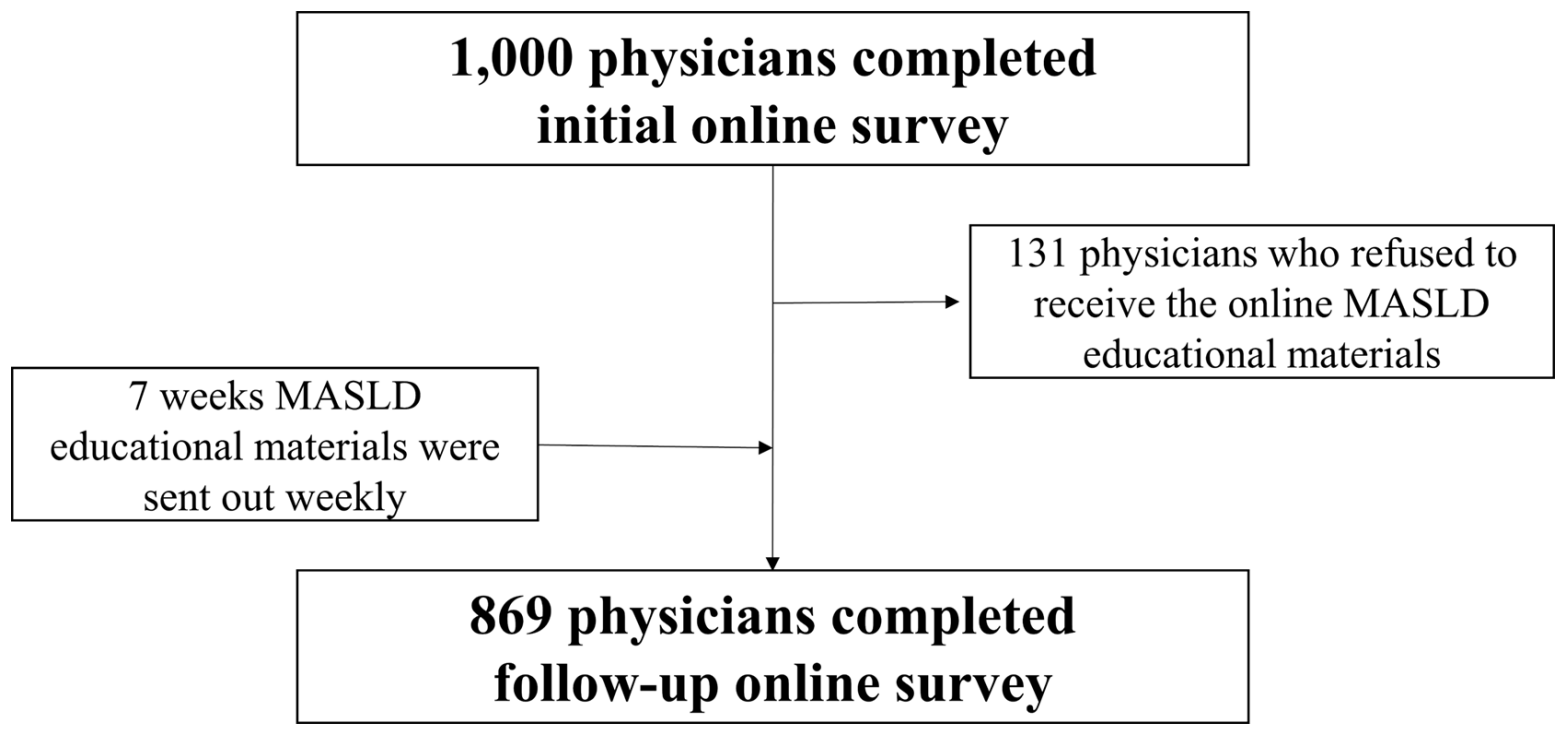
Flowchart of the study population. MASLD, Metabolic dysfunction-associated steatotic liver disease.

This study was conducted using a medical portal website for medical doctors. Doctors who voluntarily accessed the medical portal were asked to participate in the survey and education program. The initial and follow-up surveys were administered online. Figure 1 presents a flowchart of the study. The eligible participants were medical doctors registered on a website exclusively for physicians.^1^ We included 1,000 physicians who responded to the initial survey, which was conducted between October 18, 2022, and October 20, 2022. After excluding 131 respondents who refused to receive educational materials for MASLD, we analyzed a total of 869 participants who responded to the follow-up surveys conducted between December 16, 2022, and January 3, 2023. The standard error of both surveys was ±1.25%, with a 95% confidence interval. Participants were divided into PCPs (*n* = 797) and physicians at referral centers (*n* = 72).

### 2.3 Follow-up and education schedule

Educational materials were sent to the participants weekly between October 27, 2022, and December 8, 2022. They were distributed as image files via a prominent Korean social network service application (KakaoTalk) and tracked by the researchers to confirm participant engagement. Engagement was verified by monitoring whether the files that were sent were opened, providing an initial indication of the participants’ interaction with the educational content.

### 2.4 Contents of questionnaires

Referring to previous survey studies (10, 12), a research group consisting of four physicians (three hepatologists and one primary care physician) developed a 21-item baseline questionnaire and a 16-item follow-up questionnaire (Supplementary material 1) focusing on awareness, current management, barriers, and demand for MASLD. The initial version of the questionnaire was reviewed by seven physicians (four hepatologists, one cardiologist, one endocrinologist, and one neurologist). Cognitive interviews with 10 physicians were conducted to identify whether the respondents’ interpretations matched the intended meaning. This process was repeated three times before the final version of the questionnaire was completed. Content validity was measured by 12 experts, including medical doctors and methodologists, who did not participate in the questionnaire development. The content validity index for each item was rated above 0.80, and the overall content validity index was 0.86. The final version of the questionnaire was read and approved by all developers, and English translation was completed by two native English speakers who were excluded from the study.

### 2.5 Contents of educational materials

The MASLD educational program, comprising seven distinct modules, was methodically disseminated online weekly from the first to seventh week (Supplementary material 2). The modules encompassed the following: (1) an overview of the disease course of MASLD and identification of high-risk groups for screening; (2) methods for assessing liver fibrosis and guidelines for hepatologist referrals; (3) information on the increased risk of CVD in patients with MASLD compared to the general population; (4) the heightened risk of liver cirrhosis and hepatocellular carcinoma in patients with MASLD; (5) weight loss strategies specifically designed for patients with MASLD; (6) dietary recommendations tailored for individuals with MASLD; and (7) exercise regimens suitable for patients with MASLD. Each module was carefully designed to deliver comprehensive and relevant information to the participants.

### 2.6 Statistical analysis

Data representation includes the mean ± standard deviation for continuous variables and the number (percentage, %) for categorical variables. For pre- and post-comparisons in the study group, categorical variables were analyzed using the McNemar test, while continuous variables were evaluated using the paired *t*-test or repeated-measures ANOVA. All statistical analyses were conducted using the R software (version 4.2.1; R Foundation for Statistical Computing, Vienna, Austria). *P* significance was set at *p* < 0.05.

## 3 Results

### 3.1 Baseline characteristics of the study population

Table 1 presents the baseline characteristics of the study population. The mean age was 41.3 ± 8.0 years. The proportion of men was 68.9%. More than half (57.2%) of the respondents lived in metropolitan areas. Over 80% of the respondents were employed by physicians. The most frequent workplace was private clinics (44.6%), followed by hospitals (30.1%), teaching hospitals (13.9%), and others (11.3%).

**Table 1:** Baseline characteristics of the study population.

	Total population (*n* = 869)	PCPs (*n* = 797)	Physicians at referral centers (*n* = 72)	*p*-value
Age, years	41.3 ± 8.0	41.4 ± 8.1	39.5 ± 6.0	0.013
Men, *n* (%)	599 (68.9%)	549 (68.9%)	50 (69.4%)	1.000
Major field, *n* (%)				<0.001
Internal medicine	509 (58.6%)	437 (54.8%)	72 (100.0%)	
Neurology	62 (7.1%)	62 (7.8%)	0 (0.0%)	
Family medicine	192 (22.1%)	192 (24.1%)	0 (0.0%)	
General practice	106 (12.2%)	106 (13.3%)	0 (0.0%)	
Residence type				0.428
Metropolitan area	497 (57.2%)	461 (57.8%)	36 (50.0%)	
Urban cities	191 (22.0%)	172 (21.6%)	19 (26.4%)	
Rural area	181 (20.8%)	164 (20.6%)	17 (23.6%)	
Working type				0.001
Independent practitioner	161 (18.5%)	159 (19.9%)	2 (2.8%)	
Employed physician	696 (80.1%)	626 (78.5%)	70 (97.2%)	
Others	12 (1.4%)	12 (1.5%)	0 (0.0%)	
Working place				<0.001
Private clinic	388 (44.6%)	388 (48.7%)	0 (0.0%)	
Hospital	262 (30.1%)	262 (32.9%)	0 (0.0%)	
Teaching hospital	121 (13.9%)	49 (6.1%)	72 (100.0%)	
Others	98 (11.3%)	98 (12.3%)	0 (0.0%)	

Mean ± standard deviation or number (%). GE, Gastroenterology; PCP, Primary care physician.

### 3.2 Current management status of MASLD among physicians at referral centers compared to PCPs

**Figure 2:**
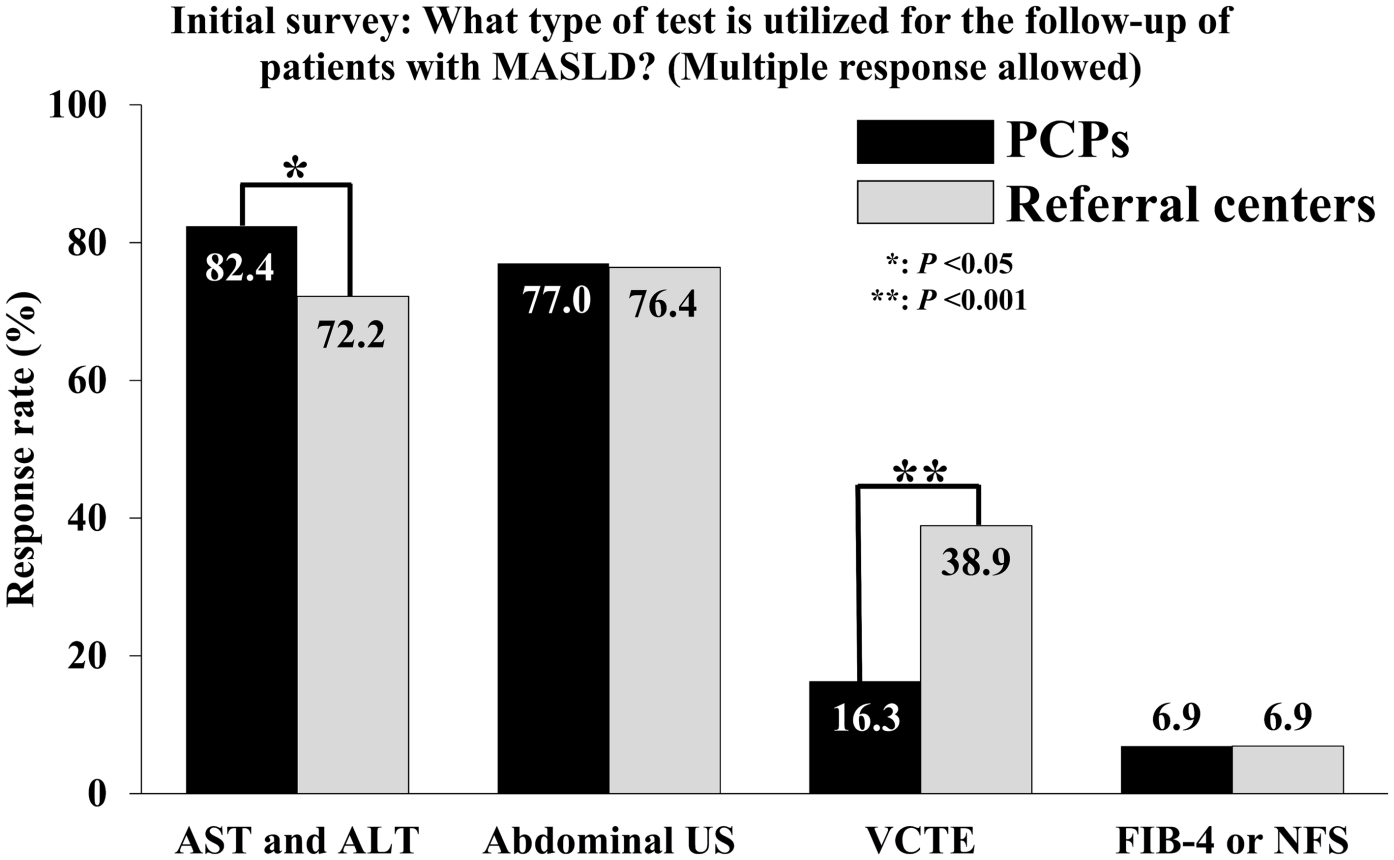
Comparison of follow-up methods for patients with MASLD by PCPs and physicians at referral centers. MASLD, Metabolic dysfunction-associated steatotic liver disease; PCP, Primary care physician; AST, Aspartate aminotransferase; ALT, Alanine aminotransferase; US, Ultrasonography; VCTE, Vibration-controlled transient elastography; FIB-4, Fibrosis-4; NFS, Nonalcoholic fatty liver disease fibrosis score. ^*^p < 0.05; ^**^p < 0.001.

Table 2 outlines the current management of MASLD by comparing specialists at referral centers and PCPs. Physicians at referral centers more frequently assessed the degree of hepatic fibrosis among patients with obesity (54.2 vs. 39.9%, *p* = 0.026) or dyslipidemia (43.1 vs. 29.1%, *p* = 0.020) compared to PCPs. Only 32.6% of the patients with DM and 9.2% of the patients with CVD were evaluated for hepatic fibrosis by PCPs. A similar situation was observed in referral centers, where specialists performed hepatic fibrosis evaluations in only 37.5% of patients with DM and 11.1% of patients with CVD. Figure 2 shows the frequency of utilizing the type of test to follow up on patients with MASLD. Only 6.9% of physicians at referral centers and PCPs used noninvasive tests, such as the Fibrosis-4 Index or the nonalcoholic fatty liver disease fibrosis score (NFS), to evaluate hepatic fibrosis, with no significant difference observed between the two groups. A significant difference was observed in the frequency of liver function tests [aspartate aminotransferase (AST) and alanine aminotransferase (ALT)] and vibration-controlled transient elastography (VCTE) use between PCPs and physicians at referral centers. PCPs used liver function tests as follow-up tests more frequently than physicians at referral centers (82.4 vs. 72.2%, *p* < 0.05). However, the use of VCTE as a follow-up test was lower among PCPs than among physicians at referral centers (16.3 vs. 38.9%, *p* < 0.001).

**Table 2:** Initial survey: current management of MASLD among PCPs compared to physicians at referral centers.

	Total (*n* = 869)	PCPs (*n* = 797)	Physicians at referral centers (*n* = 72)	*p*-value
**Hepatic fibrosis evaluation in patients**				
Never considered	160 (18.4%)	151 (18.9%)	9 (12.5%)	0.233
Old age	167 (19.2%)	150 (18.8%)	17 (23.6%)	0.405
Abnormal AST or ALT	609 (70.1%)	552 (69.3%)	57 (79.2%)	0.104
Obesity	357 (41.1%)	318 (39.9%)	39 (54.2%)	0.026
Diabetes mellitus	287 (33.0%)	260 (32.6%)	27 (37.5%)	0.476
Dyslipidemia	263 (30.3%)	232 (29.1%)	31 (43.1%)	0.020
Metabolic syndrome	312 (35.9%)	279 (35.0%)	33 (45.8%)	0.088
CVD event	81 (9.3%)	73 (9.2%)	8 (11.1%)	0.738
Ischemic stroke	73 (8.4%)	67 (8.4%)	6 (8.3%)	1.000
**Patient education for**				
Liver disease progression	554 (63.8%)	507 (63.6%)	47 (65.3%)	0.878
CVD development	174 (20.0%)	161 (20.2%)	13 (18.1%)	0.778
Ischemic stroke development	119 (13.7%)	109 (13.7%)	10 (13.9%)	1.000
Extra-hepatic cancer risk	98 (11.3%)	93 (11.7%)	5 (6.9%)	0.308

PCP, Primary care physician; VCTE, Vibration-controlled transient elastography; FIB-4, Fibrosis index-4; NFS, Non-alcoholic fatty liver disease fibrosis score; AST, Aspartate aminotransferase; ALT, Alanine aminotransferase; and CVD, Cardiovascular disease.

### 3.3 Clinical barriers in MASLD patient management

Responses to the barriers to managing patients with MASLD are shown in Table 3. A total of 76.4% of physicians at referral centers considered the “short consultation time” a primary challenge, followed by “patients’ low compliance” (76.4%), “lack of appropriate medication” (72.3%), “lack of adequate educational materials” (70.8%), “the absence of a fee for this service” (68.0%), and “not my primary area of practice” (38.9%). In contrast, 72.3% of PCPs cited the “the absence of a fee for this service” as a major barrier, followed by “short consultation time” (67.2%), “patients’ low compliance” (67.1%), “lack of adequate educational materials” (65.1%), “lack of appropriate medication” (61.7%), and “not my primary area of practice” (38.9%).

**Table 3:** Initial survey: barriers to the management of MASLD among specialists at referral centers compared to PCPs.

	PCPs (*n* = 797)					Physicians at referral centers (*n* = 72)				
Five-point Likert scale	Strongly agree	Agree	Unsure	Disagree	Strongly disagree	Strongly agree	Agree	Unsure	Disagree	Strongly disagree
**Management barrier**										
Short consultation time	193 (24.2%)	343 (43.0%)	215 (27.0%)	41 (5.1%)	5 (0.6%)	19 (26.4%)	36 (50.0%)	15 (20.8%)	1 (1.4%)	1 (1.4%)
Lack of adequate educational materials	121 (15.2%)	398 (49.9%)	235 (29.5%)	41 (5.1%)	2 (0.3%)	15 (25.0%)	33 (45.8%)	20 (27.8%)	1 (1.4%)	0 (0.0%)
Not my primary area of practice	90 (11.3%)	220 (27.6%)	292 (36.6%)	156 (19.6%)	39 (4.9%)	6 (8.3%)	22 (30.6%)	27 (37.5%)	13 (18.1%)	4 (5.6%)
Patients’ low compliance	173 (21.7%)	362 (45.4%)	224 (28.1%)	35 (4.4%)	3 (0.4%)	15 (20.8%)	40 (55.6%)	16 (22.2%)	1 (1.4%)	0 (0.0%)
Lack of appropriate medication	127 (15.9%)	365 (45.8%)	241 (30.2%)	58 (7.3%)	6 (0.8%)	12 (16.7%)	40 (55.6%)	19 (26.4%)	1 (1.4%)	0 (0.0%)
Absence of a fee for this service	226 (28.4%)	350 (43.9%)	199 (25.0%)	20 (2.5%)	2 (0.3%)	15 (20.8%)	34 (47.2%)	23 (31.9%)	0 (0.0%)	0 (0.0%)

PCP, Primary care physician.

### 3.4 Changes in clinical practice patterns among PCPs after education

**Figure 3:**
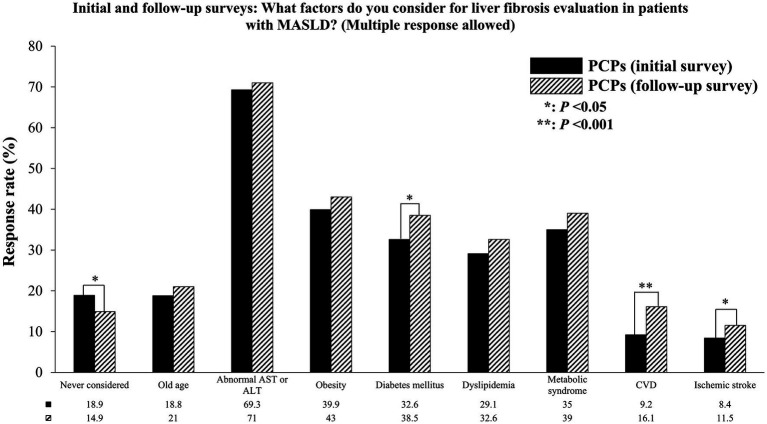
Changes in the percentage of PCPs considering hepatic fibrosis evaluation in patients with MASLD who have comorbidities. PCP, Primary care physician; MASLD, Metabolic dysfunction-associated steatotic liver disease; AST, Aspartate aminotransferase; ALT, Alanine aminotransferase; CVD, Cardiovascular disease. ^*^p < 0.05; ^**^p < 0.001.

Table 4 shows the changes in the management of patients incidentally identified as having MASLD among PCPs before and after the physicians received educational materials related to MASLD. In cases in which PCPs incidentally discovered MASLD in patients, the educational program led to an increase in the percentage of PCPs ordering additional tests to assess hepatic fibrosis from 7.0 to 11.2% (*p* < 0.001). Similarly, the percentage of PCPs performing additional tests to determine the presence of concurrent CVD increased from 3.9 to 8.2% (*p* < 0.001). Figure 3 shows the changes in the percentage of PCPs based on hepatic fibrosis evaluation in patients with MASLD and comorbidities. There has been an increase in the percentage of PCPs who consider evaluating hepatic fibrosis when patients with MASLD also present conditions of DM (from 32.6 to 38.5%), CVD (from 9.2 to 16.1%), or ischemic stroke (from 8.4 to 11.5%).

**Table 4:** Changes in the management of incidentally diagnosed patients with MASLD among PCPs before and after receiving educational materials on MASLD.

(Please respond if you are a PCP) How do you treat a patient who is incidentally found to have MASLD on ultrasound? (Multiple responses allowed)	Initial survey	Follow-up survey	*p*-value^*^
Briefly mention that the patient has MASLD and do not proceed with any additional measures.	131 (16.4%)	153 (19.2%)	0.111
Refer the patient to gastroenterology or a tertiary hospital.	246 (30.9%)	248 (31.1%)	0.943
Take no additional measure if the patient has normal AST and ALT levels.	168 (21.1%)	156 (19.6%)	0.432
Mention that the patient has a fatty liver and recommend lifestyle modification.	460 (57.7%)	459 (57.6%)	1.000
Order additional tests (blood glucose test, lipid panel, etc.) for metabolic syndrome (diabetes, dyslipidemia, etc.).	368 (46.2%)	387 (48.6%)	0.252
Order additional tests regarding liver fibrosis.	56 (7.0%)	89 (11.2%)	<0.001
Order additional tests to determine whether the patient also has CVD.	31 (3.9%)	65 (8.2%)	<0.001

^*^The *p* value was derived from the McNemar test. MASLD, Metabolic dysfunction-associated steatotic liver disease; PCP, Primary care physician; AST, Aspartate aminotransferase; ALT, Alanine aminotransferase; and CVD, Cardiovascular disease.

### 3.5 Changing of awareness and referral rate among PCPs after education

The percentage of PCPs providing education to patients with MASLD regarding the increased risk of disease progression to cirrhosis or liver cancer increased from 63.6% before education to 69.4% after education (*p* = 0.005). Moreover, the proportion of PCPs educating patients regarding the elevated risk of ischemic stroke increased from 13.7 to 16.9% (*p* = 0.039; Table 5). After education, the percentage of PCPs who immediately referred patients to a specialist after an MASLD diagnosis decreased from 15.4 to 12.3% (*p* = 0.042). In contrast, the proportion of patients with MASLD who were referred for suspected advanced hepatic fibrosis (54.7 vs. 60.1%, *p* = 0.018) and of patients at a high risk of CVD or ischemic stroke (12.4 vs. 16.8%, *p* < 0.001) significantly increased (Table 6).

**Table 5:** Changes in the education of patients with MASLD among PCPs before and after receiving educational materials on MASLD.

(Please respond if you are a PCP) What kind of education do you provide patients who have MASLD? (Multiple responses allowed)	Initial survey	Follow-up survey	*p*-value^*^
Provide no further explanation.	54 (6.8%)	62 (7.8%)	0.456
Explain the increased risk of disease progression into cirrhosis or liver cancer.	507 (63.6%)	553 (69.4%)	0.005
Explain the increased risk of metabolic diseases (diabetes, dyslipidemia, etc.).	612 (76.8%)	603 (75.7%)	0.575
Explain the increased risk of CVD.	161 (20.2%)	184 (23.1%)	0.113
Explain the increased risk of ischemic stroke.	109 (13.7%)	135 (16.9%)	0.039
Explain the increased risk of extrahepatic cancer.	93 (11.7%)	106 (13.3%)	0.287

^*^The *p* value was derived from the McNemar test. MASLD, Metabolic dysfunction-associated steatotic liver disease; PCP, Primary care physician; and CVD, Cardiovascular disease.

**Table 6:** Changes in immediate referral cases of patients with MASLD among PCPs before and after receiving educational materials for MASLD.

(Please respond if you are a PCP) In which situations do you refer your patients with nonalcoholic fatty liver disease to gastroenterology or a tertiary hospital? (Multiple responses allowed)	Initial survey	Follow-up survey	*p*-value^*^
Immediately refer to GI once hepatic steatosis is confirmed.	123 (15.4%)	98 (12.3%)	0.042
If AST or ALT level is abnormal.	288 (36.1%)	277 (34.8%)	0.536
If the patient’s hepatic steatosis is severe.	330 (41.4%)	314 (39.4%)	0.373
If there is concern regarding the progression of liver fibrosis or cirrhosis (high severity of liver fibrosis, low platelet count, etc.).	436 (54.7%)	479 (60.1%)	0.018
If the patient has comorbid metabolic disorders (diabetes, dyslipidemia, etc.).	120 (15.1%)	110 (13.8%)	0.477
If the patient has comorbid CVD or ischemic stroke.	124 (15.6%)	194 (24.3%)	<0.001
When considering a variety of treatment options for nonalcoholic fatty liver disease.	89 (11.2%)	85 (10.7%)	0.794
When requested by patients or their families.	99 (12.4%)	134 (16.8%)	0.006

^*^The *p* value was derived from the McNemar test. PCP, Primary care physician; AST, Aspartate aminotransferase; ALT, Alanine aminotransferase; and CVD, Cardiovascular disease.

## 4 Discussion

Despite technological advancements, the importance of direct face-to-face interactions between physicians and patients in providing education and medical services remains paramount. This study, which involved a large cohort of 869 physicians, provided educational materials over approximately 2 months and showed positive changes in the management of MASLD. Several important features regarding the impact of educational materials on PCPs’ management patterns in relation to MASLD should be highlighted. There was an increase in the frequency of fibrosis tests among PCPs in patients with MASLD and a decrease in the rate of immediate referrals to specialists. Additionally, the proportion of physicians educating patients regarding the risks of CVD and ischemic stroke increased, which can be considered a significant motivator for patients to understand the necessity of weight reduction in managing MASLD. Moreover, the rate at which PCPs assessed hepatic fibrosis in patients with DM, CVD, or ischemic stroke significantly increased. These findings highlight the importance of identifying and managing high-risk patients more effectively. These trends demonstrate the potential of online educational programs to change medical practices in a cost-effective manner, ultimately reducing long-term medical expenses.

In primary care, it is essential to identify patients with MASLD and understand the indications for referral to a hepatologist (10). In patients with MASLD, 52% exhibit MASH, and 15% have significant intrahepatic fibrosis (13). Additionally, the risk of developing liver cancer increases with the stage of hepatic fibrosis; individuals with stage 3 fibrosis have a liver cancer incidence rate of 340 per 100,000, which is 8.5 times higher than the rate of 40 per 100,000 observed in individuals with stages 0–2 (14). Therefore, the presence of advanced hepatic fibrosis is an important criterion for referral. In the initial survey, 82.4% of PCPs reported using AST and ALT levels for patient monitoring. However, using only serum AST, ALT, or the AST/ALT ratio as markers for severe MASLD demonstrates low sensitivity for hepatic fibrosis, potentially leading to the misclassification of high-risk patients as mild cases (15, 16). Additionally, a previous study involving 145 patients with diabetes found evidence of liver cirrhosis in 6.0% of patients using magnetic resonance imaging, and 12.8% were identified as having advanced hepatic fibrosis when evaluated using NFS (17). This suggests that the prevalence of undiagnosed liver cirrhosis could be high even in the primary care field, underscoring the necessity of assessing hepatic fibrosis in patients with MASLD. Given the challenges associated with using equipment such as VCTE in primary care clinics, assessment of hepatic fibrosis and its severity via noninvasive tests may be necessary. However, simply providing educational materials was insufficient to encourage physicians who were previously unfamiliar with this information to actively implement serological testing. Nevertheless, for physicians who were already informed regarding these tests, receiving reminders resulted in a 7.4% increase in actual utilization (data not shown). This suggests that continuous and repeated exposure to knowledge is important for eliciting behavioral changes in actual medical practice. However, from the perspective of the various barriers to MASLD management, education alone may not sufficiently address the unmet needs in different specialties and hospital settings.

We performed a sensitivity analysis to assess the pre- and post-education effects on participants who agreed with each barrier item; participants were divided into PCPs and physicians at referral centers (Supplementary Tables 1–6). Overall, education significantly increased the assessment of liver fibrosis and CVD risk among PCPs. Physicians at referral centers also showed a trend toward increased liver fibrosis test assessment. Surprisingly, among PCPs who identified “lack of adequate education materials” as a barrier, the rate of omitting additional explanations for MASLD patients increased from 5.8 to 9.2% post-education. Conversely, the proportion of physicians at referral centers who did not take additional measures for MASLD patients decreased from 23.5 to 7.8% (Supplementary Table 2). Additionally, PCPs who agreed with “not my primary area of practice” as a barrier showed better educational outcomes in management and education practice areas (Supplementary Table 3). Although the sample size for physicians at referral centers was small, making drawing definitive conclusions challenging, those who identified “short consultation time,” “lack of adequate educational materials,” and “patients’ low compliance” as barriers tended to show positive educational effects (Supplementary Tables 1, 2, 4). This suggests that different types of barriers require tailored approaches. However, the significant gap between clinical guidelines and actual clinical practice is of considerable concern.

Although healthcare providers concur with the need for a joint approach to working across disciplines to achieve long-term effective MASLD management (18), the quietly different unmet needs for the management of patients with MASLD between specialists in referral centers and PCPs are should be discussed. Specialists in referral centers face high clinical workloads and treat a large number of patients, making it difficult for them to allocate time for patient education. In particular, managing multifactorial risk factors in referral centers necessitates a multidisciplinary system that includes not only medical education but also paramedical advice such as weight loss, diet control, and exercise regulation, utilizing experts from multiple domains, such as nutritionists and physical therapists.

Regarding PCPs, there is lack of motivation to educate patients with MASLD in primary clinics due to Korea’s specific health insurance system. South Korea operates a national mandatory health insurance system in which healthcare providers submit claims to government agencies based on the medical services provided to patients. However, there are significant limitations on the financial reimbursement that physicians can claim for patient education. Increasing reimbursement support for medical education as well as for the management of liver fibrosis and MASLD could help reduce the barriers that specialists and PCPs face in managing MASLD.

In Asian primary care clinics, using the Fibrosis-4 Index for advanced hepatic fibrosis demonstrated a commendable diagnostic performance with an area under the receiver operating characteristic curve of 0.818, showcasing its utility in primary practice (19). Additionally, the cost-effectiveness analysis indicated that screening the at-risk population led to an incremental cost of $298 and an increase of 0.0199 quality-adjusted life-years (QALY) per patient compared to not screening [incremental cost-effectiveness ratio (ICER) $14,949/QALY] (20). This screening approach was deemed cost effective according to Korea’s implicit ICER threshold of $25,000/QALY. When the impact of intensive lifestyle intervention on CVD and extrahepatic malignancies were factored in, the ICER dropped to $12,749/QALY. On the other hand, when the model focused solely on liver disease effects, the ICER rose to $16,305. Despite these variations, the screening strategy remained cost-effective when assessed against the ICER thresholds of Japan and the United States.

Based on this evidence, if reimbursement for performing noninvasive tests for liver fibrosis management, at a minimum, is introduced in primary care clinics, it could be more cost effective compared to the economic burden of managing liver fibrosis complications. To date, there has been no evaluation of the cost effectiveness of education for MASLD management, and the effect size is crucial. The current study’s results indicate that the standalone effect of merely distributing educational materials was relatively modest across all groups. Future research should explore the effects of various educational methods on linkage to care rates. These findings can serve as a basis for future economic evaluations of educational interventions. Conducting a cost-effectiveness analysis requires confirming the effects of education across different groups, which we propose pursuing in subsequent research.

Individuals who have MASLD have a 66% higher risk of CVD and a 41% higher risk of ischemic stroke than those who do not have MASLD (21). As the severity of MASLD escalates, these risks further intensify; severe fatty liver disease is associated with a 2.6-fold increase in the risk of developing CVD and a 3.3-fold increase in the risk of fatal CVD (22). Although assessing CVD risk in patients with MASLD is critical, the initial survey revealed that only 3.9% of PCPs prescribed additional tests to determine whether their patients had concurrent CVD, which is a strikingly low percentage. After receiving educational materials, this rate increased dramatically to 8.2%, and the proportion of physicians educating patients about the risk of MASLD leading to ischemic stroke increased from 13.7 to 16.9%. However, the survey did not inquire about the specific methods used by physicians to assess and educate patients on CVD, suggesting that actual practice might display a mosaic pattern.

In a survey of 629 American physicians, there were variations in the evaluation and management of patients with MASLD or MASH depending on the subspecialty, and there were major differences in MASLD knowledge and management between PCPs and other subspecialists, including hepatologists, gastroenterologists, and endocrinologists (23). In this study, there was no significant change in the specialists’ MASLD knowledge before and after receiving educational materials. As the educational materials used in this study were developed with a focus on essential knowledge regarding MASLD, they were not sufficient to change the management patterns of the specialists.

Our results can potentially be applied to digital therapeutics. There is also a high demand for ongoing MASLD management among patients. The use of digital therapeutics could be a solution for efficient utilization of physicians’ consultation time as well as to enhance their motivation. In a web-based intervention study involving patients with MASLD, the most frequently accessed modules were “Welcome to the Program,” “Understanding MASLD,” and “Food and MASLD,” with 37% of the patients requesting access to a lifestyle coach (24). Few studies have verified the effectiveness of MASLD educational training programs for PCPs (11, 25). In 2021, a team of European collaborators developed a continuing medical education program on MASLD/MASH tailored to primary care settings in Europe^2^ (11). This online training program, conducted for 28 Greek doctors over a period of 1 month, achieved high satisfaction among 96% of the PCPs, with 62% reporting a significant positive change in clinical practice. The participating doctors expressed strong interest in educational materials that are accessible via mobile applications. However, because most participants in this study were approximately 40 years old and adept at using smartphones, it is challenging to assert that these results represent the needs of all doctors. A previous study showed a generally high demand for a mobile application for MASLD management, particularly among the elderly aged 60 and above (6). Therefore, it is plausible that older doctors are keen to engage in educational programs accessible through mobile applications as well. Furthermore, as digital therapeutics evolve, there has been a gradual increase in the clinical application of patient-targeted therapeutics across various medical fields (26–28). Thus, the development of digital therapeutics for MASLD management is essential, and further research is required for its effective clinical implementation. In Korea, the Korean Association for the Study of the Liver (KASL) provides continuous medical education on their website.^3^ The online education programs include “Case Studies,” “Guidelines,” “Factsheet,” and “Liver Disease White Paper” for healthcare providers as well as “Common Knowledge” and “Liver White Paper” for the general public, with information being regularly updated. Based on the findings of this study, leveraging social network services or smartphone applications to disseminate the high-quality materials provided by KASL to PCPs and specialists could significantly enhance the management of patients with MASLD.

This study has several limitations. First, because the physicians completed the surveys themselves, there may have been discrepancies between their responses and actual clinical practice. Second, although we were able to confirm whether doctors received educational materials, we could not assess the extent to which they utilized these resources. Third, we were unable to verify whether changes in the physicians’ knowledge and clinical practice translated into improved disease progression in patients with MASLD. Finally, we cannot guarantee whether the changes in PCPs’ practices observed in this study will be sustained over the long term.

In conclusion, providing appropriate education on MASLD for PCPs could contribute to a reduction in unnecessary referral rates and increase awareness of cardiovascular evaluation. There is a need to develop more comprehensive educational materials on MASLD, and subsequent research should focus on serially monitoring the management patterns of PCPs. Further studies should be conducted to determine whether actual changes have occurred in clinical practice.

## Data availability statement

The raw data supporting the conclusions of this article will be made available by the authors, without undue reservation.

## Ethics statement

The studies involving humans were approved by Institutional Review Board of Hanyang University Hospital. The studies were conducted in accordance with the local legislation and institutional requirements. The participants provided their written informed consent to participate in this study.

## Author contributions

J-HL: Writing – original draft, Visualization, Methodology, Investigation, Formal analysis, Data curation, Conceptualization. EY: Writing – original draft, Software, Methodology, Investigation, Formal analysis, Data curation, Conceptualization. JO: Writing – original draft, Data curation. KK: Writing – review & editing, Data curation. SA: Writing – review & editing, Validation, Supervision, Project administration, Methodology, Formal analysis, Conceptualization. DJ: Writing – review & editing, Supervision, Project administration, Methodology, Funding acquisition, Formal analysis, Conceptualization.

## Footnotes

^1^https://www.intermd.co.kr

^2^www.espcg.eu

^3^https://kasl.org
