# Supplementary material for: Barriers to care linkage and educational impact on unnecessary MASLD referrals
Source: Front Med (Lausanne). 2024 Jul 25;11:1407389. doi: 10.3389/fmed.2024.1407389 (PMC11309125; doi:10.3389/fmed.2024.1407389)

Supplementary Material

**Supplementary data 1.** a 21-item baseline questionnaire and a 16-item follow-up questionnaire

**A 21-item Baseline Questionnaire for Physician**

Metabolic dysfunction-associated steatotic liver disease causes chronic inflammation and fibrosis of liver tissue due to the accumulation of excess fat, increasing mortality from liver disease. In addition, as a risk factor for cardiovascular diseases such as angina, myocardial infarction, and stroke, it also increases mortality from cardiovascular diseases. The purpose of this questionnaire is to identify the current state and issues related to the management of metabolic dysfunction-associated steatotic liver disease by understanding the physician’s perspective on metabolic dysfunction-associated steatotic liver disease, and to devise a plan for improvement. All collected data will be kept confidential in accordance with the Personal Information Protection Act. Data analysis will be carried out utilizing the integrated opinion of all study participants, and will not be based on your responses alone.

**1. Among all patients that you see during one session, what percentage has metabolic dysfunction-associated steatotic liver disease?**

1. None
2. Between 0 – 25%
3. Between 25 – 50%
4. Between 50 – 75%
5. Between 75 – 100%

**2. Among all patients with metabolic dysfunction-associated steatotic liver disease that you see during one session, what percentage had been diagnosed incidentally during a health check-up?**

1. None
2. Between 0 – 25%
3. Between 25 – 50%
4. Between 50 – 75%
5. Between 75 – 100%

※Tests for metabolic dysfunction-associated steatotic liver disease include screening tests(e.g., abdominal ultrasound) and tests that evaluate the severity of liver fibrosis(e.g., fibrosis index-4, MASLD fibrosis score, Fibroscan®, etc).

The following questions aim to understand your current criteria and practice in conducting screening tests for metabolic dysfunction-associated steatotic liver disease and assessment of the severity of liver fibrosis.

**3. How often do you perform screening tests such as abdominal ultrasound for metabolic dysfunction-associated steatotic liver disease on the following patients?**

|  | Almost always  (75–100%) | Frequently (50–74%) | Sometimes (25–49%) | Rarely  (1–24%) | Never  (0%) |
| --- | --- | --- | --- | --- | --- |
| All patients |  |  |  |  |  |
| Patients with abnormal AST or ALT levels |  |  |  |  |  |
| Obese patients |  |  |  |  |  |
| Diabetic patients |  |  |  |  |  |
| Dyslipidemic patients |  |  |  |  |  |
| Patients with metabolic syndrome |  |  |  |  |  |
| Patients with ischemic heart disease |  |  |  |  |  |
| Patients who have had a cerebral stroke |  |  |  |  |  |

**4. How often do you order the following tests for patients with suspected metabolic dysfunction-associated steatotic liver disease?**

|  | Almost always  (75–100%) | Frequently  (50–74%) | Sometimes  (25–49%) | Rarely  (1–24%) | Never  (0%) |
| --- | --- | --- | --- | --- | --- |
| AST or ALT |  |  |  |  |  |
| Abdominal ultrasound |  |  |  |  |  |
| Imaging test for liver fibrosis(Fibroscan®, etc) |  |  |  |  |  |
| Hematological assessment of liver fibrosis(Fibrosis index-4, MASLD fibrosis score, etc) |  |  |  |  |  |
| Blood glucose test |  |  |  |  |  |
| Lipid panel |  |  |  |  |  |

**5. What factors do you consider for liver fibrosis evaluation in patients with metabolic dysfunction-associated steatotic liver disease? (Multiple response allowed)**

1. Never considered
2. Old age
3. Abnormal AST or ALT levels
4. Obesity
5. Diabetes
6. Dyslipidemia
7. Metabolic syndrome
8. Ischemic cardiac disease
9. Cerebral stroke

**6.** **Have you ever heard of, or do you currently use serologic markers of liver fibrosis (fibrosis index-4, MASLD fibrosis score, etc.) to diagnose liver fibrosis or to stratify its severity in patients with metabolic dysfunction-associated steatotic liver disease?**

1. Never heard of it.
2. Have heard of it, but do not currently use.
3. Have heard of it, and currently using.

**7. How do you treat a patient who is incidentally found to have metabolic dysfunction-associated steatotic liver disease on ultrasound? (Multiple responses allowed)**

1. Briefly mention that the patient has metabolic dysfunction-associated steatotic liver disease and do not proceed with any additional measures
2. Refer patient to the gastroenterology or a tertiary hospital
3. Take no additional measure if patient has normal AST and ALT levels.
4. Mention that the patient has fatty liver and recommend lifestyle modification
5. Order additional tests (blood glucose test, lipid panel, etc.) for metabolic syndrome (diabetes mellitus, dyslipidemia, etc.)
6. Order additional tests regarding the liver fibrosis
7. Order additional tests to determine whether patient also has cardiovascular disease

**8. What kind of education do you provide patients who have metabolic dysfunction-associated steatotic liver disease? (Multiple responses allowed)**

1. Provide no further explanation
2. Explain the increased risk of disease progression into cirrhosis or liver cancer
3. Explain the increased risk of metabolic diseases (diabetes mellitus, dyslipidemia, etc.)
4. Explain the increased risk of ischemic heart disease
5. Explain the increased risk of cerebral stroke
6. Explain the increased risk of extrahepatic cancer

**9. What type of lifestyle modification do you recommend patients who have metabolic dysfunction-associated steatotic liver disease? (Multiple responses allowed)**

1. Provide no education on lifestyle modification (Go to Question 13)
2. Weight loss (Go to Question 9-1)
3. Dietary education
4. Education on physical activity

**9-1. In general, what percent weight loss do you recommend to overweight or obese patients with metabolic dysfunction-associated steatotic liver disease?**

1. Less than 5%
2. Between 5 - 10%
3. Between 10 - 15%
4. Between 15 - 20%
5. More than 20%
6. Give no mention of a percentage

**10. How much time do you allot towards educating patients with metabolic dysfunction-associated steatotic liver disease on lifestyle modification?**

1. Less than a minute
2. Between 1 - 3 minutes
3. Between 3 - 5 minutes
4. More than 5 minutes

**11. What type of medications do you currently use for the management of metabolic dysfunction-associated steatotic liver disease? (Multiple responses allowed)**

1. No use of medication
2. Use of diabetes medication within the range of approved use (Metformin, Pioglitazone, etc.s)
3. Off-label use of diabetes medication [Dulaglutide(Trulicity®), Liraglutide(Victoza®), etc.]
4. Use of anti-obesity drugs [Phentermine/Topiramate(Qsymia®), Liraglutide(Saxenda®), Bupropion/Naltrexone (Contrave®), etc.]
5. Use of liver disease drugs [Silymarin(Legalon®), DDB complex(Godex®, Pennel®), etc.]
6. Use of choleretic drugs (Ursodeoxycholic acid(Ursa®)
7. Use of dyslipidemia drugs (Atorvastatin, Rosuvastatin, etc.)
8. Vitamin E
9. Intestinal drugs (Lactic acid bacteria)

**12. In which case do you recommend follow-up visits for patients with metabolic dysfunction-associated steatotic liver disease? (Multiple responses allowed)**

1. Give no recommendation (Go to question 15)
2. If weight loss is necessary
3. If AST or ALT level is abnormal
4. If the patient has comorbid metabolic disorders (diabetes mellitus, dyslipidemia, etc.)
5. If the severity of liver fibrosis is high
6. If the patient has risk of ischemic cardiovascular disease or cerebral stroke

**13. What type of test is utilized for the follow-up of patients with metabolic dysfunction-associated steatotic liver disease? (Multiple responses allowed)**

1. AST and ALT
2. Abdominal ultrasound
3. Imaging test for liver fibrosis (eg., Transient elastography [Fibroscan®])
4. Serologic markers of liver fibrosis (Serological test panel)

**14. How often do you follow-up with patients with metabolic dysfunction-associated steatotic liver disease?**

1. Less than 1 month
2. Between 1 – 3 months
3. Between 3 – 6 months
4. Between 6 and 12 months
5. More than 12 months

**15. From a physician’s perspective, what hinders the consistent management of patients with metabolic dysfunction-associated steatotic liver disease?**

| Factor | Strongly agree  (5points) | Agree  (4points) | Neutral  (3points) | Disagree  (2points) | Strongly Disagree  (1point) |
| --- | --- | --- | --- | --- | --- |
| Short consultation time |  |  |  |  |  |
| Lack of adequate educational materials |  |  |  |  |  |
| Not my primary area of practice |  |  |  |  |  |
| Patients’ low compliance |  |  |  |  |  |
| Lack of appropriate medication |  |  |  |  |  |
| Fee for this service does not exist |  |  |  |  |  |

**16. From the patients’ perspective, what hinders lifestyle modification? (Multiple responses allowed)**

1. Patients don’t consider metabolic dysfunction-associated steatotic liver disease a grave illness.
2. Patients do not know how to pursue lifestyle modification
3. Patients don’t have enough time for lifestyle modification
4. Patients lack motivation to pursue lifestyle modification
5. Physicians do not proactively provide education
6. There exists no program that supports ongoing management

**17. What do you think is necessary for long-term and efficient management of metabolic dysfunction-associated steatotic liver disease when you see patients? (Multiple responses allowed)**

1. Improving awareness of metabolic dysfunction-associated steatotic liver disease
2. Education on lifestyle modification
3. Development of a lifestyle modification management program
4. Development of treatment for metabolic dysfunction-associated steatotic liver disease
5. Officially setting fees for relevant consultation during patient visits
6. Don’t know

**18.** **What is the approximate level of understanding of incoming patients with metabolic dysfunction-associated steatotic liver disease regarding their disease?**

1. Very knowledgeable (5points)
2. Knowledgeable (4points)
3. Average (3points)
4. Not knowledgeable(2points)
5. Have no knowledge at all (1point)

**19. What is your source of knowledge regarding metabolic dysfunction-associated steatotic liver disease? (Multiple responses allowed)**

1. At this time, I don’t seek additional knowledge on metabolic dysfunction-associated steatotic liver disease
2. Guideline or medical textbook on metabolic dysfunction-associated steatotic liver disease
3. Medical journals
4. Colleagues
5. Web portal (Blog, cafe, etc.)
6. Youtube

**20. (Respond if you are NOT a GI physician) What percent of your patients with metabolic dysfunction-associated steatotic liver disease do you refer to a GI specialist?**

1. Refer all of them to a GI specialist or a tertiary hospital
2. Between 0 – 25%
3. Between 25 – 50%
4. Between 50 – 75%
5. Between 75 – 100%
6. Treat them without referral (End of questionnaire)

**21. (Respond if you are NOT a GI physician) In which case do you refer your patients with metabolic dysfunction-associated steatotic liver disease to gastroenterology or a tertiary hospital? (Multiple responses allowed)**

1. Immediately refer to GI once hepatic steatosis is confirmed
2. If AST or ALT level is abnormal
3. If the patient’s hepatic steatosis is severe
4. If there is concern regarding the progression of liver fibrosis or cirrhosis (severe liver fibrosis, low platelet count, etc.)
5. If patient has comorbid metabolic disorders (diabetes mellitus, dyslipidemia, etc.)
6. If patient has comorbid ischemic cardiovascular disease or cerebral stroke
7. When considering a variety of treatment options for metabolic dysfunction-associated steatotic liver disease
8. By request of patients or their families

※ This research is being conducted as a project funded by the Korea Centers for Disease Control and Prevention. If you would like information on the management of metabolic dysfunction-associated steatotic liver disease, we would be glad to send you information via email on a regular basis for two months. For those who receive information on a regular basis, we plan to distribute a 2-month follow-up questionnaire and provide additional points. Do you agree to subscribe to receive information on metabolic dysfunction-associated steatotic liver disease on a regular basis?

① Yes.

② No.

**A 16-item Follow-up Questionnaire for Physician**

This follow-up survey is designed to assess the impact of a 7-week educational program on metabolic dysfunction-associated steatotic liver disease. It aims to determine whether this program enhances physicians' awareness of metabolic dysfunction-associated steatotic liver disease and helps address the challenges in its actual management. The survey will be administered to those who consented to participate in this follow-up during the initial survey phase. All provided information will be kept confidential, in compliance with personal information protection laws. Furthermore, the responses will be analyzed as the aggregate opinion of all participating physicians, rather than on an individual basis.

※Tests for metabolic dysfunction-associated steatotic liver disease include screening tests(e.g., abdominal ultrasound) and tests that evaluate the severity of liver fibrosis(e.g., fibrosis index-4, MASLD fibrosis score, Fibroscan®, etc).

The following questions aim to understand your current criteria and practice in conducting screening tests for metabolic dysfunction-associated steatotic liver disease and assessment of the severity of liver fibrosis.

**1. How often do you perform screening tests such as abdominal ultrasound for metabolic dysfunction-associated steatotic liver disease on the following patients?**

|  | Almost always  (75–100%) | Frequently (50–74%) | Sometimes (25–49%) | Rarely  (1–24%) | Never  (0%) |
| --- | --- | --- | --- | --- | --- |
| All patients |  |  |  |  |  |
| Patients with abnormal AST or ALT levels |  |  |  |  |  |
| Obese patients |  |  |  |  |  |
| Diabetic patients |  |  |  |  |  |
| Dyslipidemic patients |  |  |  |  |  |
| Patients with metabolic syndrome |  |  |  |  |  |
| Patients with ischemic heart disease |  |  |  |  |  |
| Patients who have had a cerebral stroke |  |  |  |  |  |

**2. How often do you order the following tests for patients with suspected metabolic dysfunction-associated steatotic liver disease?**

|  | Almost always  (75–100%) | Frequently  (50–74%) | Sometimes  (25–49%) | Rarely  (1–24%) | Never  (0%) |
| --- | --- | --- | --- | --- | --- |
| AST or ALT |  |  |  |  |  |
| Abdominal ultrasound |  |  |  |  |  |
| Imaging test for liver fibrosis(Fibroscan®, etc) |  |  |  |  |  |
| Hematological assessment of liver fibrosis(Fibrosis index-4, MASLD fibrosis score, etc) |  |  |  |  |  |
| Blood glucose test |  |  |  |  |  |
| Lipid panel |  |  |  |  |  |

**3. What factors do you consider for liver fibrosis evaluation in patients with metabolic dysfunction-associated steatotic liver disease? (Multiple response allowed)**

1. Never considered
2. Old age
3. Abnormal AST or ALT levels
4. Obesity
5. Diabetes
6. Dyslipidemia
7. Metabolic syndrome
8. Ischemic cardiac disease
9. Cerebral stroke

**4.** **Have you ever heard of, or do you currently use serologic markers of liver fibrosis (fibrosis index-4, MASLD fibrosis score, etc.) to diagnose liver fibrosis or to stratify its severity in patients with metabolic dysfunction-associated steatotic liver disease?**

1. Never heard of it.
2. Have heard of it, but do not currently use.
3. Have heard of it, and currently using.

**5. How do you treat a patient who is incidentally found to have metabolic dysfunction-associated steatotic liver disease on ultrasound? (Multiple responses allowed)**

1. Briefly mention that the patient has metabolic dysfunction-associated steatotic liver disease and do not proceed with any additional measures
2. Refer patient to the gastroenterology or a tertiary hospital
3. Take no additional measure if patient has normal AST and ALT levels.
4. Mention that the patient has fatty liver and recommend lifestyle modification
5. Order additional tests (blood glucose test, lipid panel, etc.) for metabolic syndrome (diabetes mellitus, dyslipidemia, etc.)
6. Order additional tests regarding the liver fibrosis
7. Order additional tests to determine whether patient also has cardiovascular disease

**6. What kind of education do you provide patients who have metabolic dysfunction-associated steatotic liver disease? (Multiple responses allowed)**

1. Provide no further explanation
2. Explain the increased risk of disease progression into cirrhosis or liver cancer
3. Explain the increased risk of metabolic diseases (diabetes mellitus, dyslipidemia, etc.)
4. Explain the increased risk of ischemic heart disease
5. Explain the increased risk of cerebral stroke
6. Explain the increased risk of extrahepatic cancer

**7. What type of lifestyle modification do you recommend patients who have metabolic dysfunction-associated steatotic liver disease? (Multiple responses allowed)**

1. Provide no education on lifestyle modification (Go to Question 10)
2. Weight loss (Go to Question 8)
3. Dietary education
4. Education on physical activity

**8. In general, what percent weight loss do you recommend to overweight or obese patients with metabolic dysfunction-associated steatotic liver disease?**

1. Less than 5%
2. Between 5 - 10%
3. Between 10 - 15%
4. Between 15 - 20%
5. More than 20%
6. Give no mention of a percentage

**9. How much time do you allot towards educating patients with metabolic dysfunction-associated steatotic liver disease on lifestyle modification?**

1. Less than a minute
2. Between 1 - 3 minutes
3. Between 3 - 5 minutes
4. More than 5 minutes

**10. What type of test is utilized for the follow-up of patients with metabolic dysfunction-associated steatotic liver disease (Multiple responses allowed)**

1. AST and ALT
2. Abdominal ultrasound
3. Imaging test for liver fibrosis (eg., Transient elastography [Fibroscan®])
4. Serologic markers of liver fibrosis (Serological test panel)

**11. From a physician’s perspective, what hinders the consistent management of patients with metabolic dysfunction-associated steatotic liver disease?**

| Factor | Strongly agree  (5points) | Agree  (4points) | Neutral  (3points) | Disagree  (2points) | Strongly Disagree  (1point) |
| --- | --- | --- | --- | --- | --- |
| Short consultation time |  |  |  |  |  |
| Lack of adequate educational materials |  |  |  |  |  |
| Not my primary area of practice |  |  |  |  |  |
| Patients’ low compliance |  |  |  |  |  |
| Lack of appropriate medication |  |  |  |  |  |
| Fee for this service does not exist |  |  |  |  |  |

**12. From the patients’ perspective, what hinders lifestyle modification? (Multiple responses allowed)**

1. Patients don’t consider metabolic dysfunction-associated steatotic liver disease a grave illness.
2. Patients do not know how to pursue lifestyle modification
3. Patients don’t have enough time for lifestyle modification
4. Patients lack motivation to pursue lifestyle modification
5. Physicians do not proactively provide education
6. There exists no program that supports ongoing management

**13. What do you think is necessary for long-term and efficient management of metabolic dysfunction-associated steatotic liver disease when you see patients? (Multiple responses allowed)**

1. Improving awareness of metabolic dysfunction-associated steatotic liver disease
2. Education on lifestyle modification
3. Development of a lifestyle modification management program
4. Development of treatment for metabolic dysfunction-associated steatotic liver disease
5. Officially setting fees for relevant consultation during patient visits
6. Don’t know

**14.** **What is the approximate level of understanding of incoming patients with metabolic dysfunction-associated steatotic liver disease regarding their disease?**

1. Very knowledgeable (5points)
2. Knowledgeable (4points)
3. Average (3points)
4. Not knowledgeable(2points)
5. Have no knowledge at all (1point)

**15. (Respond if you are NOT a GI physician) In which case do you refer your patients with metabolic dysfunction-associated steatotic liver disease to gastroenterology or a tertiary hospital? (Multiple responses allowed)**

1. Immediately refer to GI once hepatic steatosis is confirmed
2. If AST or ALT level is abnormal
3. If the patient’s hepatic steatosis is severe
4. If there is concern regarding the progression of liver fibrosis or cirrhosis (severe liver fibrosis, low platelet count, etc.)
5. If patient has comorbid metabolic disorders (diabetes mellitus, dyslipidemia, etc.)
6. If patient has comorbid ischemic cardiovascular disease or cerebral stroke
7. When considering a variety of treatment options for metabolic dysfunction-associated steatotic liver disease
8. By request of patients or their families

**16. After receiving education about metabolic dysfunction-associated steatotic liver disease (MASLD), how has your approach to treatment changed compared to before? Please select three responses that best describe the changes you've experienced, in order of significance, starting with the most impactful.**

1. Increased time spent educating patients on lifestyle habits
2. More frequent explanations about the risk of ischemic heart disease
3. Increased discussions about the potential for stroke
4. More detailed explanations about the increased risk of liver cancer
5. Increased emphasis on the risk of cancers other than liver cancer
6. Detailed explanations provided about the appropriate degree of weight loss
7. Clarifications given on the appropriate intensity and frequency of exercise

**Supplementary data 2.** MASLD educational materials which was disseminated online weekly from the first to seventh week

**First week**


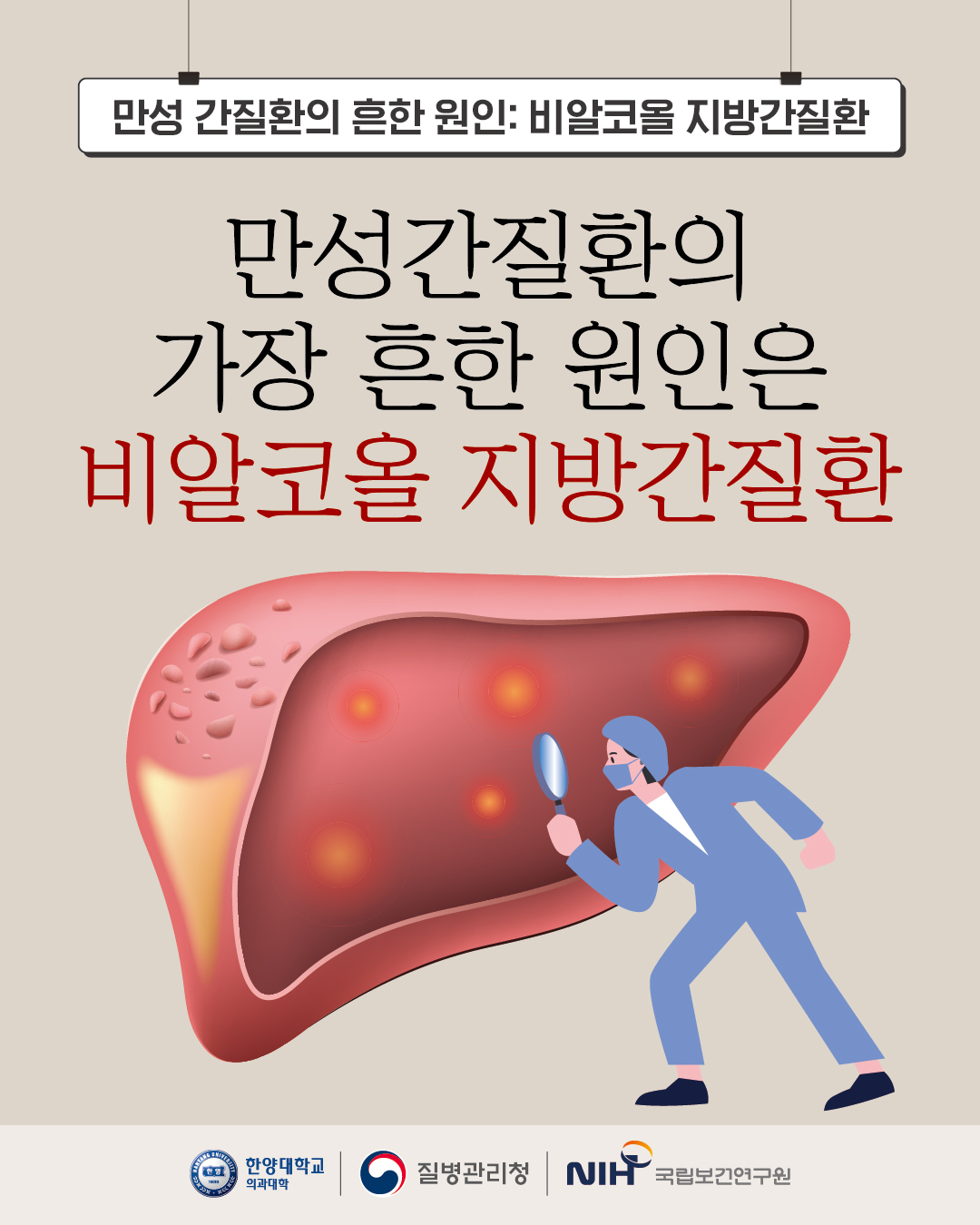

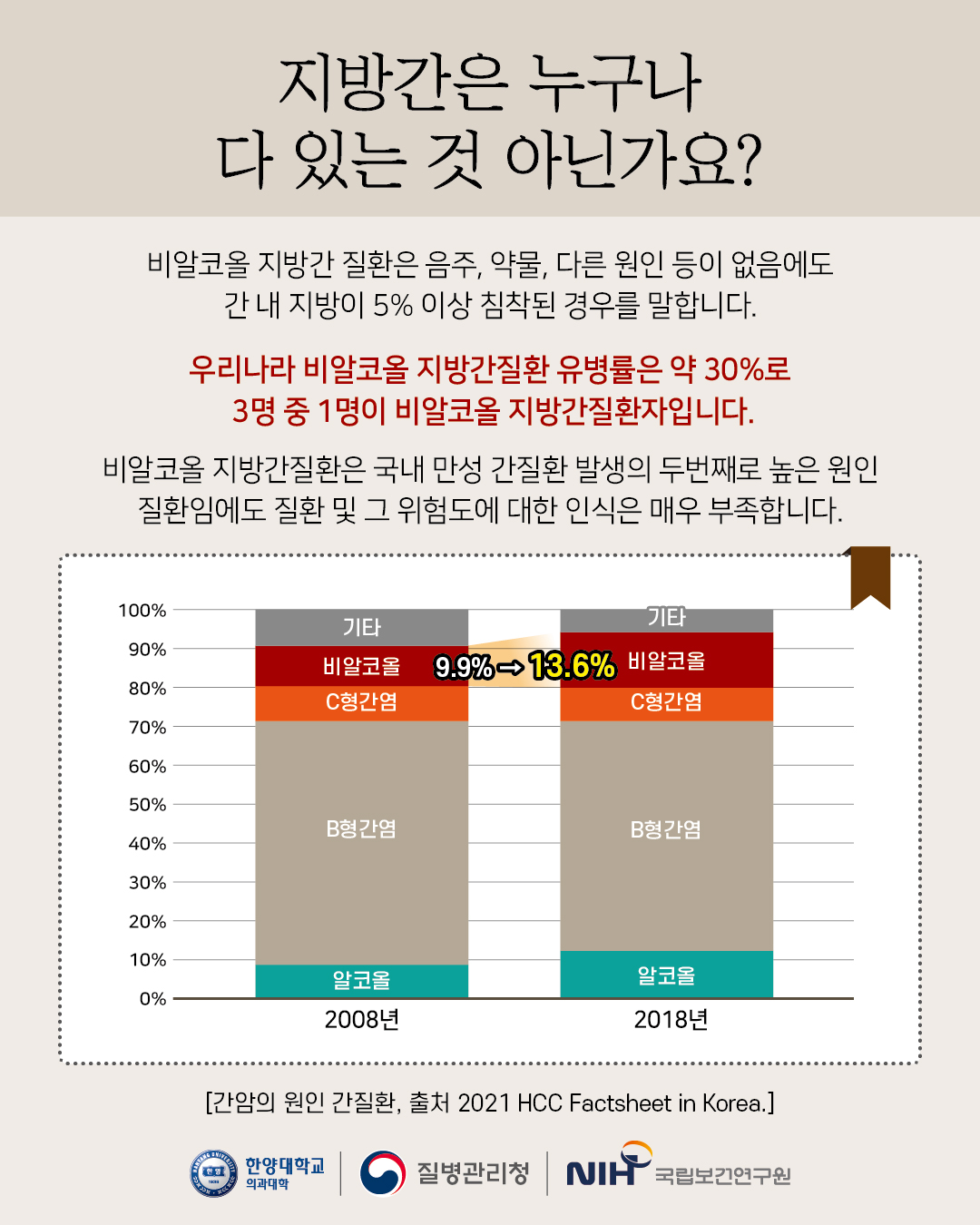

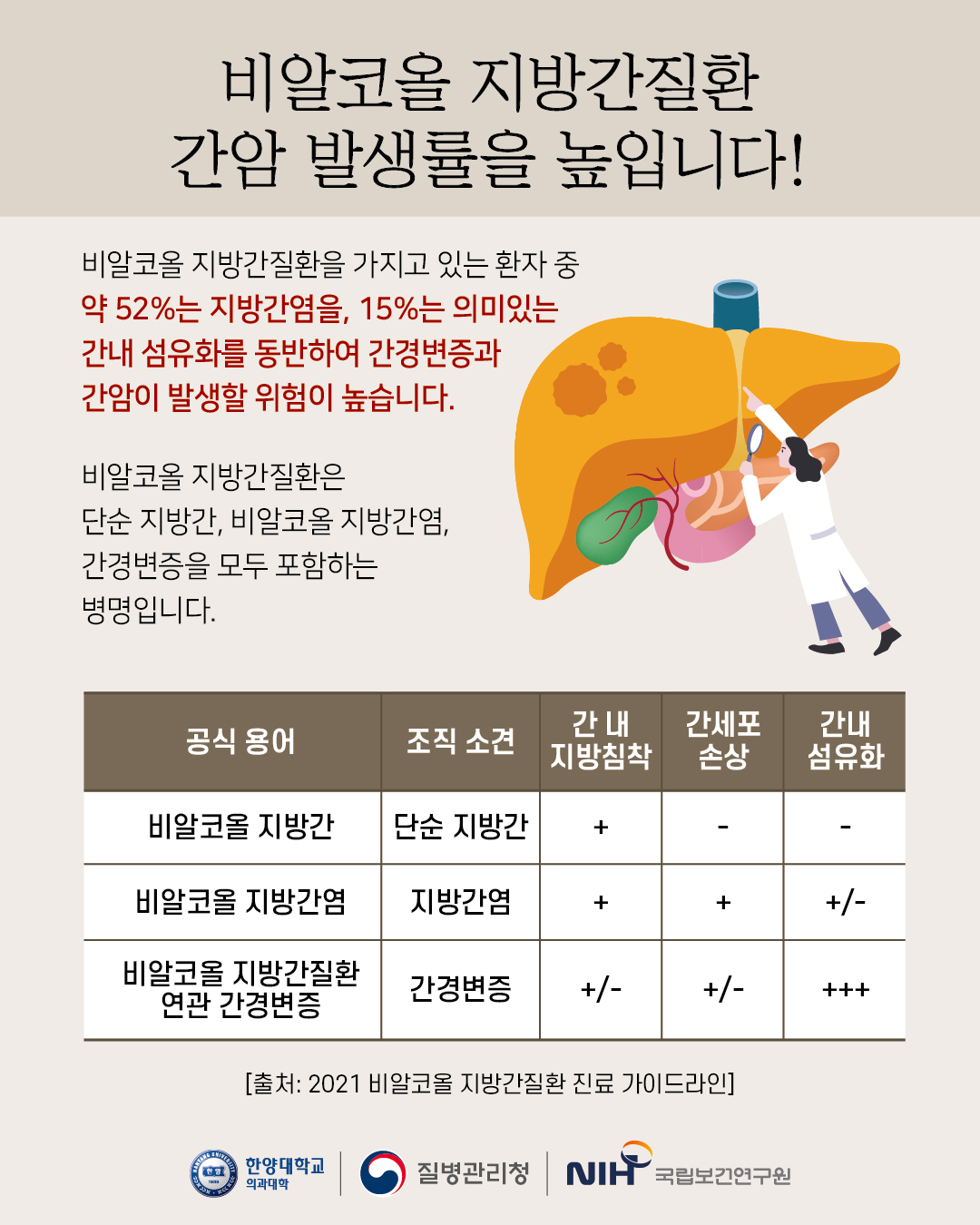

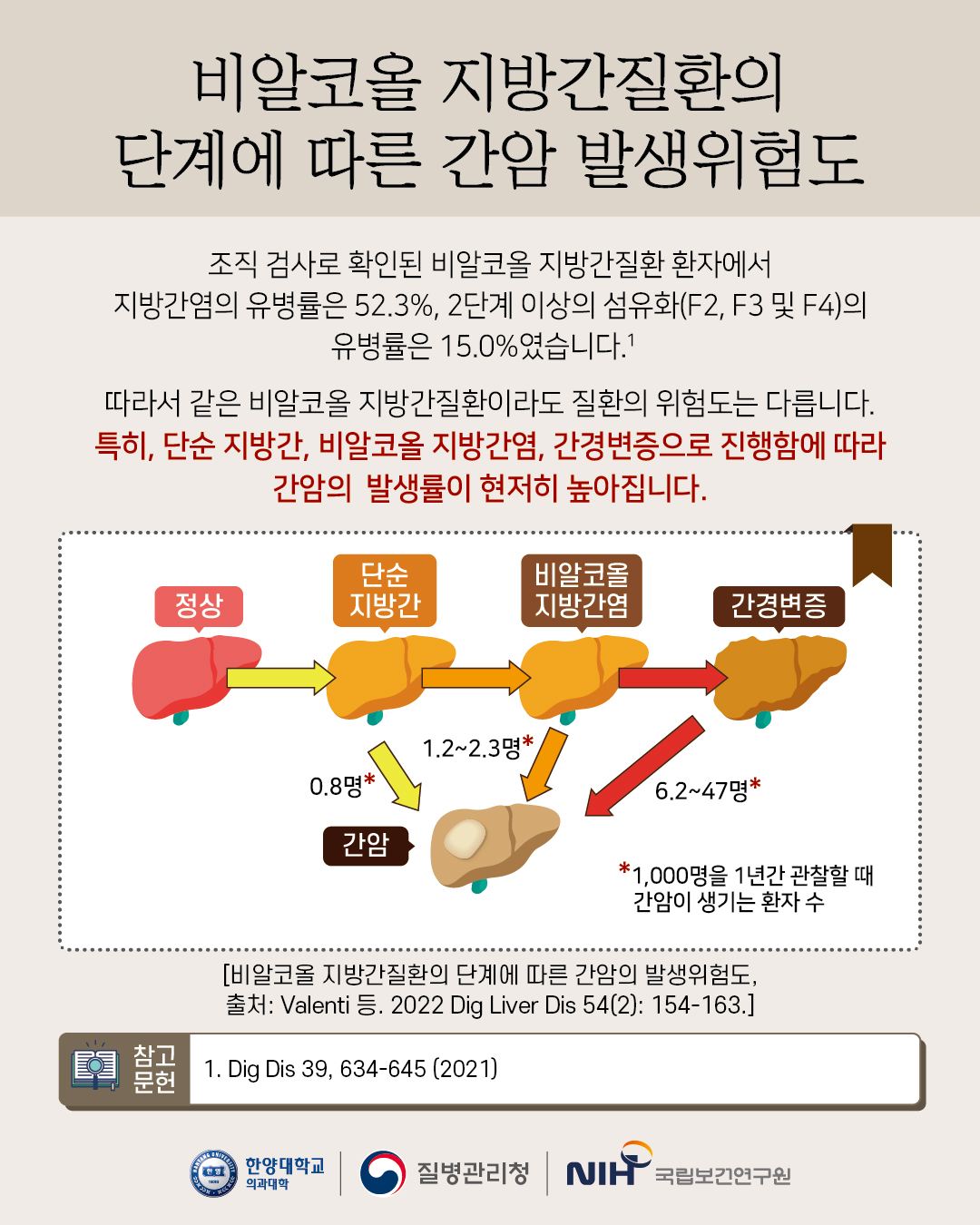

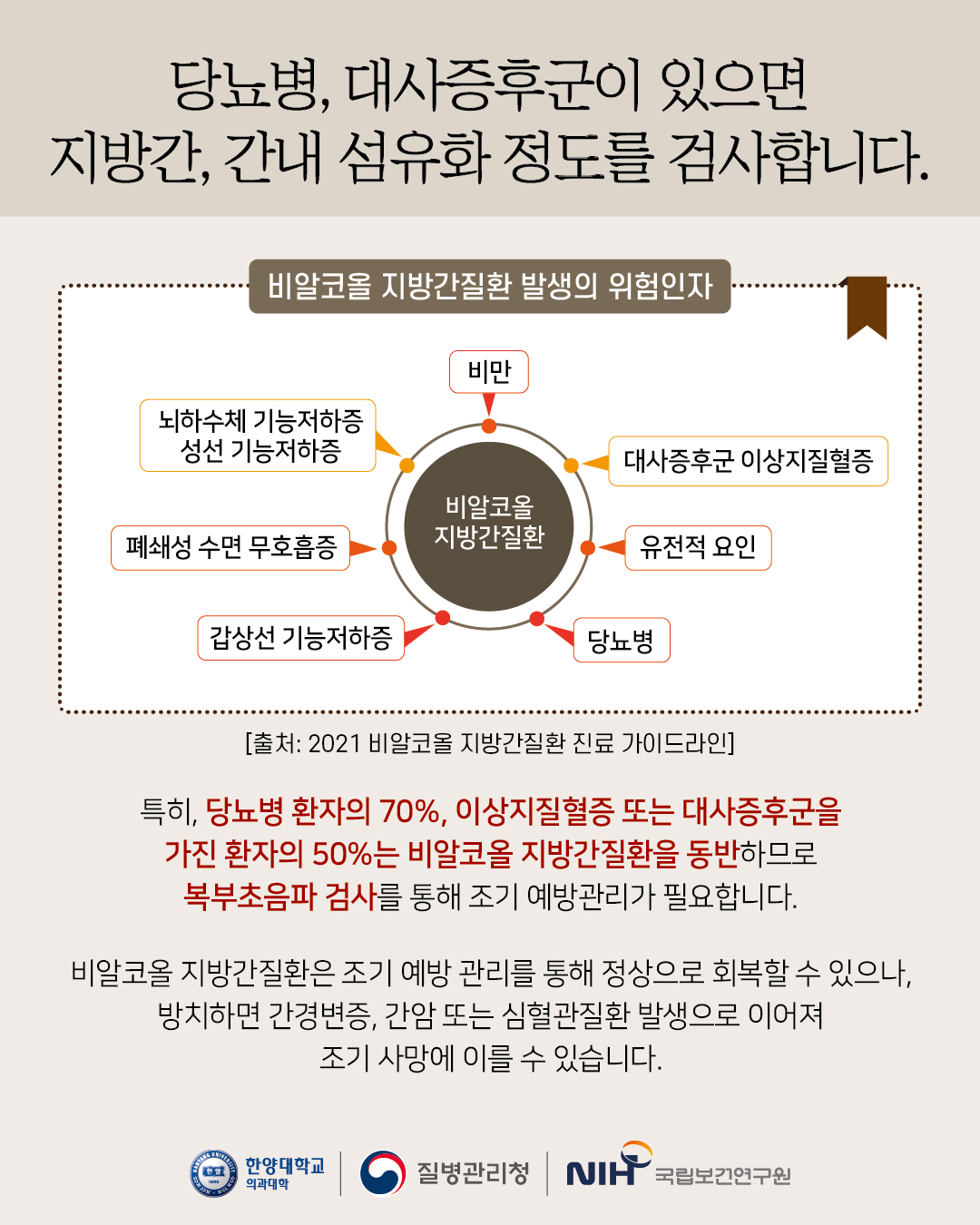

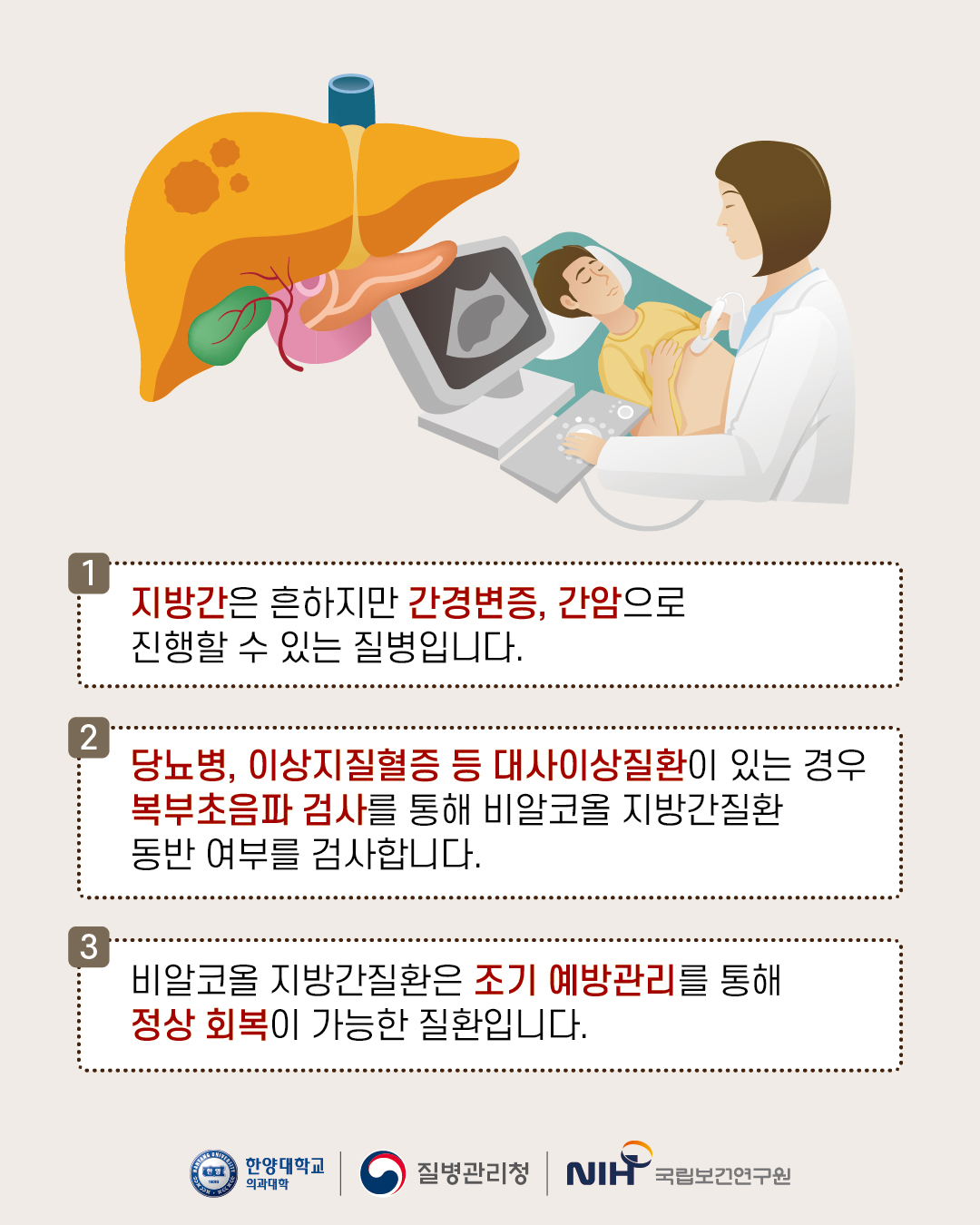


**Second week**


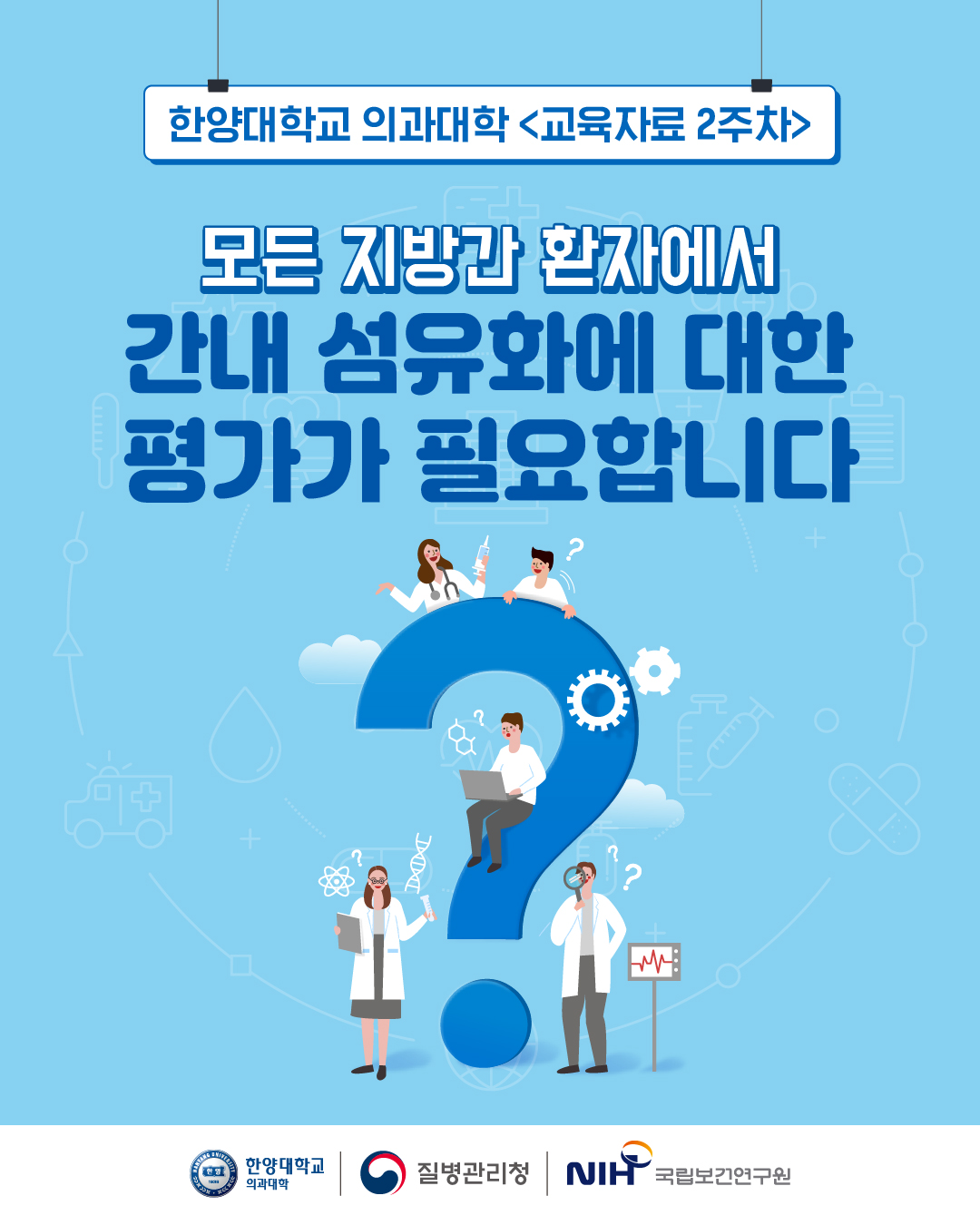


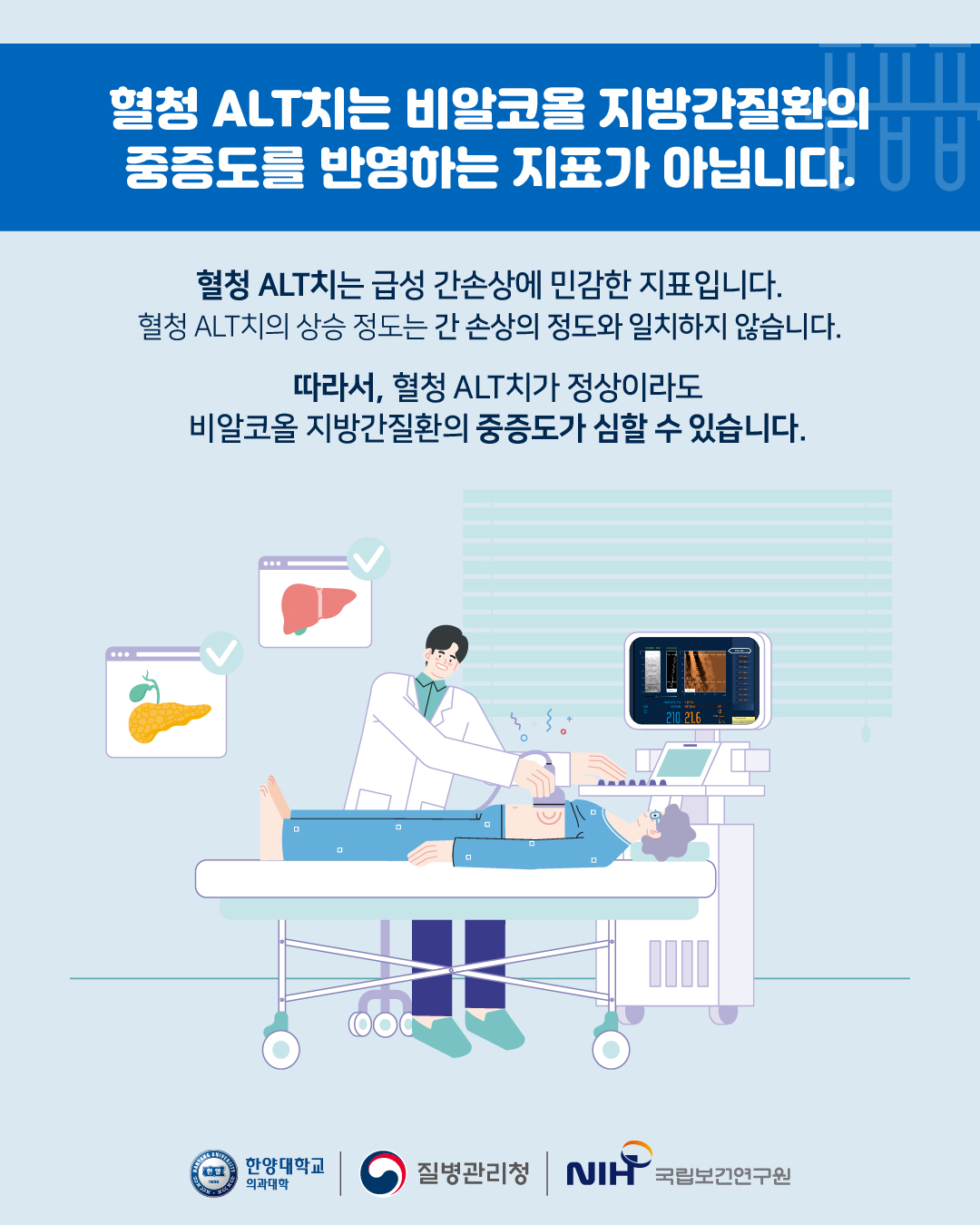


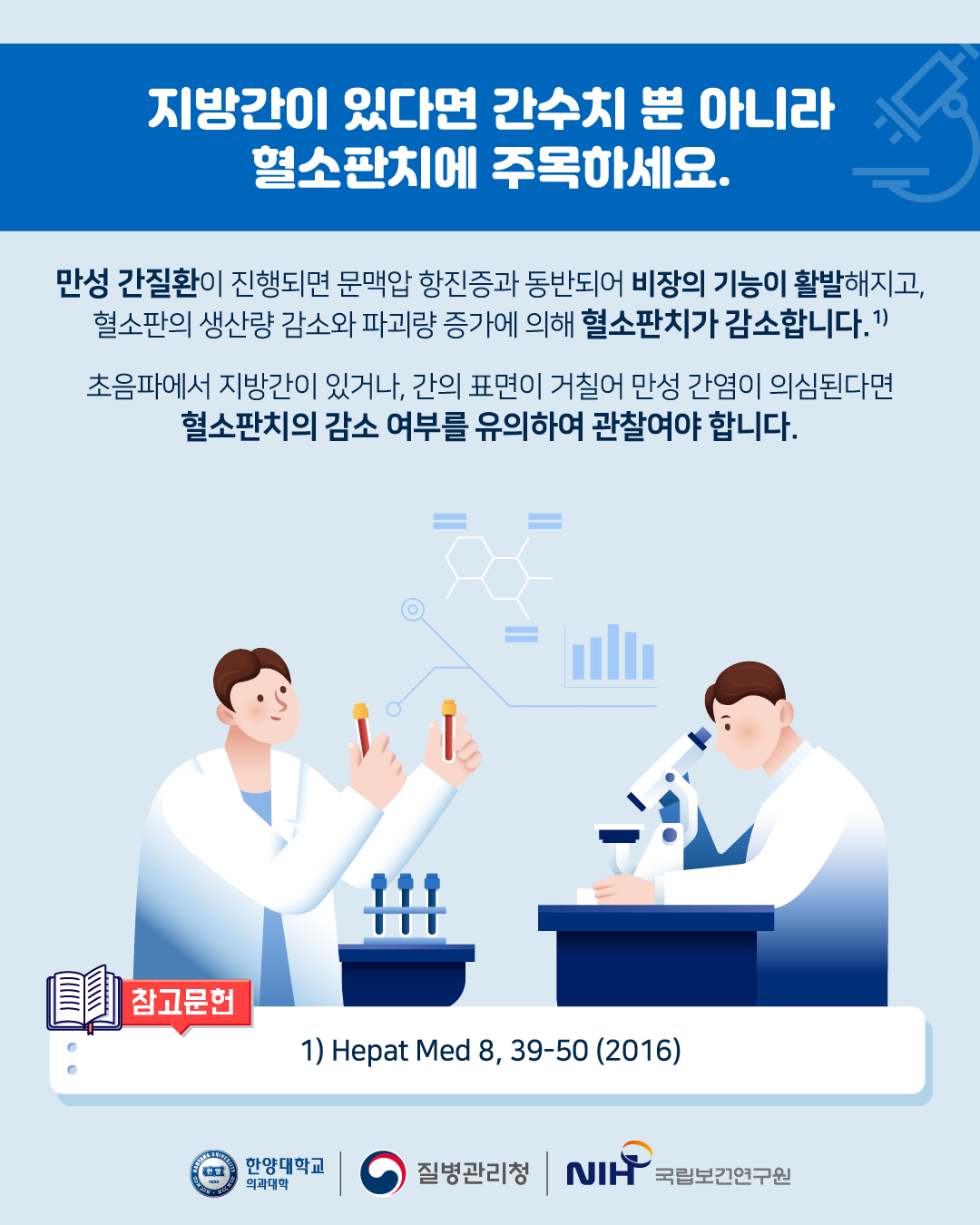


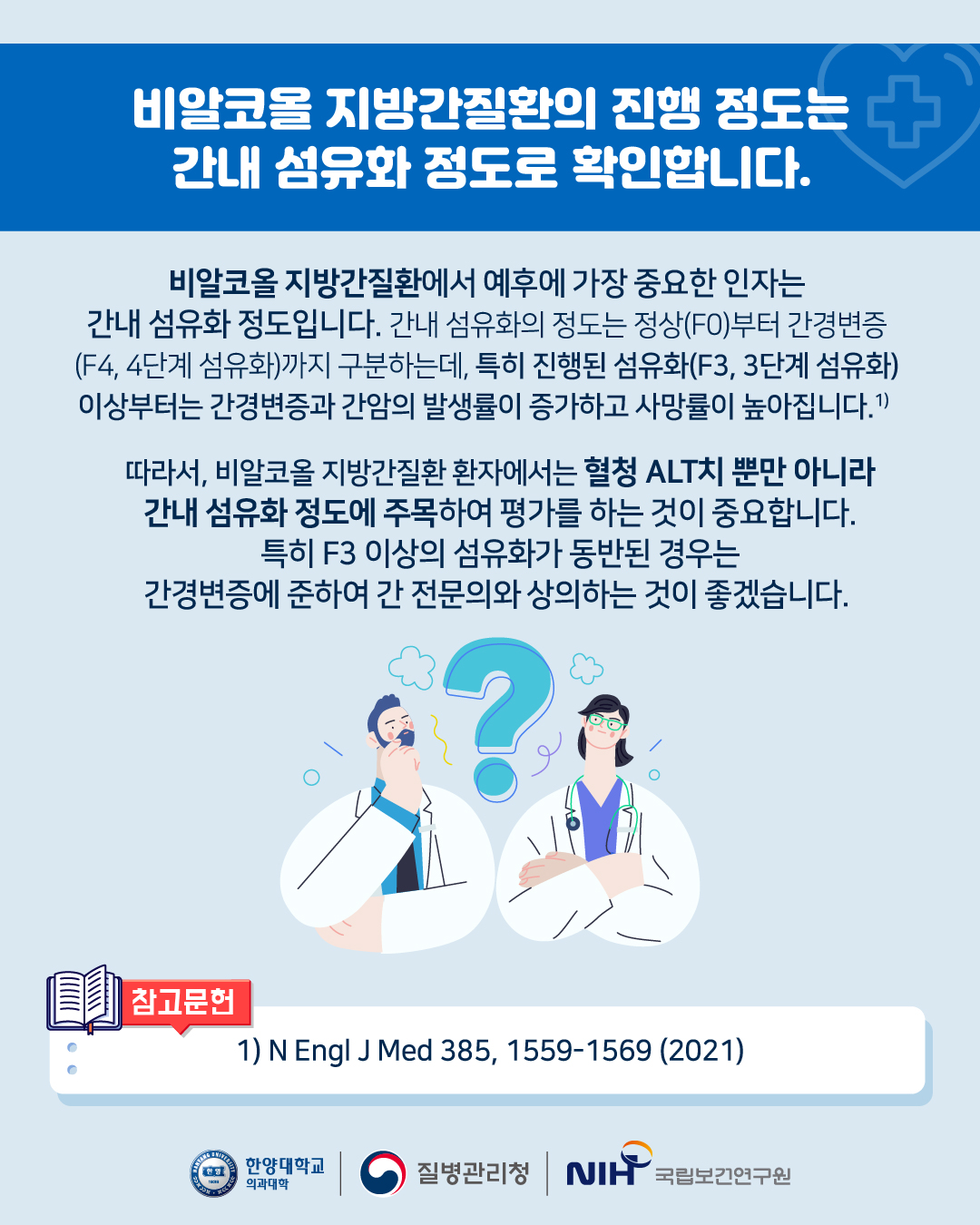


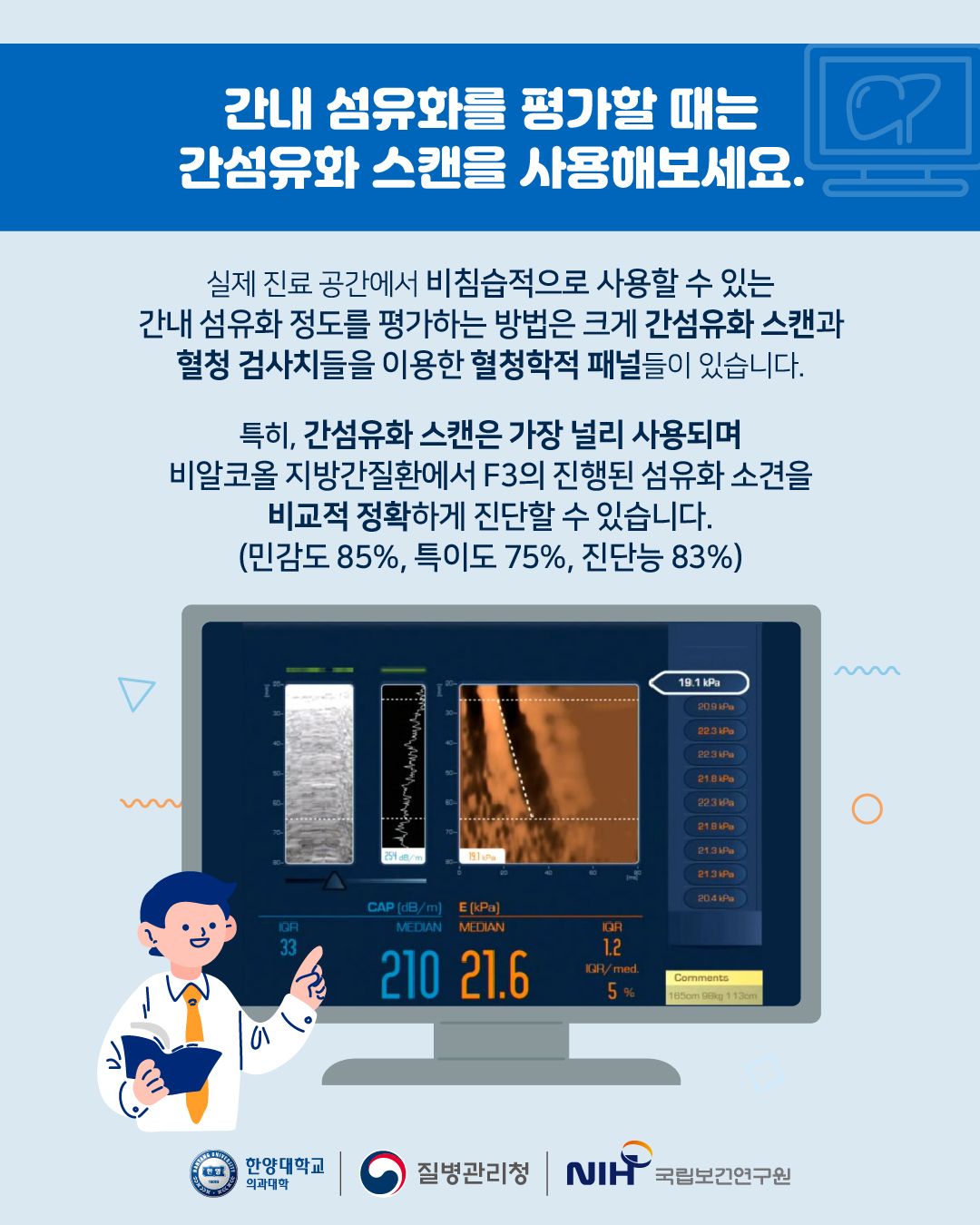


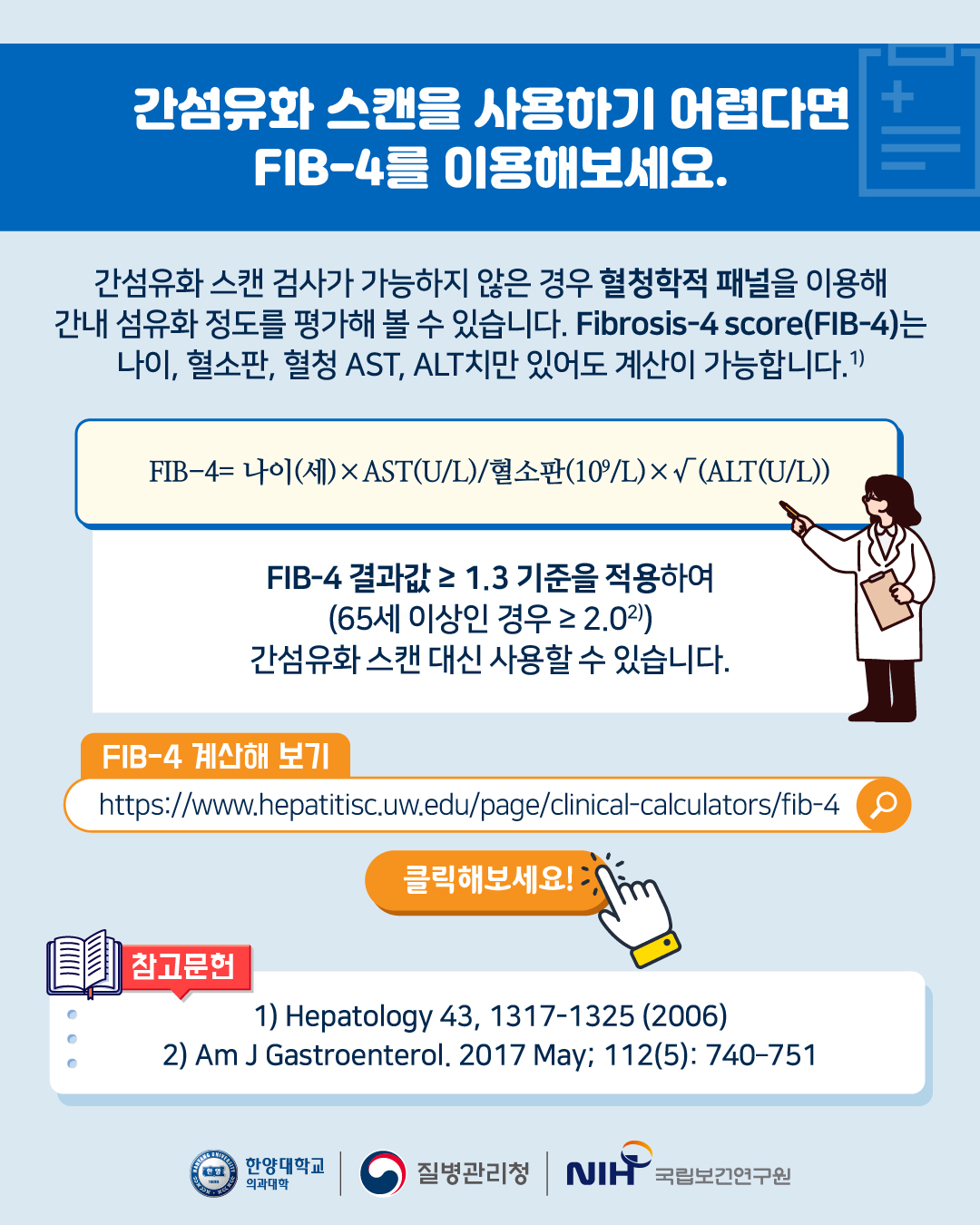


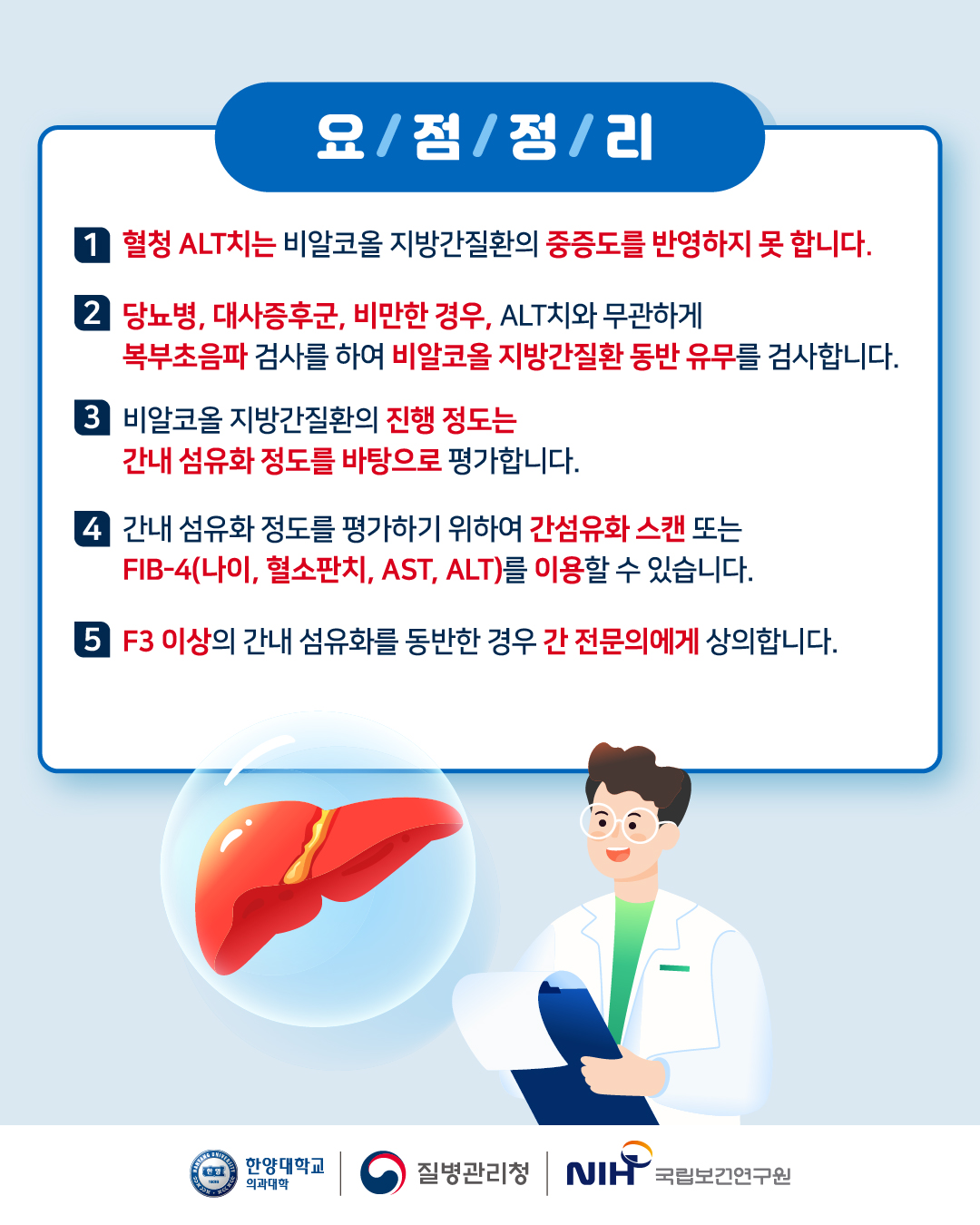


**Third week**


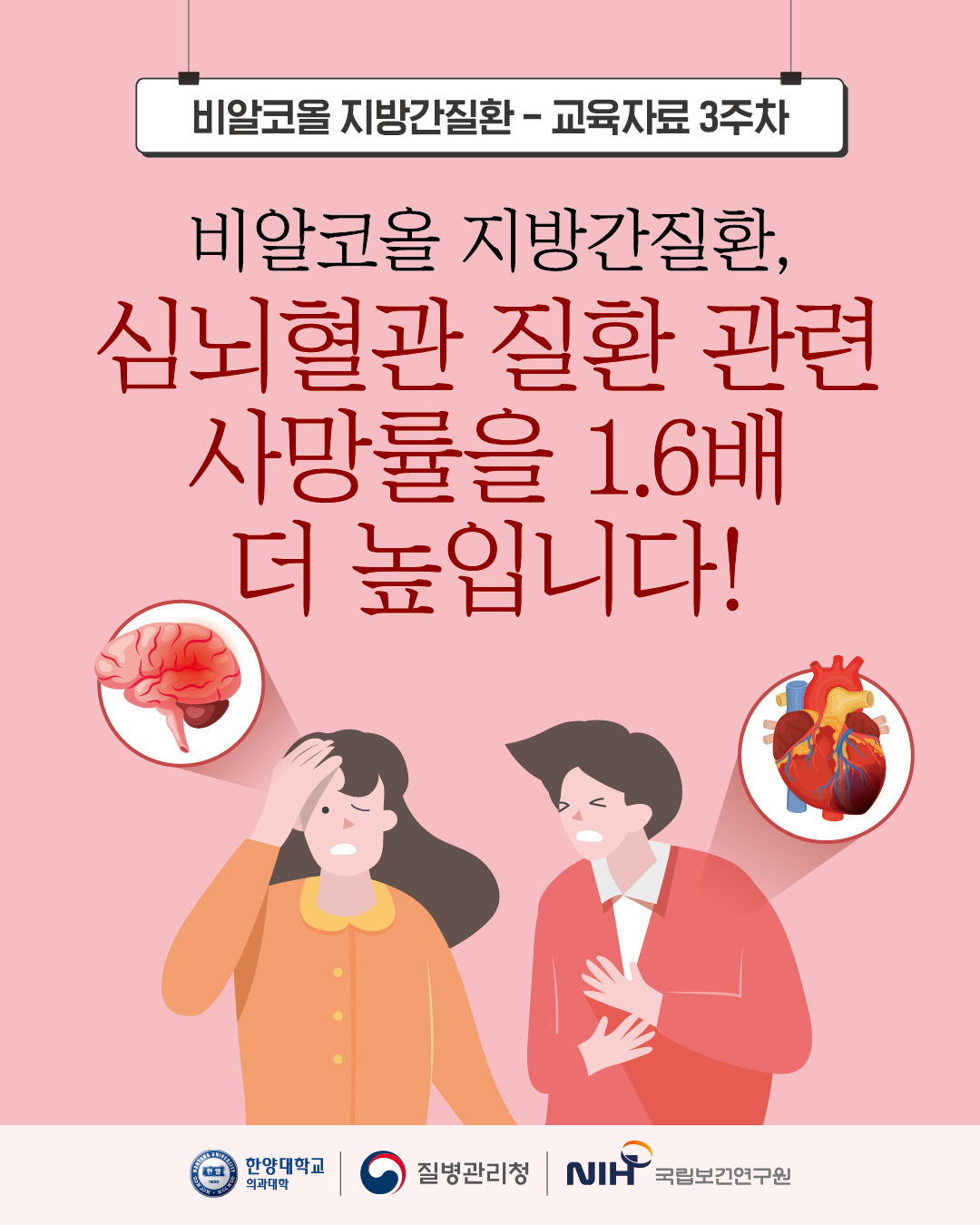


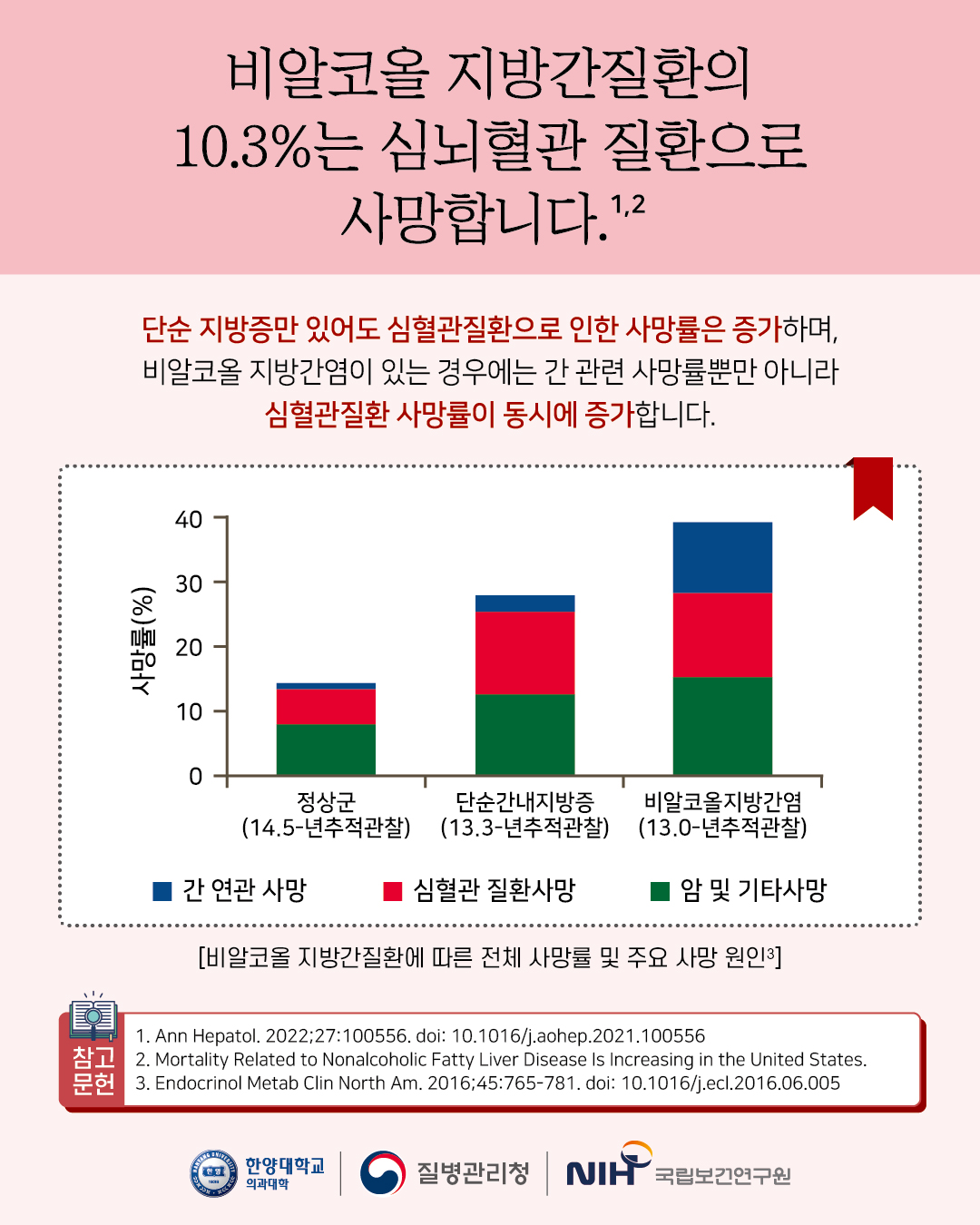

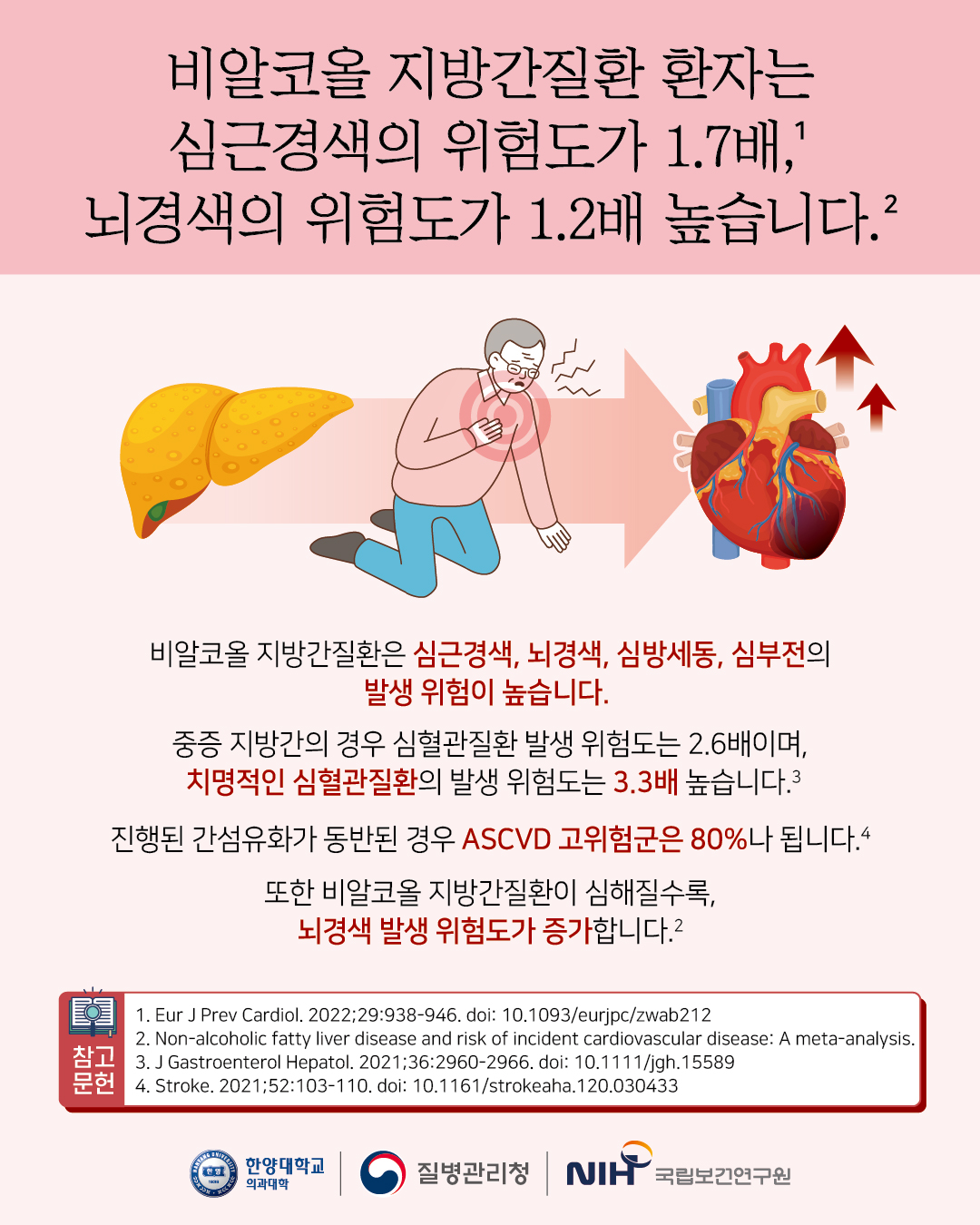


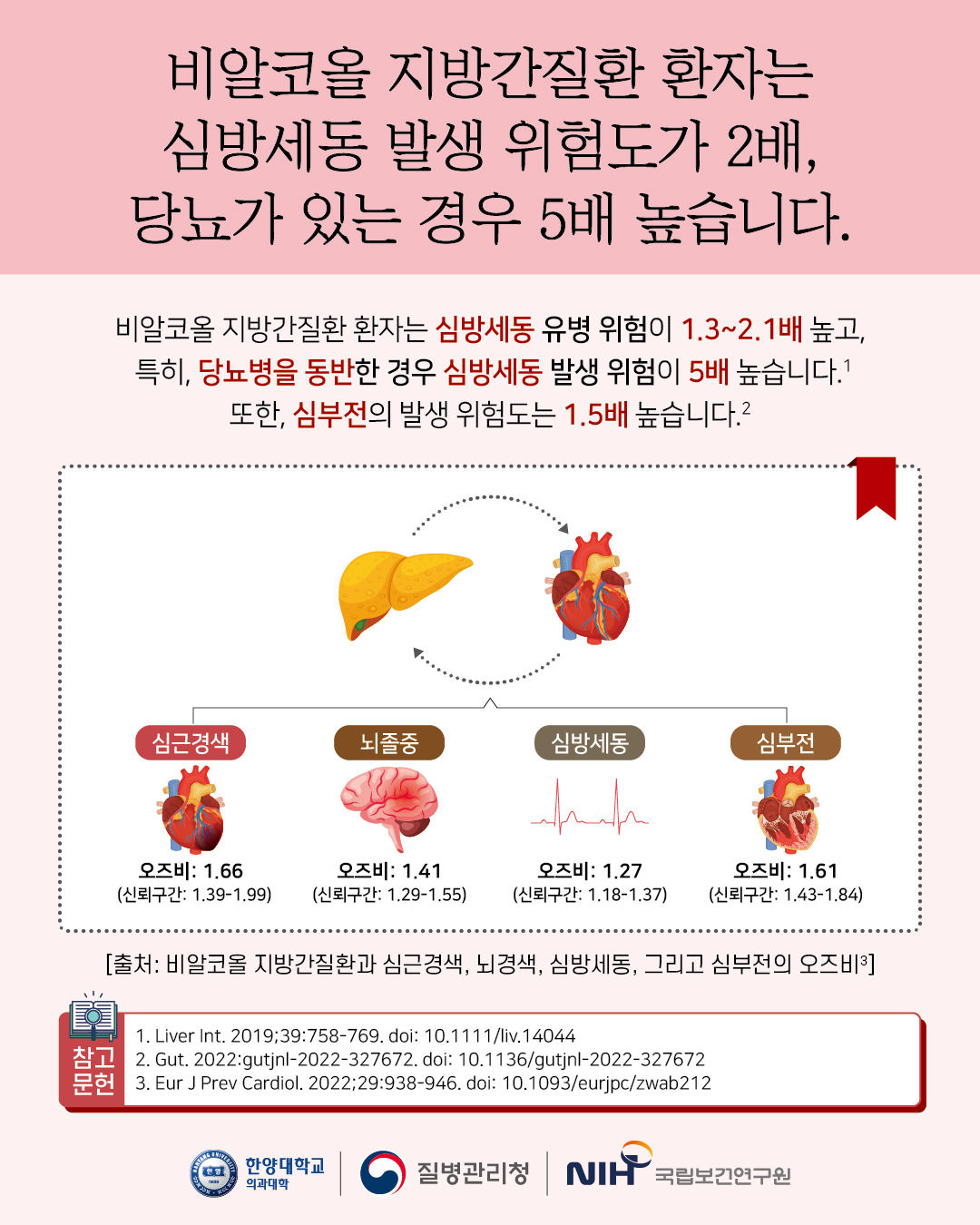


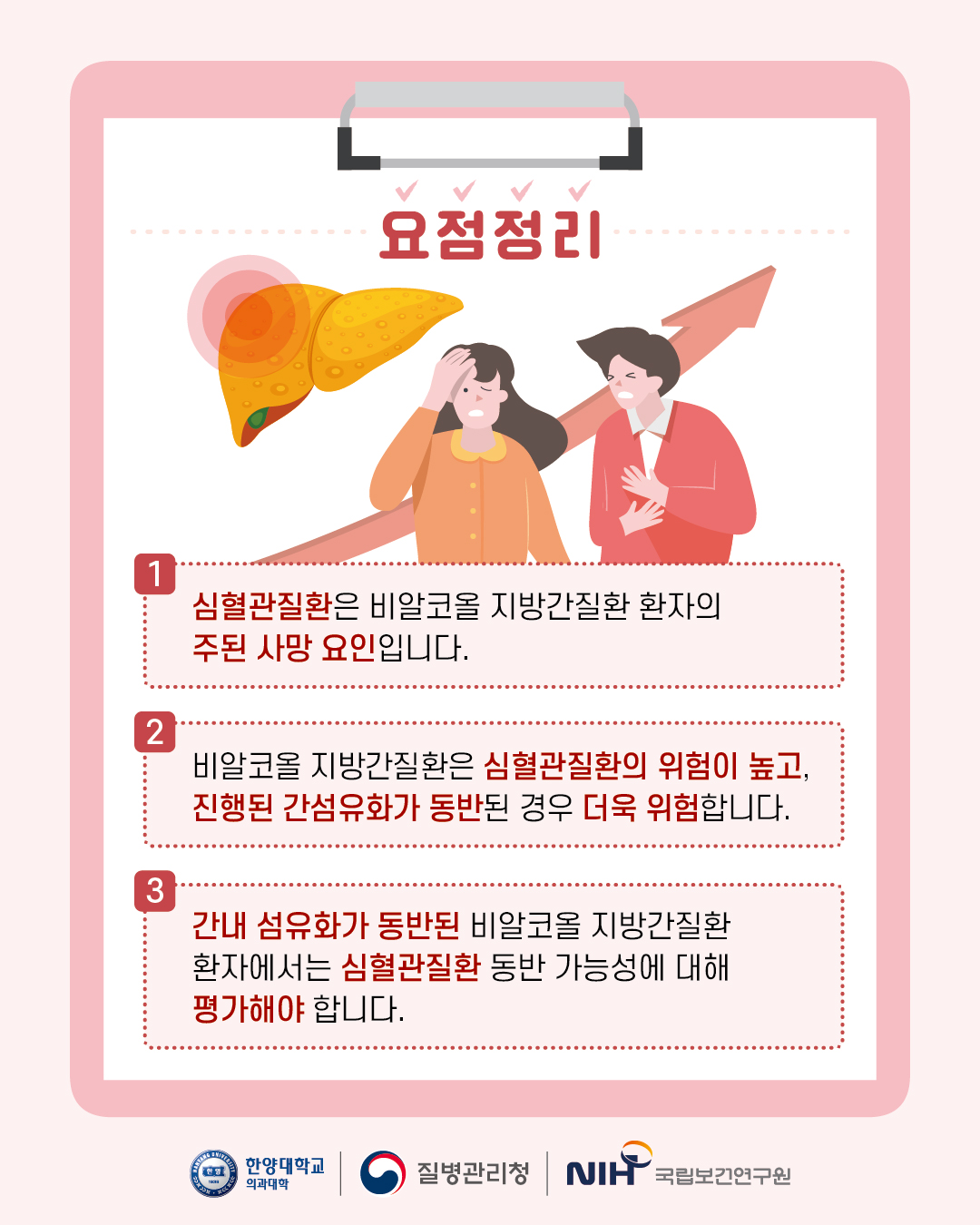


**Fourth week**


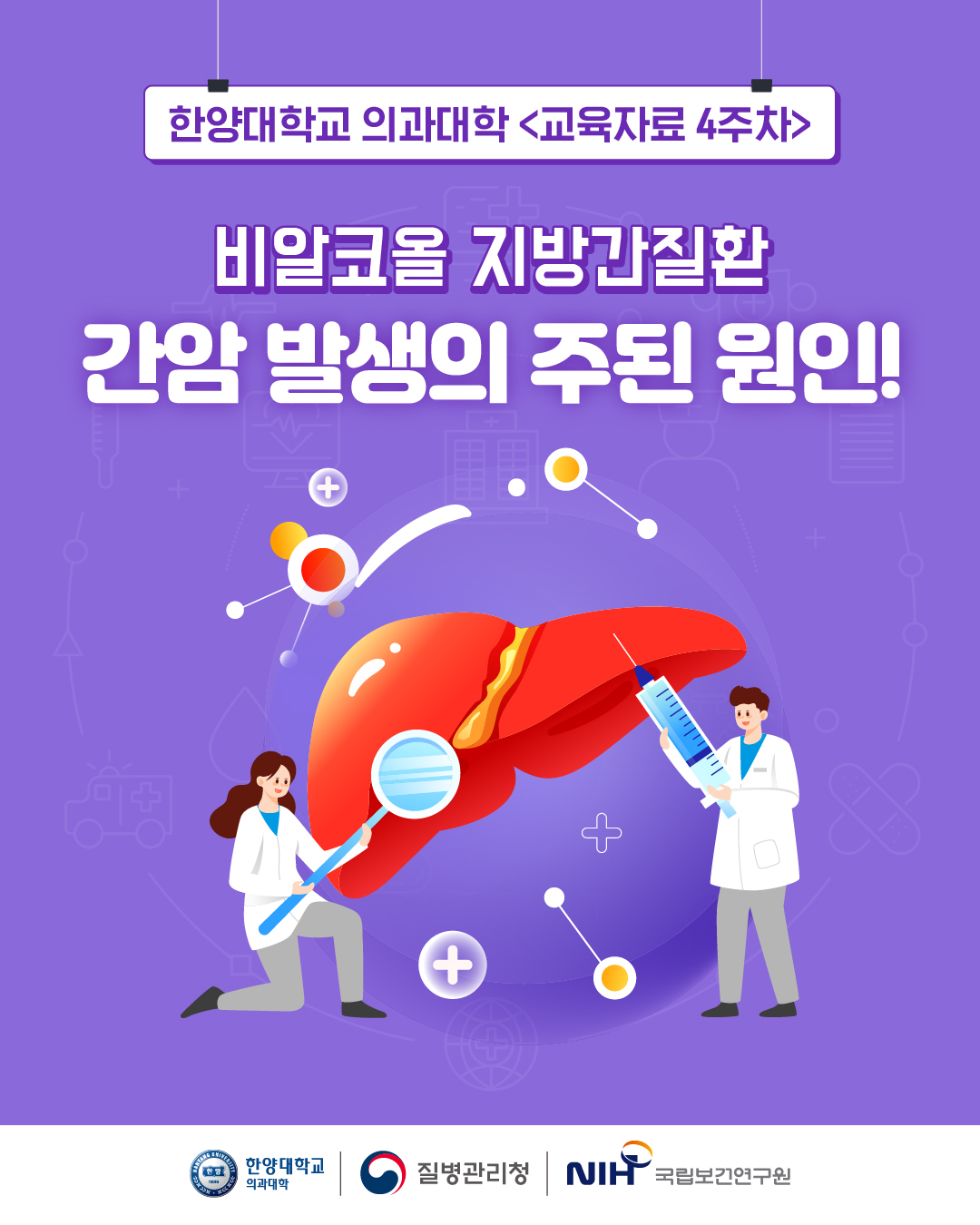


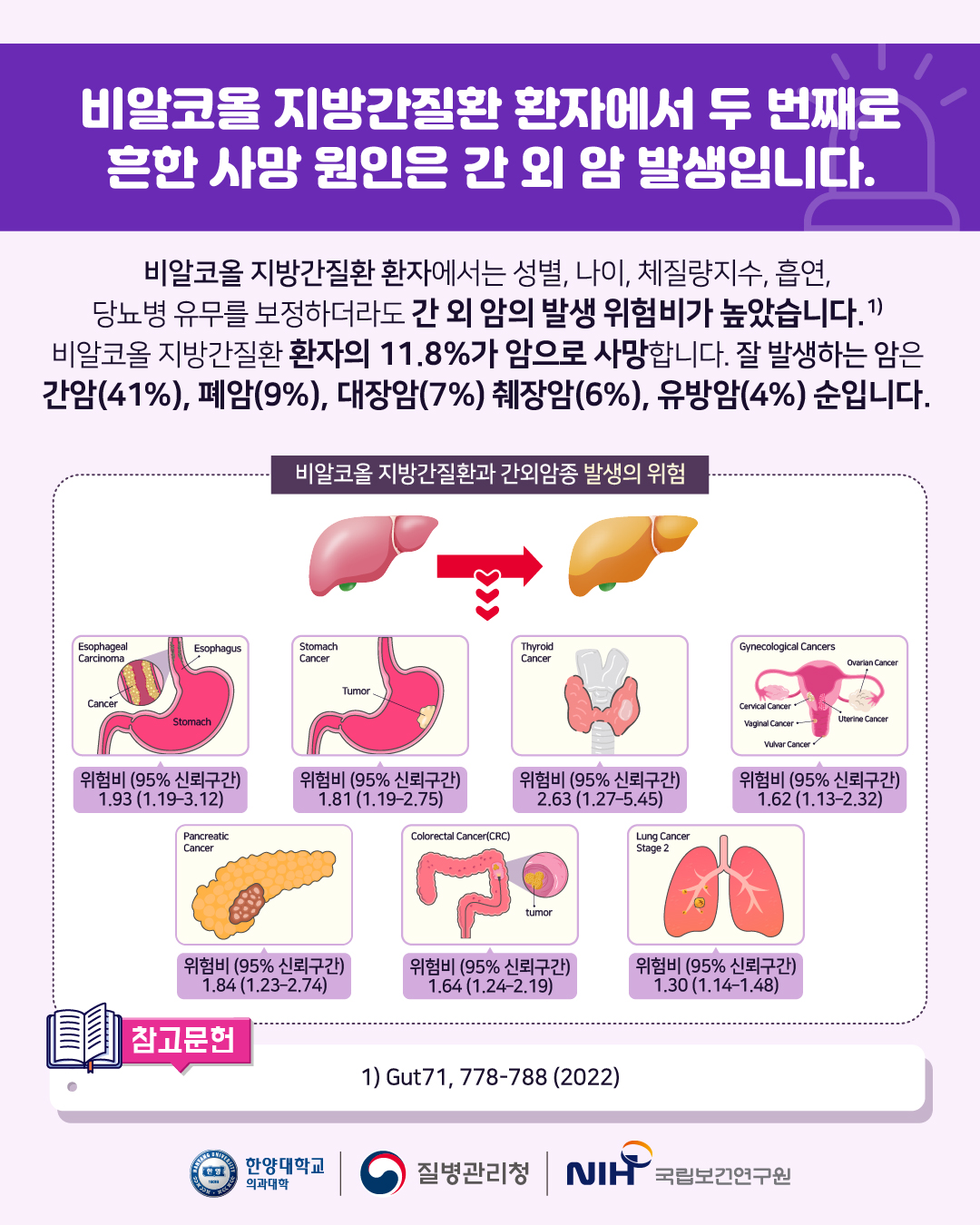


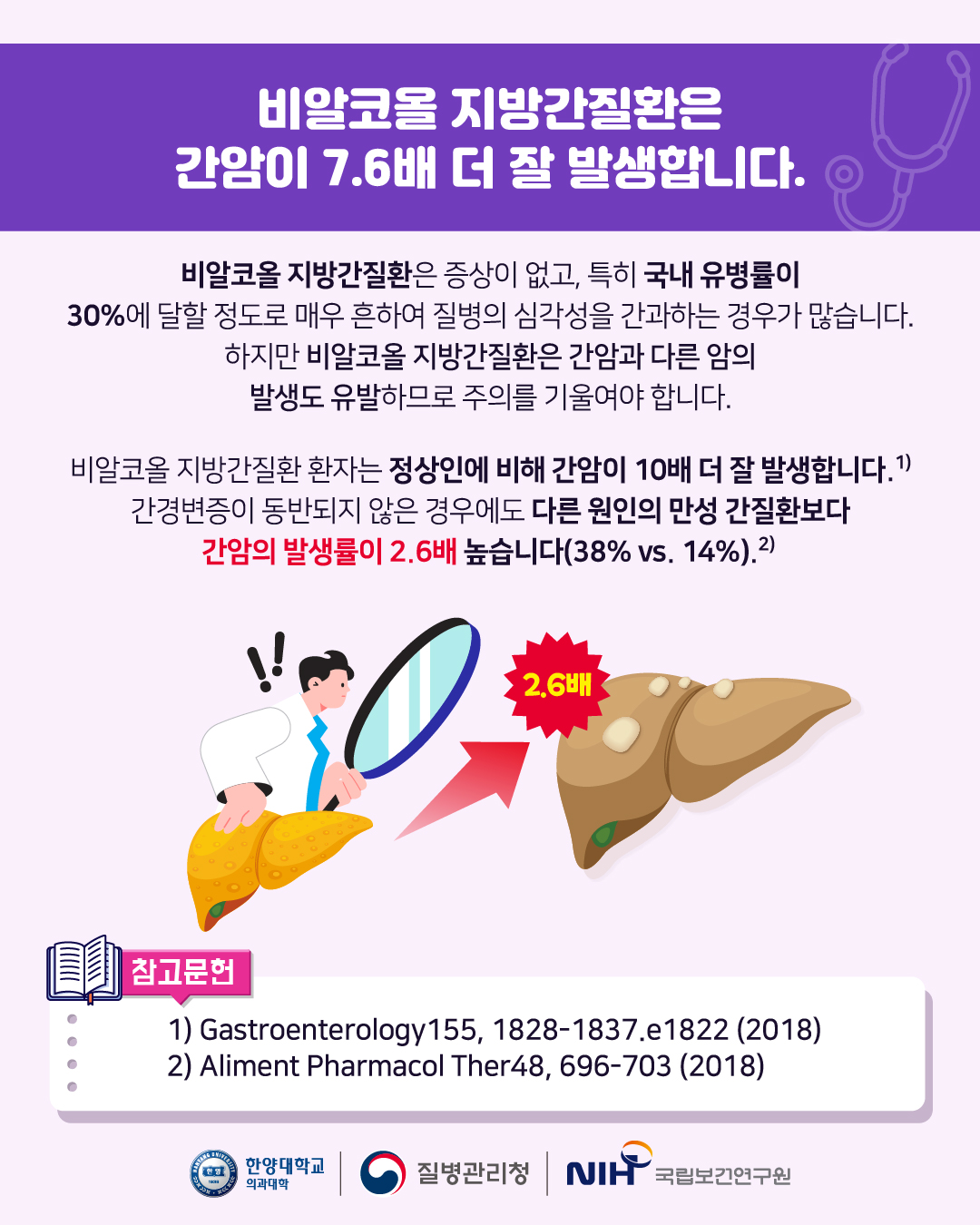


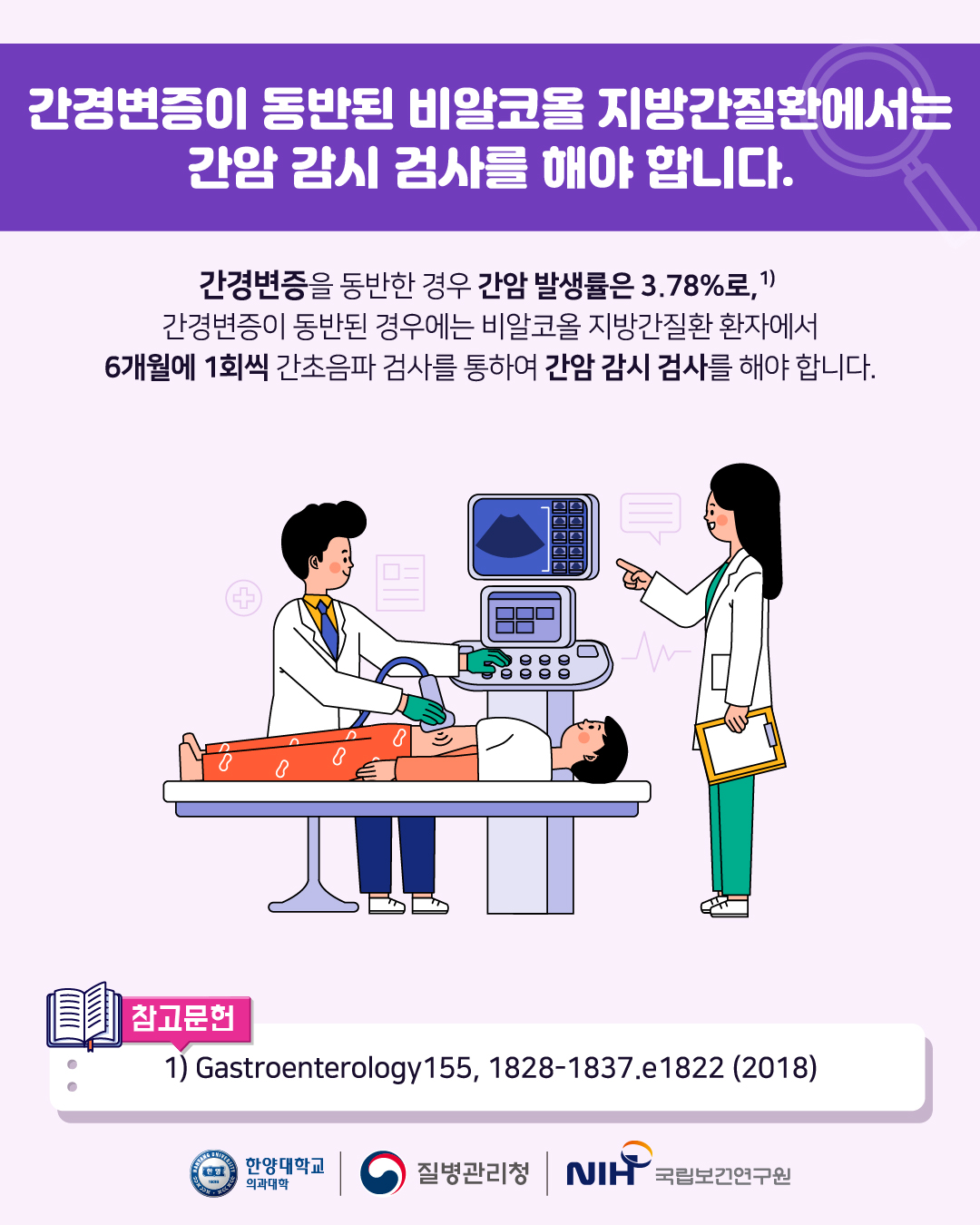


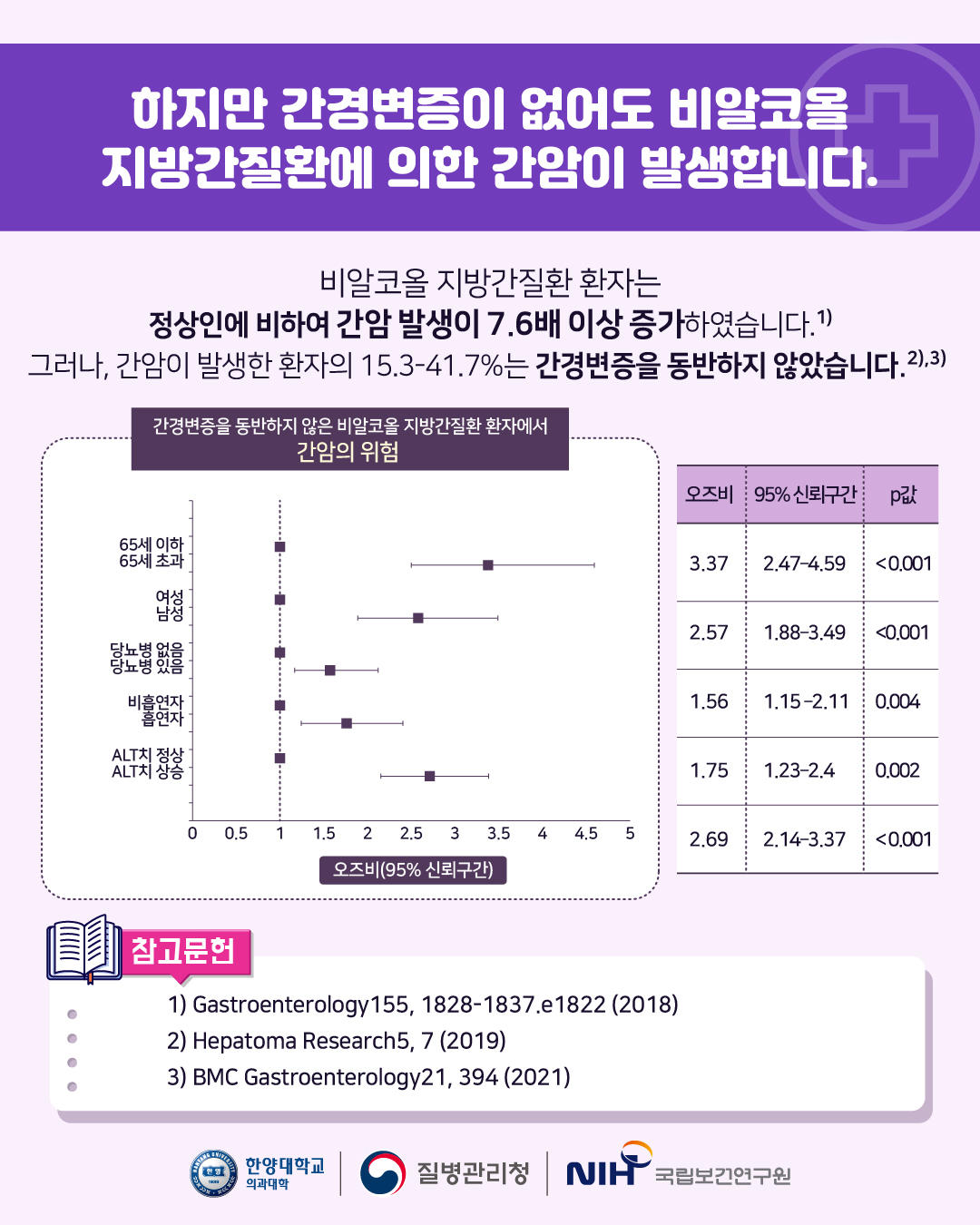


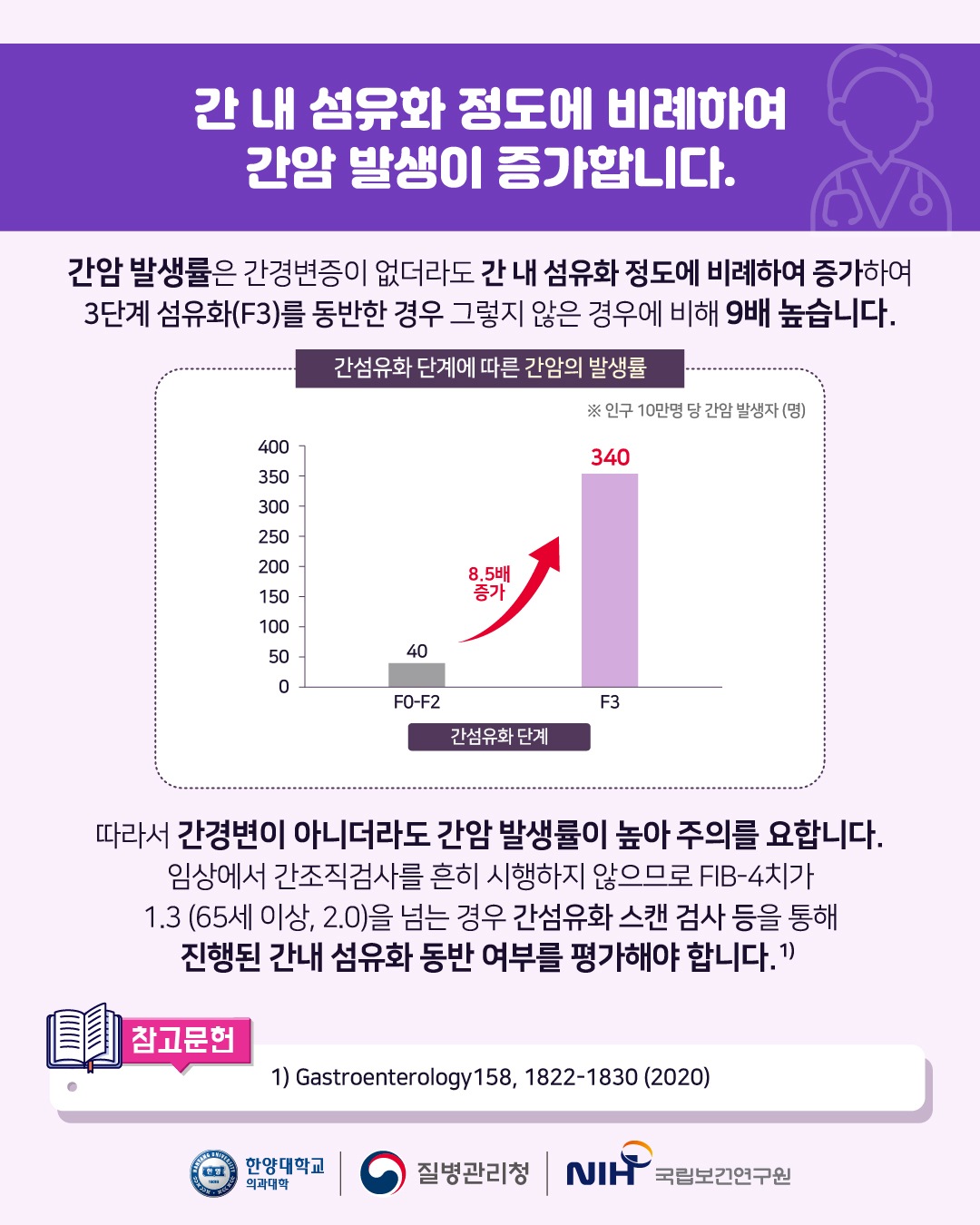


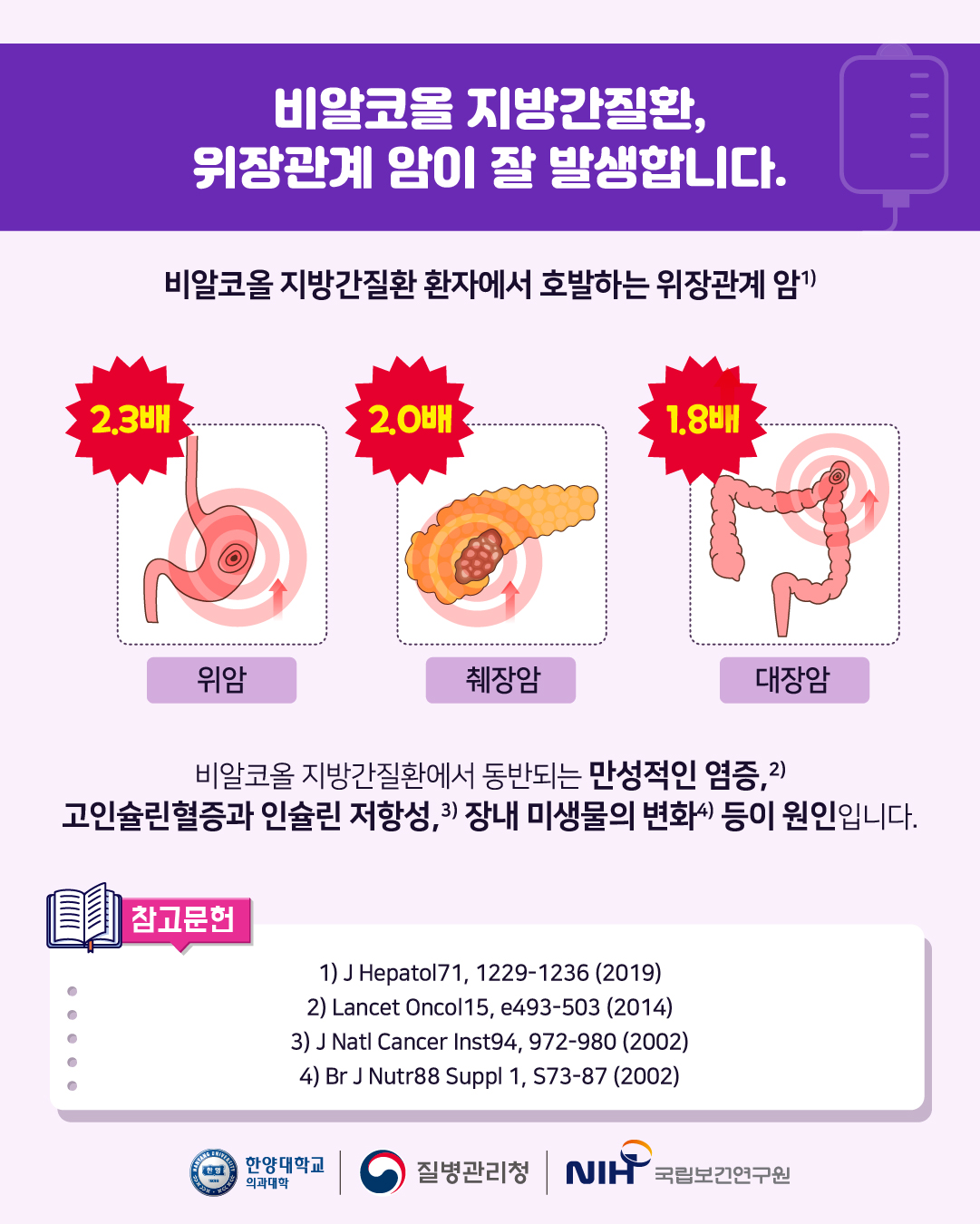


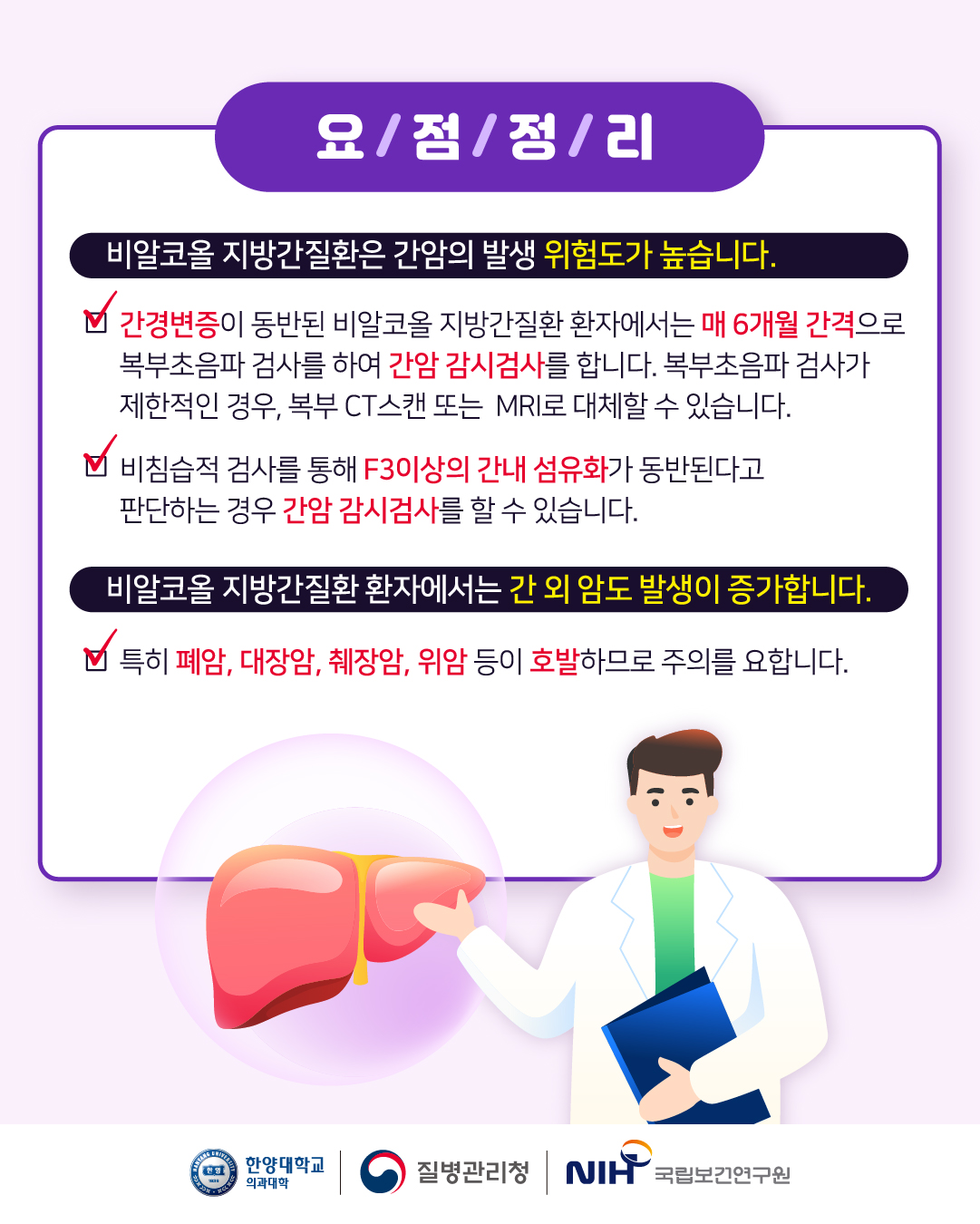


**Fifth week**


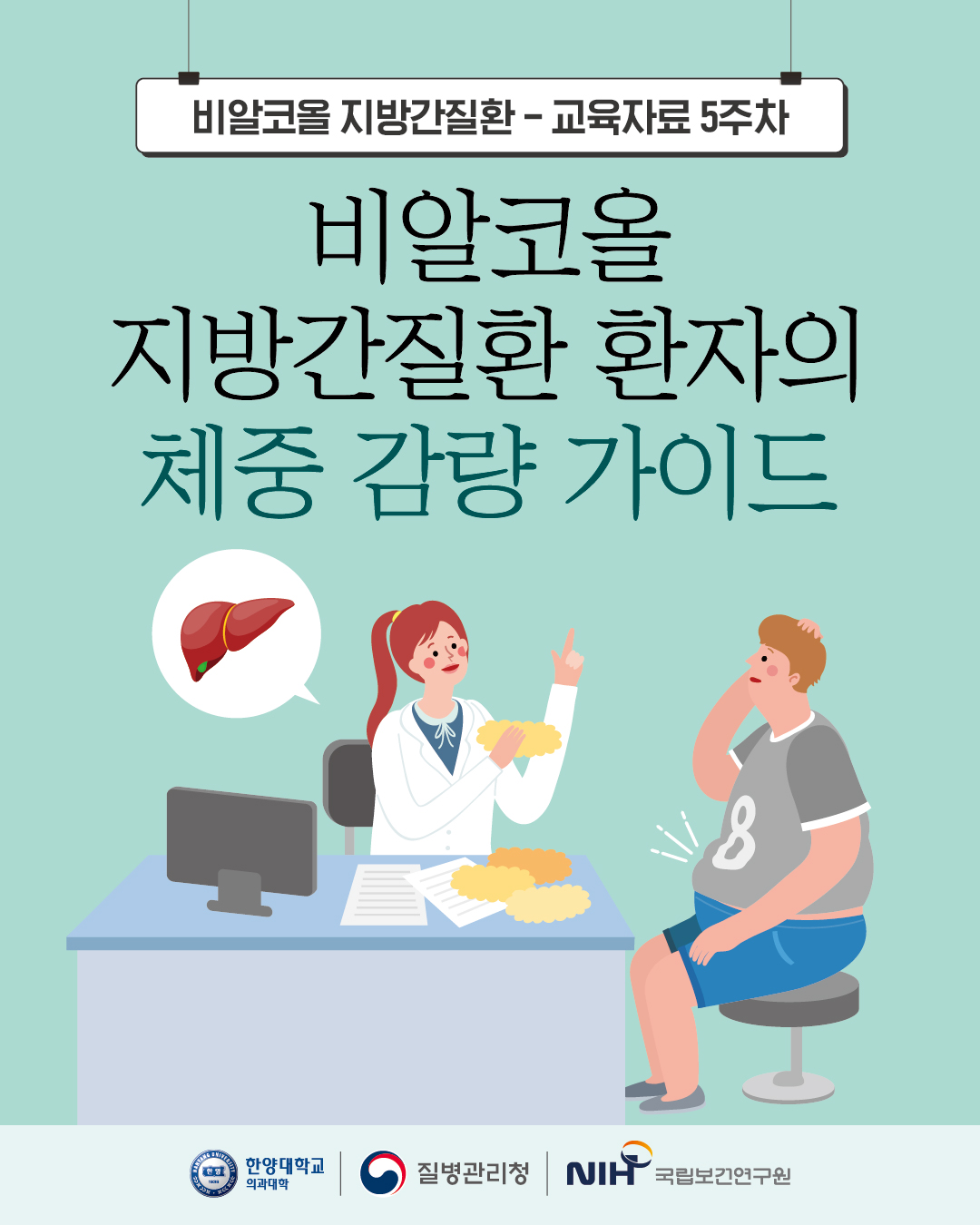


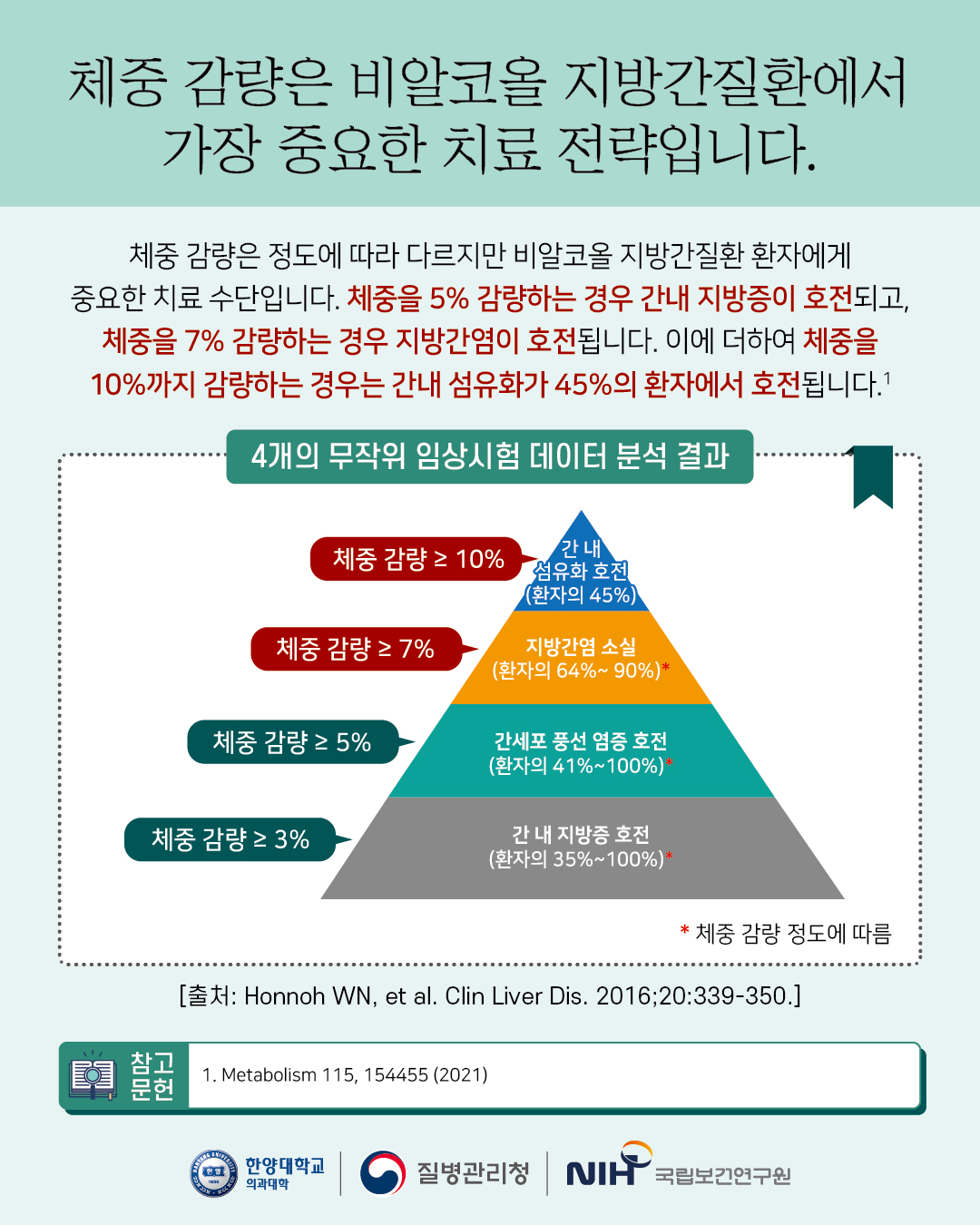


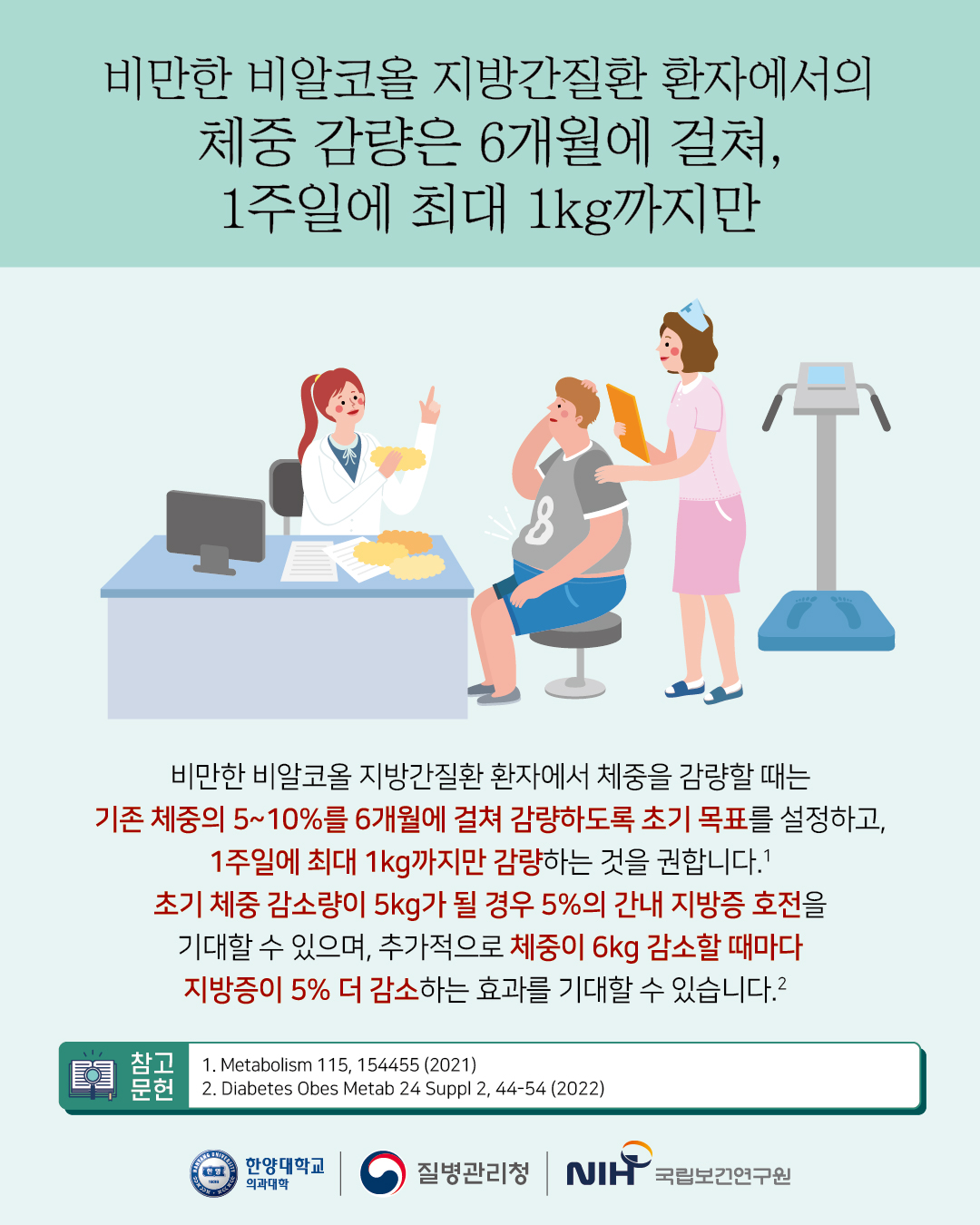


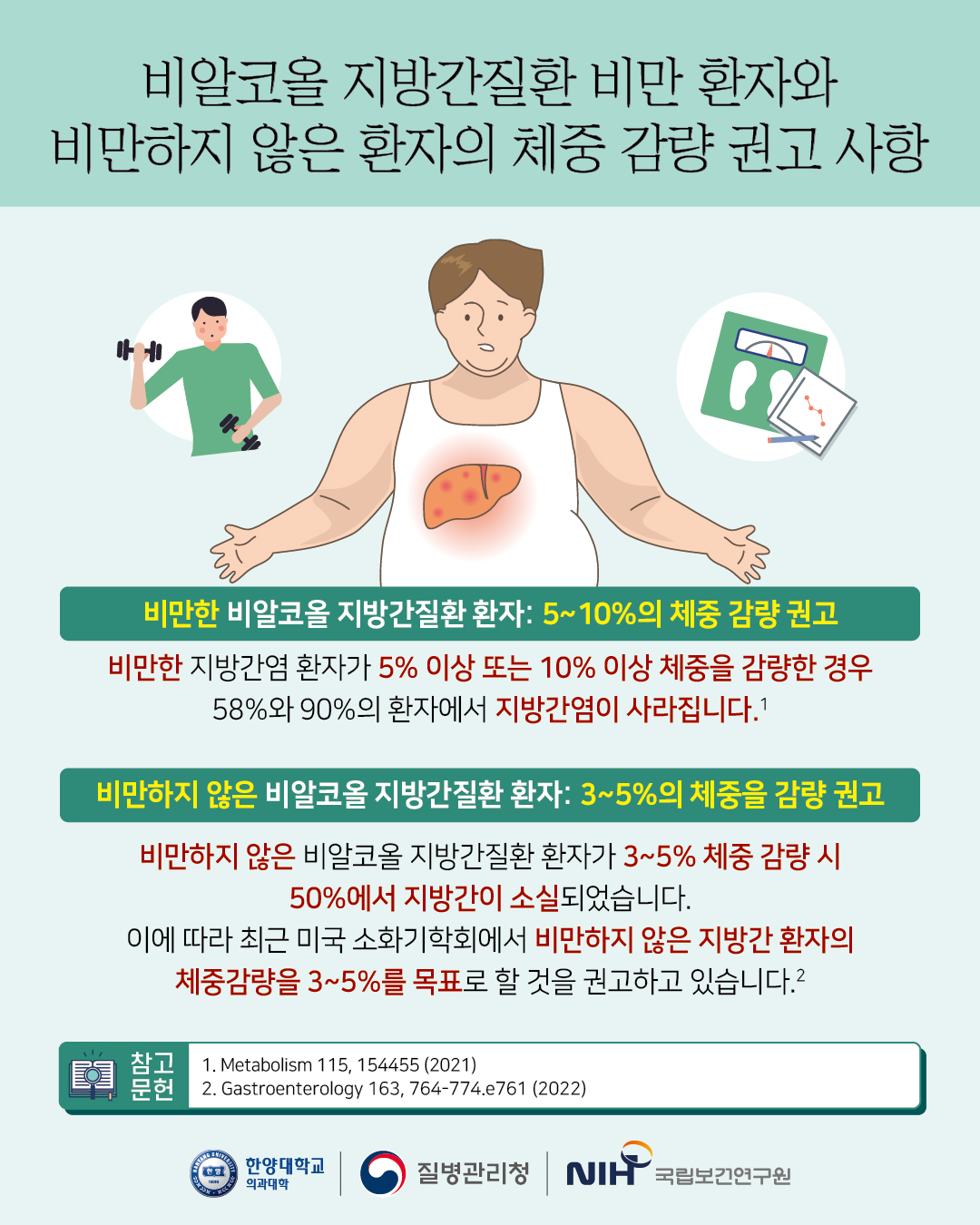


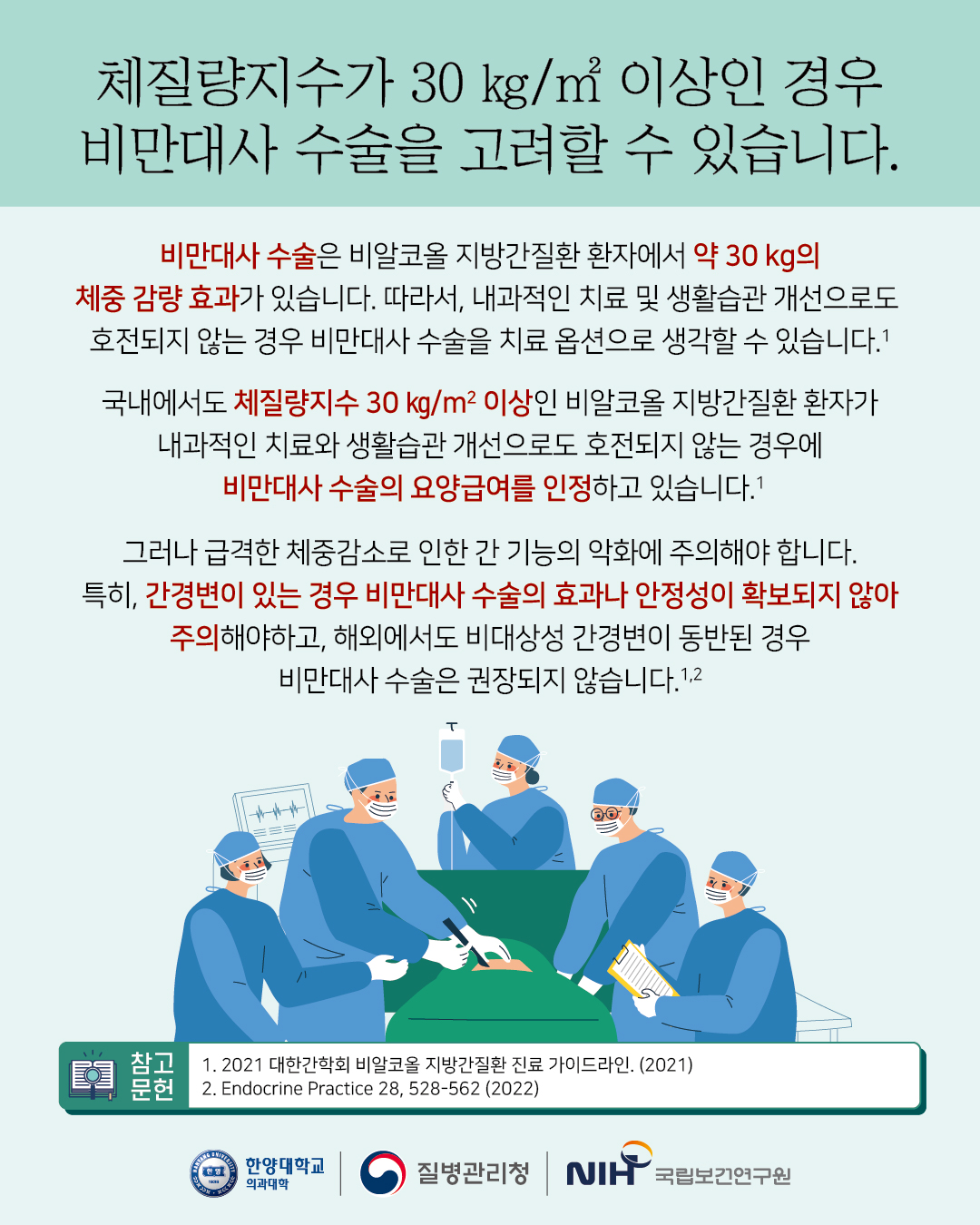


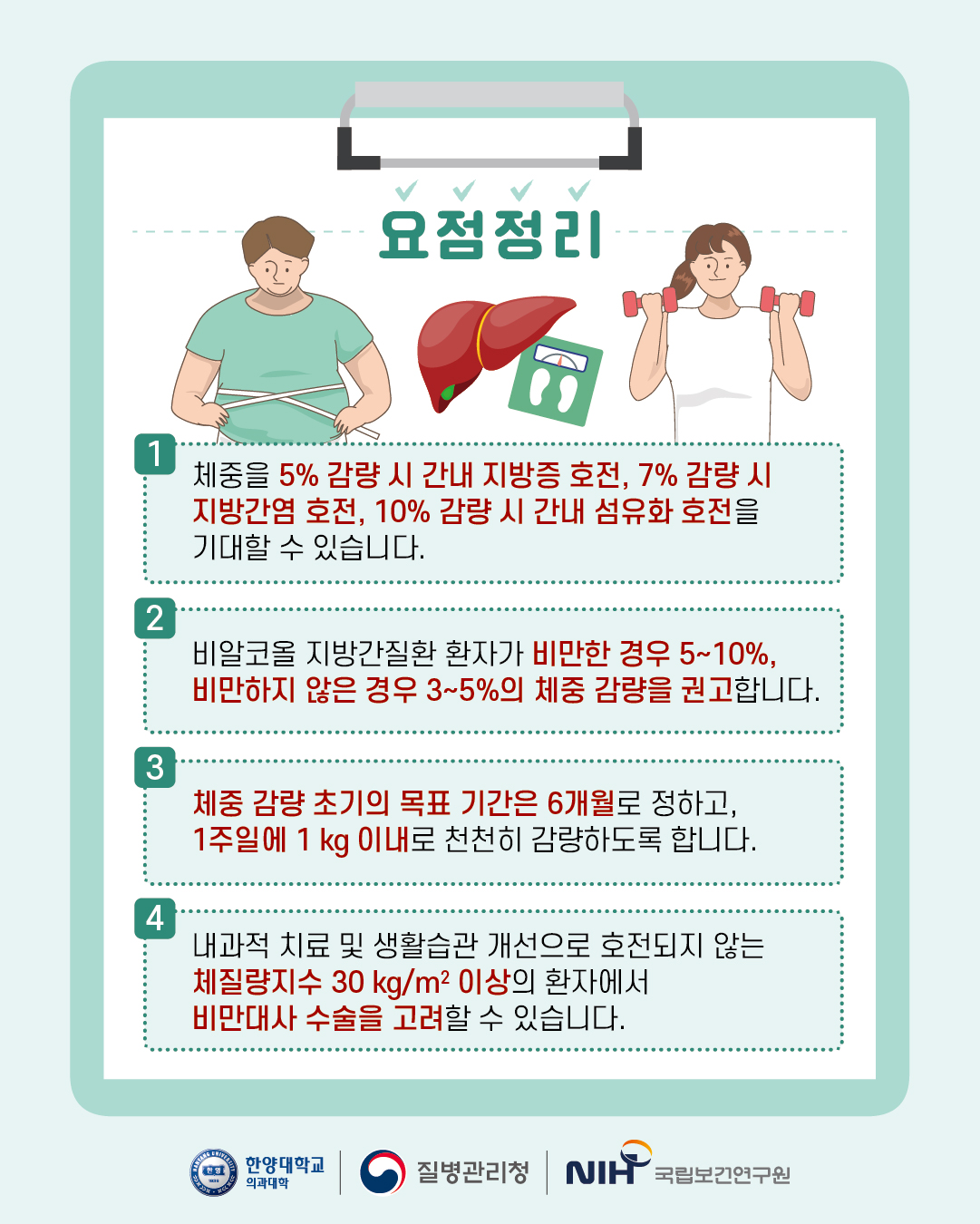


**Sixth week**


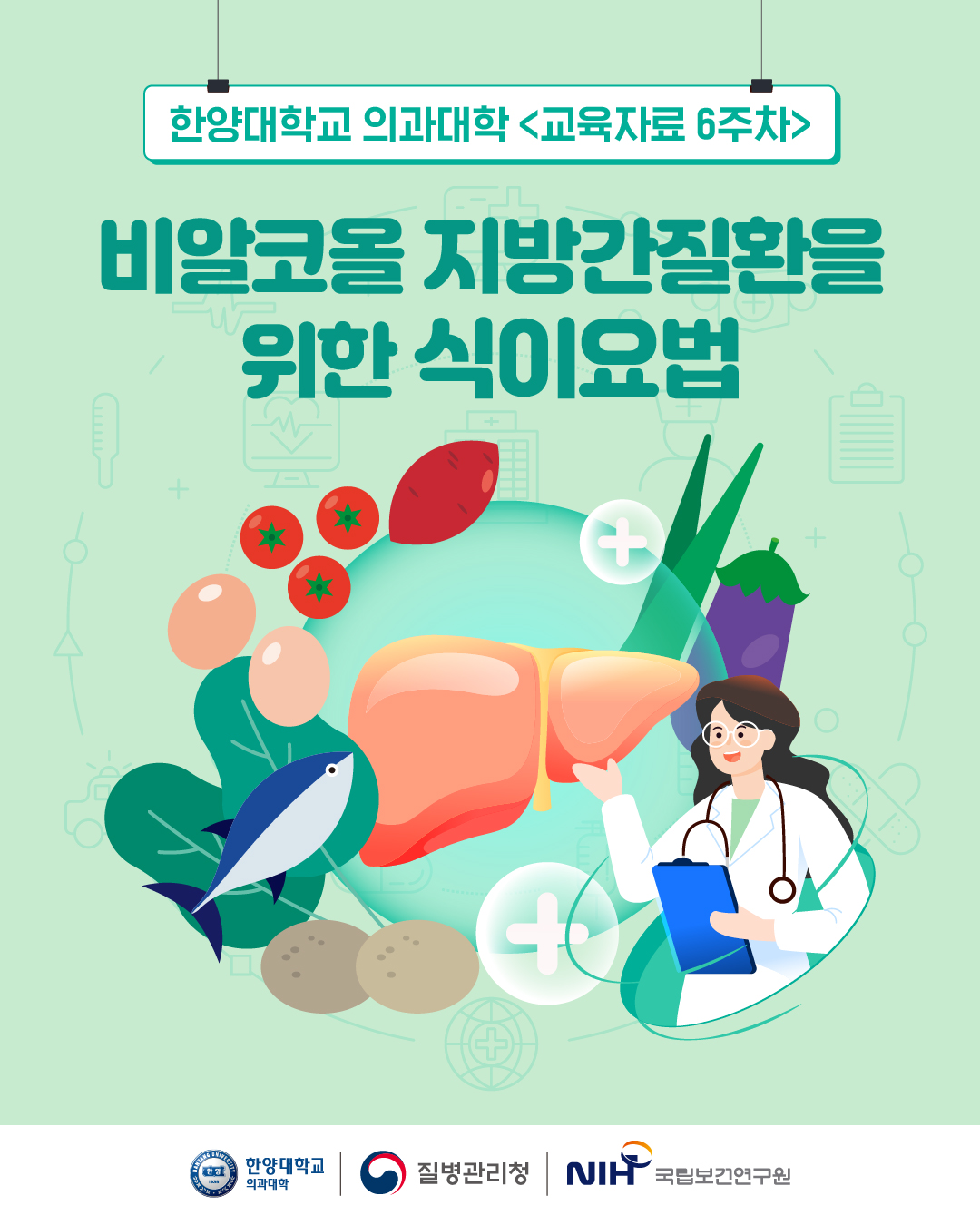


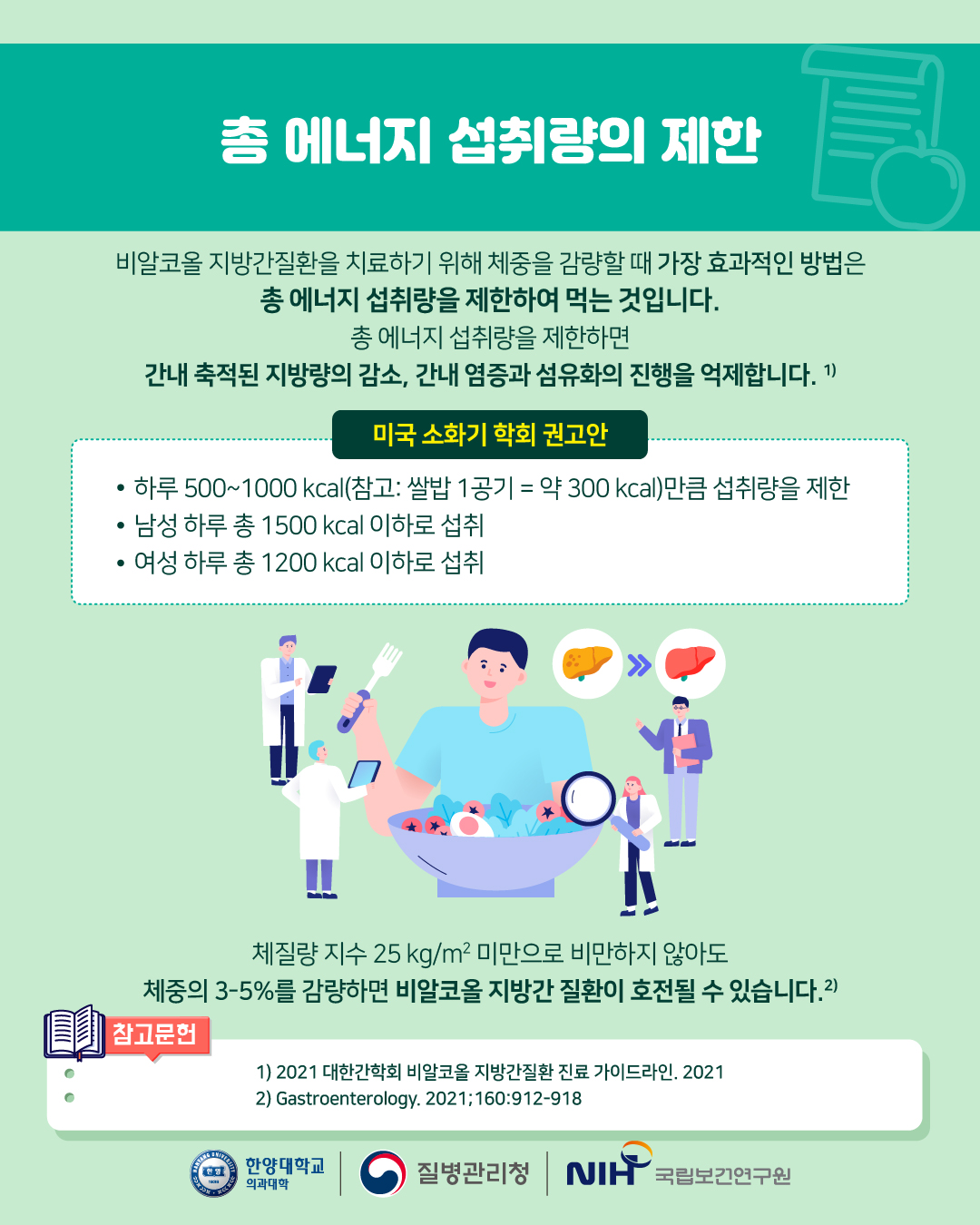


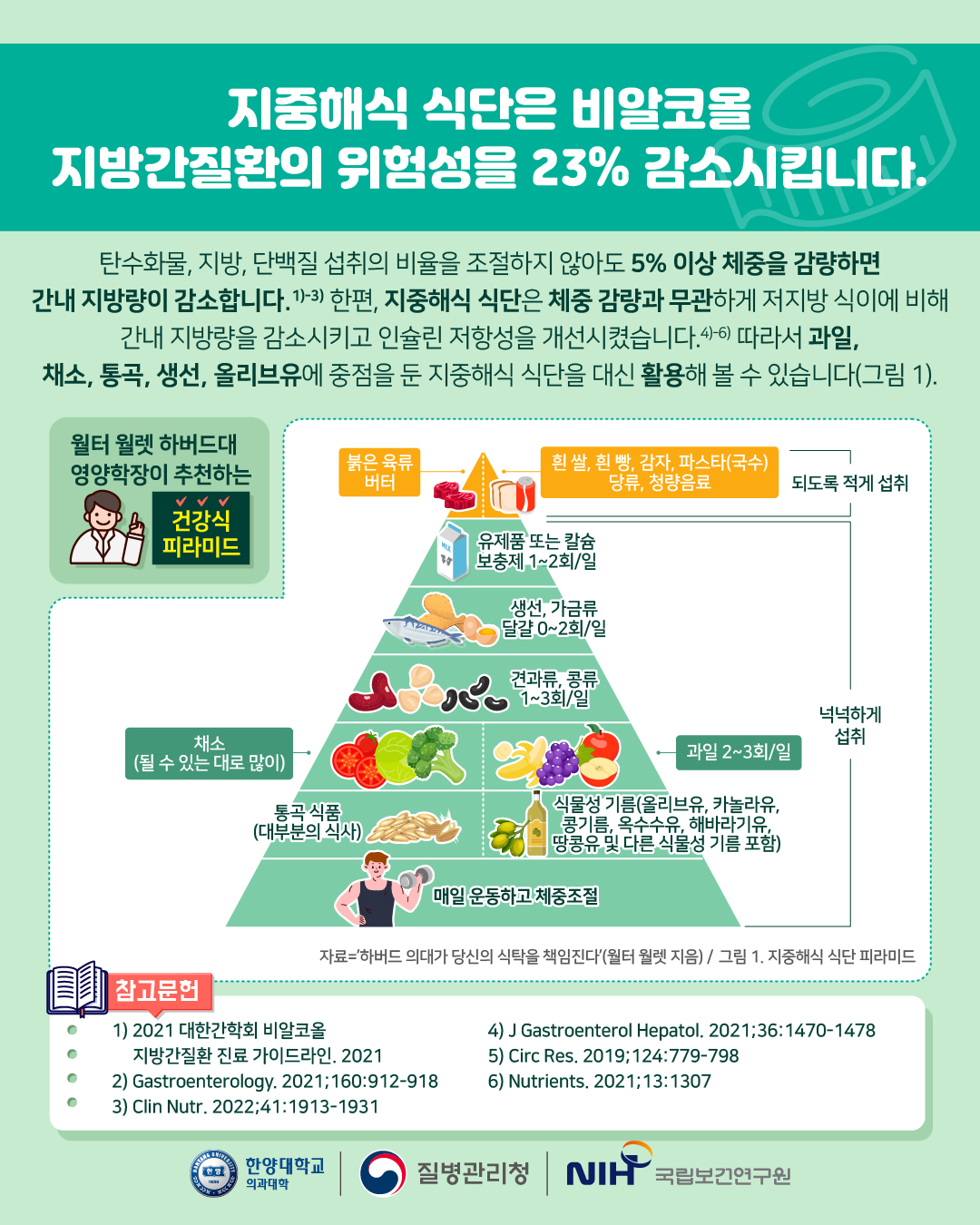


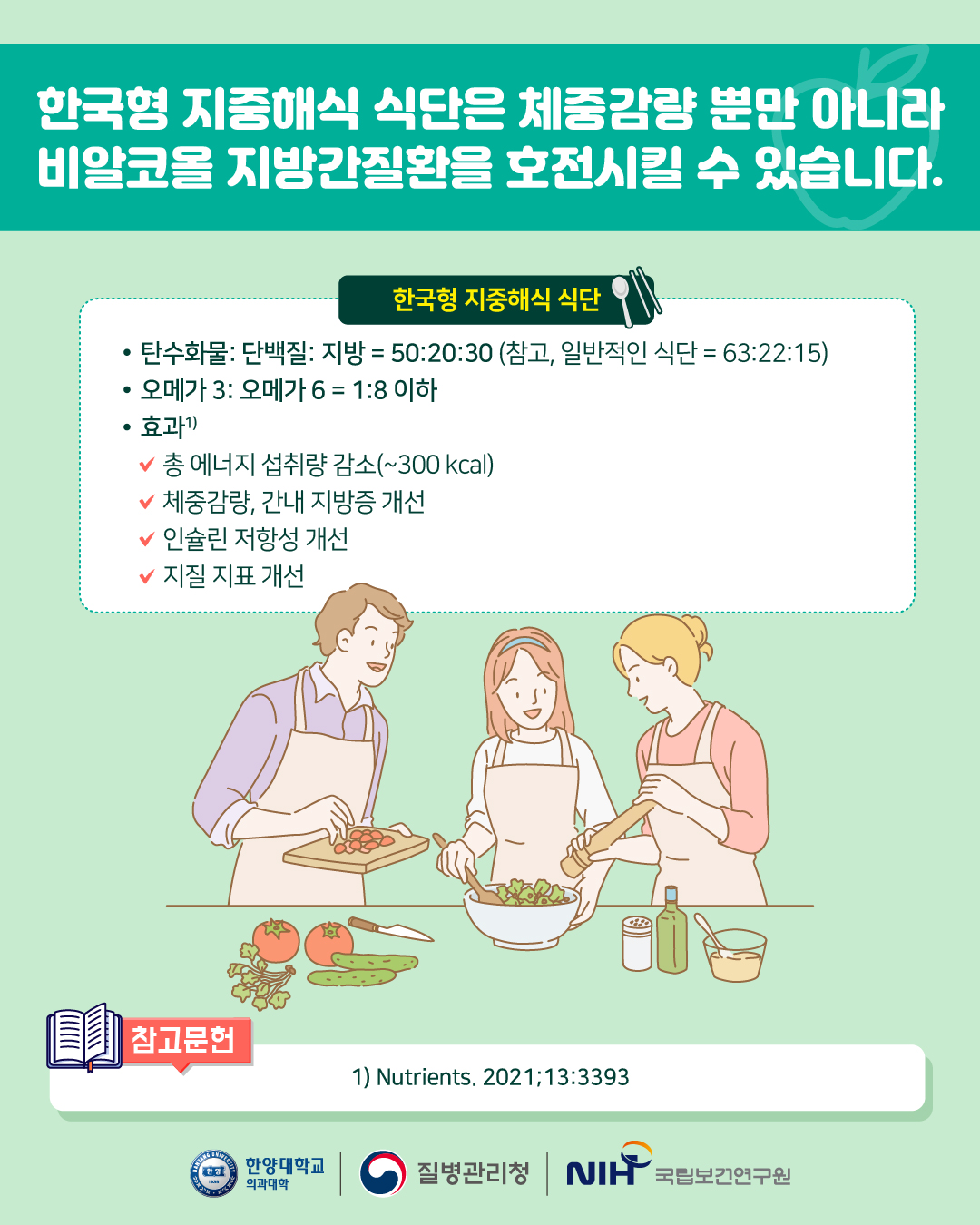


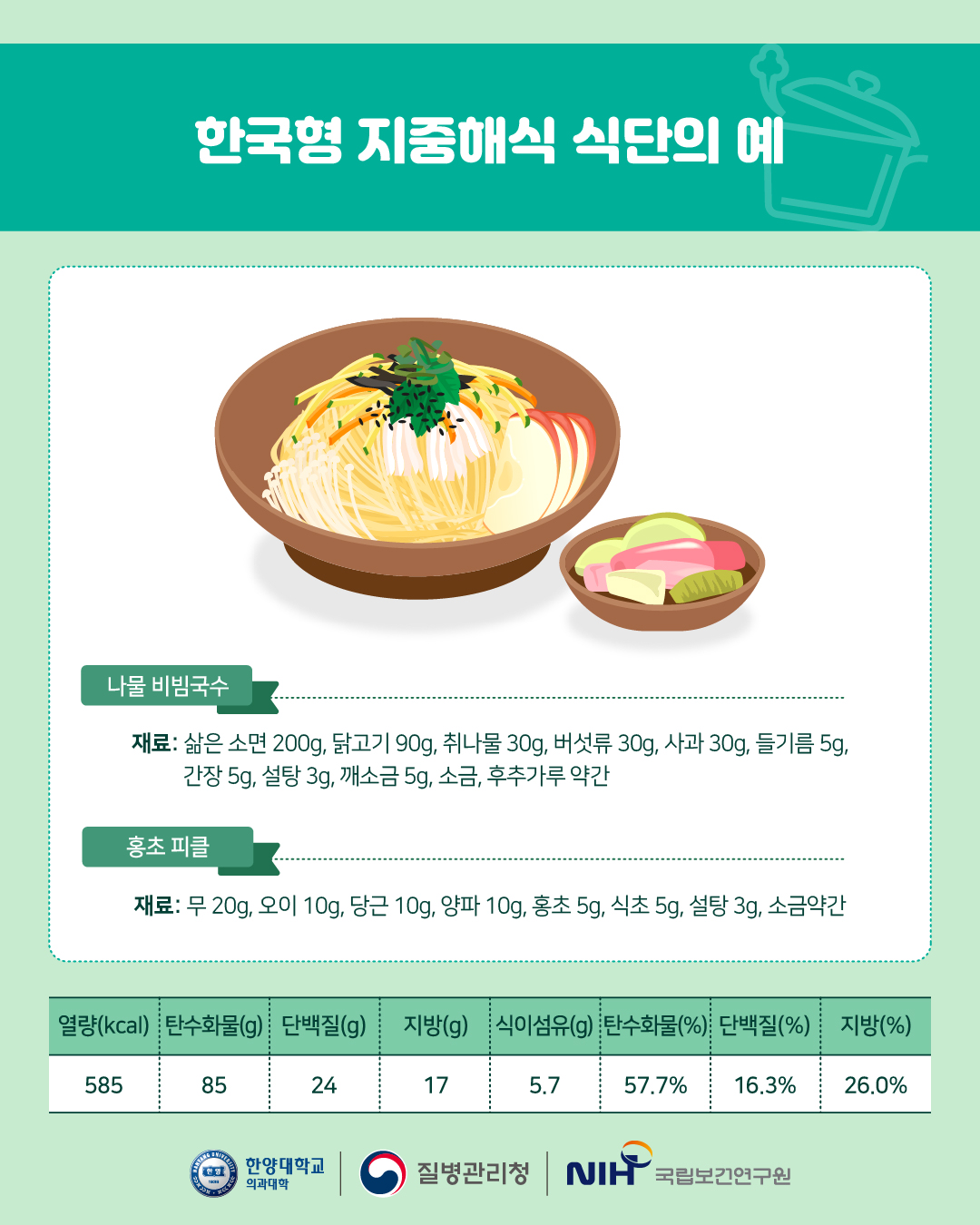


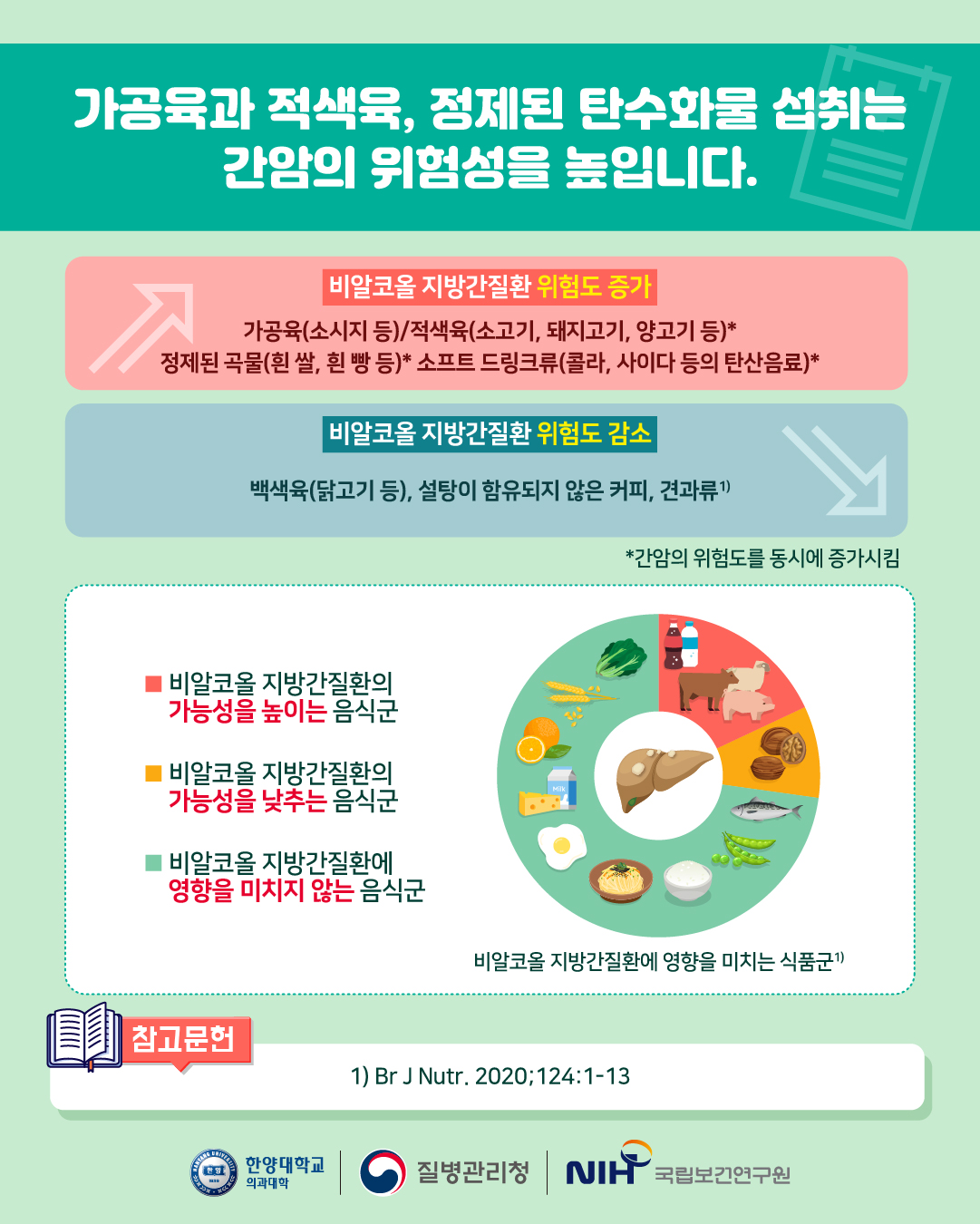


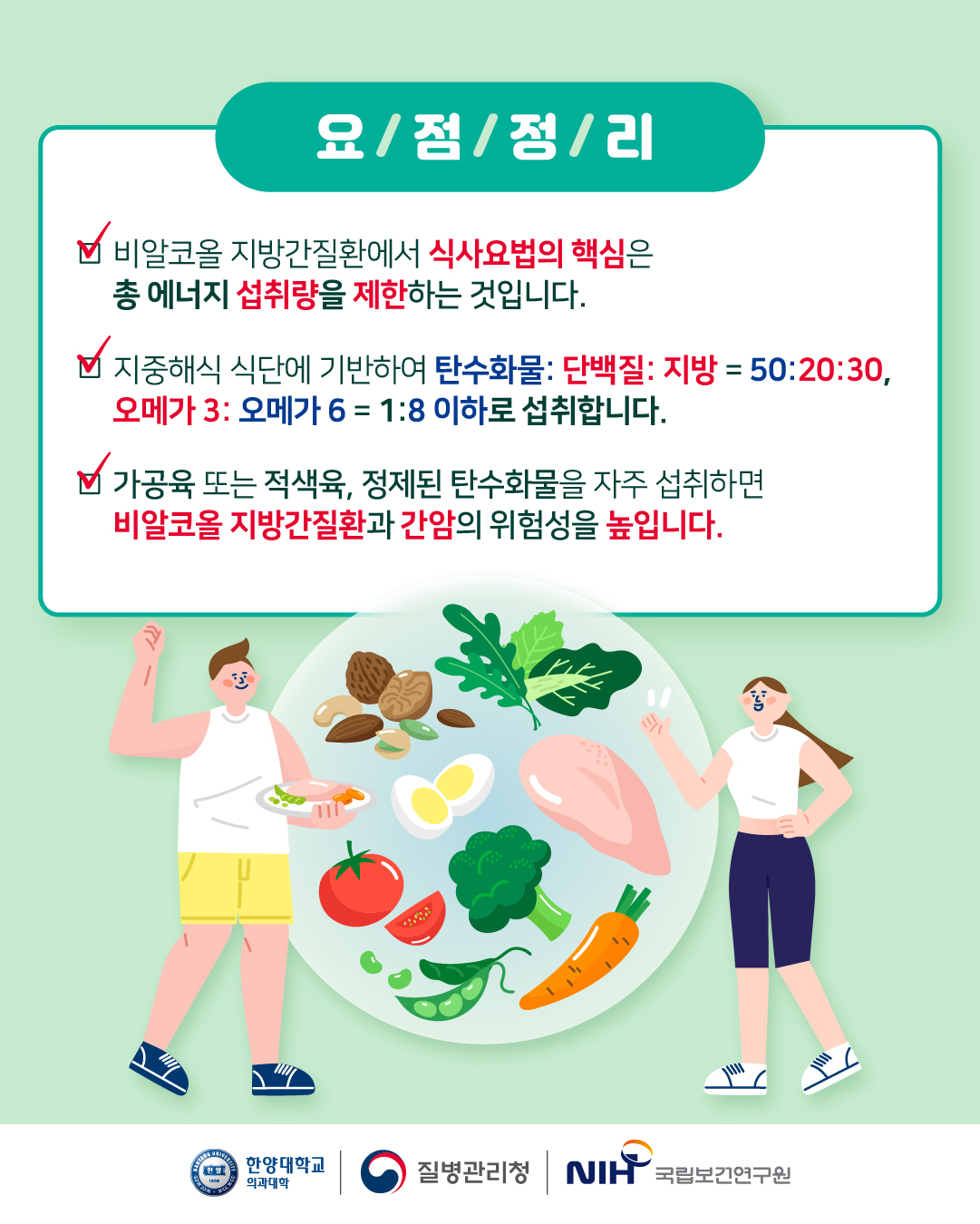


**Seventh week**


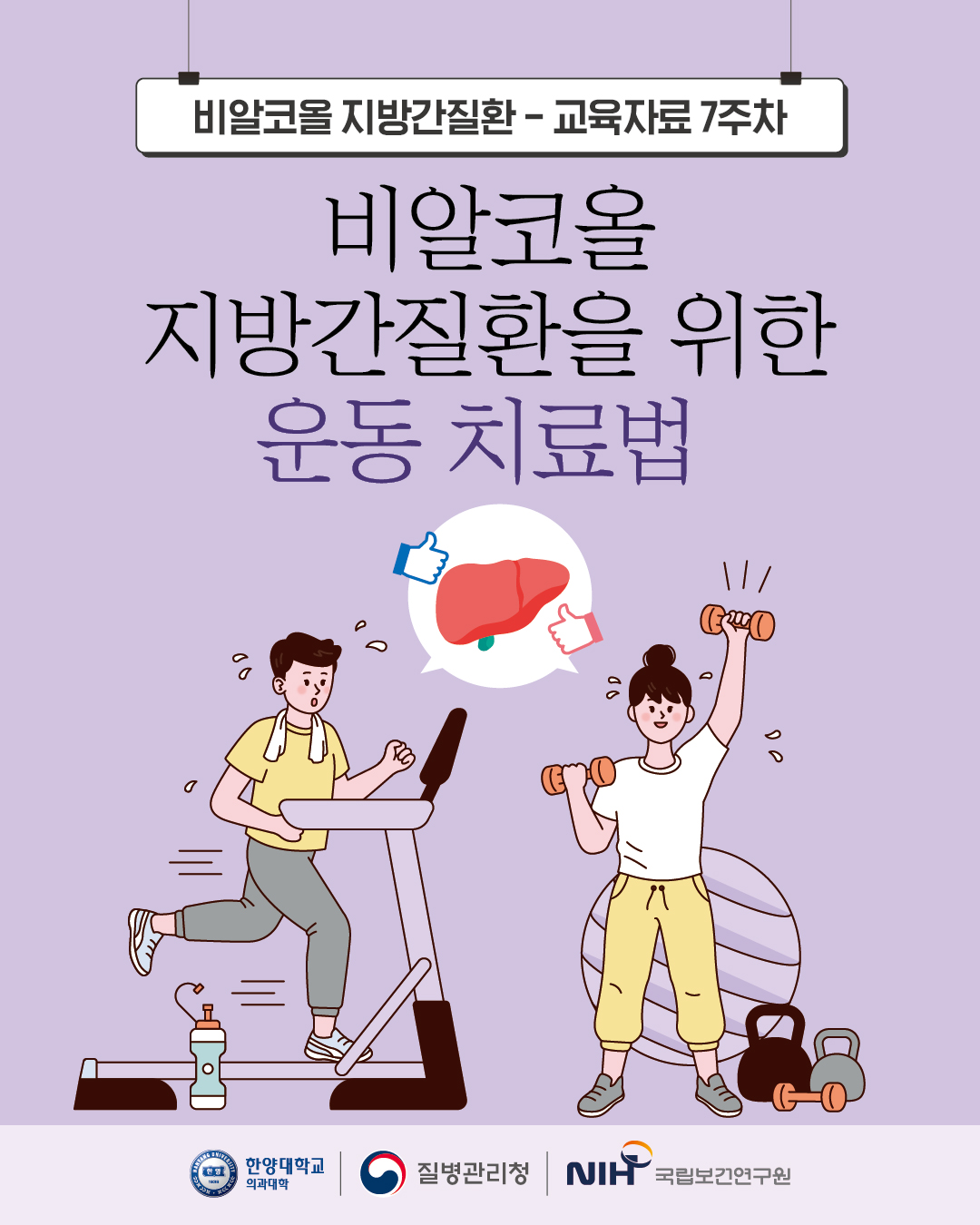


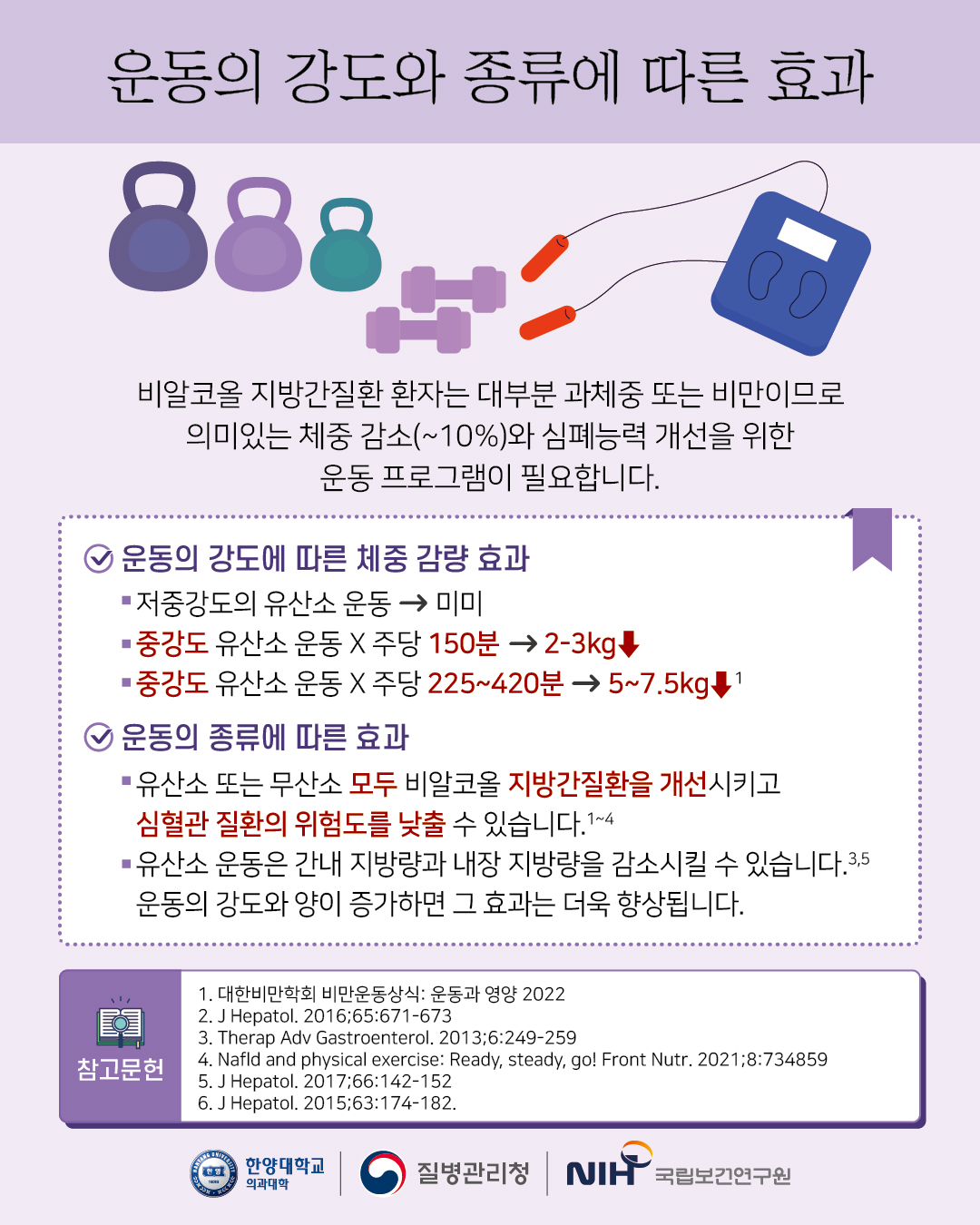


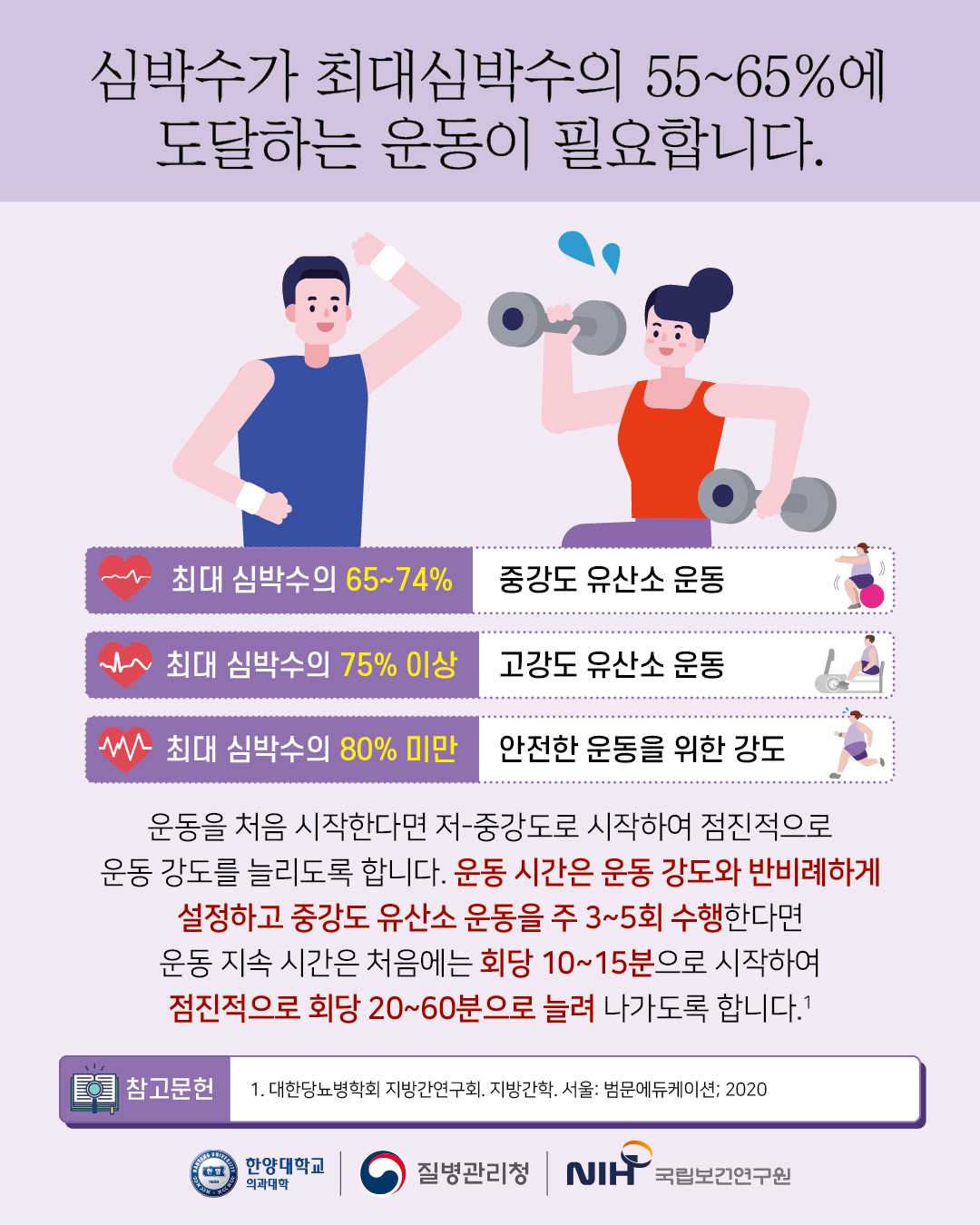


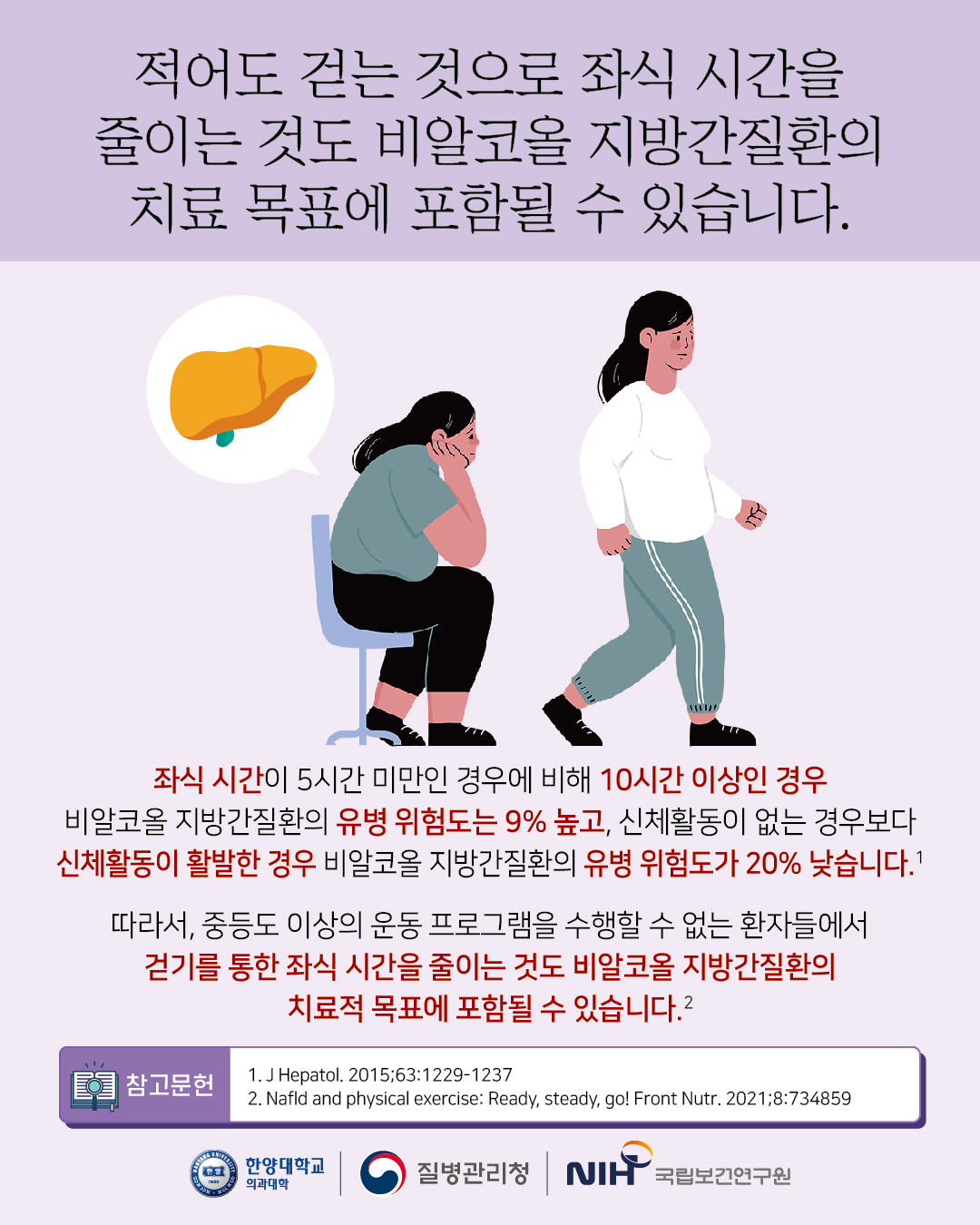

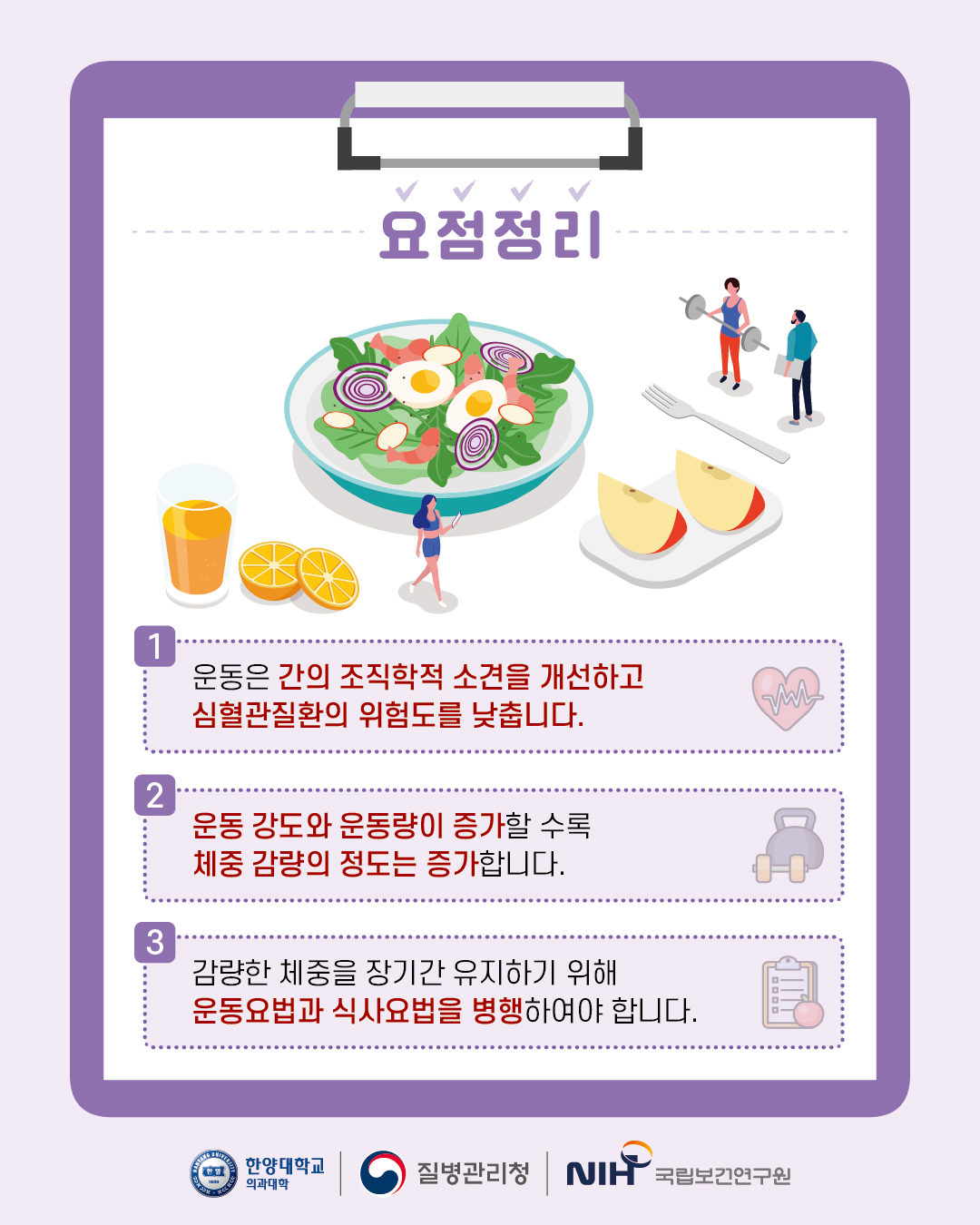

Supplement: Supplementary file 1 [file Data_Sheet_1.docx]
