# Supplementary material for: Barriers to care linkage and educational impact on unnecessary MASLD referrals
Source: Front Med (Lausanne). 2024 Jul 25;11:1407389. doi: 10.3389/fmed.2024.1407389 (PMC11309125; doi:10.3389/fmed.2024.1407389)
Supplement: Supplementary file 2 [file Data_Sheet_2.docx]

**Supplementary Table 1.** Changes in the education of patients with MASLD among PCPs and physicians at referral centers before and after receiving educational materials on MASLD based on positive responses to ' short consultation time’ as a barrier to the Management of MASLD

|  | PCPs  (n = 536) | | | Physicians at referral centers  (n = 55) | | |
| --- | --- | --- | --- | --- | --- | --- |
|  | Initial survey | Follow-up survey | *P** | Initial survey | Follow-up survey | *P** |
| **Management** |  |  |  |  |  |  |
| Briefly mention that the patient has MASLD and do not proceed with any additional measures | 91 (17.0%) | 106 (19.8%) | 0.211 | 13 (26.6%) | 4 (7.3%) | 0.016 |
| Refer patient to the gastroenterology or a tertiary hospital | 171 (31.9%) | 178 (33.2%) | 0.600 | 16 (29.1%) | 23 (41.8%) | 0.146 |
| Take no additional measure if patient has normal AST and ALT levels. | 108 (20.1%) | 94 (17.5%) | 0.251 | 13 (23.6%) | 14 (25.5%) | 1.000 |
| Mention that the patient has fatty liver and recommend lifestyle modification | 322 (60.1%) | 319 (59.5%) | 0.880 | 35 (63.6%) | 36 (65.5%) | 1.000 |
| Order additional tests (blood glucose test, lipid panel, etc.) for metabolic syndrome (diabetes, dyslipidemia, etc.) | 256 (47.8%) | 267 (49.8%) | 0.434 | 29 (52.7%) | 32 (58.2%) | 0.663 |
| Order additional tests regarding liver fibrosis | 47 (8.8%) | 67 (12.5%) | 0.023 | 7 (12.7%) | 18 (32.7%) | 0.022 |
| Order additional tests to determine whether patient also has CVD | 19 (3.5%) | 44 (8.2%) | <0.001 | 2 (3.6%) | 3 (5.5%) | 1.000 |
| **Education** |  |  |  |  |  |  |
| Provide no further explanation | 30 (5.6%) | 41 (7.6%) | 0.193 | 3 (5.5%) | 6 (10.9%) | 0.371 |
| Explain the increased risk of disease progression into cirrhosis or liver cancer | 363 (67.7%) | 378 (70.5%) | 0.273 | 38 (69.1%) | 44 (80.0%) | 0.211 |
| Explain the increased risk of metabolic diseases (diabetes, dyslipidemia, etc.) | 424 (79.1%) | 413 (77.1%) | 0.386 | 45 (81.8%) | 43 (78.2%) | 0.803 |
| Explain the increased risk of CVD | 116 (21.6%) | 128 (23.9%) | 0.342 | 13 (23.6%) | 20 (36.4%) | 0.169 |
| Explain the increased risk of ischemic stroke | 77 (14.4%) | 90 (16.8%) | 0.228 | 8 (14.5%) | 14 (25.5%) | 0.114 |
| Explain the increased risk of extrahepatic cancer | 72 (13.4%) | 75 (14.0%) | 0.836 | 5 (9.1%) | 9 (16.4%) | 0.343 |

* *P* value was derived from McNemar test.

Abbreviations: MASLD, metabolic dysfunction-associated steatotic liver disease; PCP, primary care physician; CVD, cardiovascular disease.

**Supplementary Table 2.** Changes in the education of patients with MASLD among PCPs and physicians at referral centers before and after receiving educational materials on MASLD based on positive responses to 'lack of adequate educational materials' as a barrier to the management of MASLD

|  | PCPs  (n = 519) | | | Physicians at referral centers  (n = 51) | | |
| --- | --- | --- | --- | --- | --- | --- |
|  | Initial survey | Follow-up survey | *P** | Initial survey | Follow-up survey | *P** |
| **Management** |  |  |  |  |  |  |
| Briefly mention that the patient has MASLD and do not proceed with any additional measures | 95 (16.7%) | 101 (17.7%) | 0.653 | 12 (23.5%) | 4 (7.8%) | 0.043 |
| Refer patient to the gastroenterology or a tertiary hospital | 174 (30.5%) | 180 (31.6%) | 0.668 | 14 (27.5%) | 18 (35.3%) | 0.423 |
| Take no additional measure if patient has normal AST and ALT levels. | 125 (21.9%) | 107 (18.8%) | 0.151 | 13 (25.5%) | 14 (27.5%) | 1.000 |
| Mention that the patient has fatty liver and recommend lifestyle modification | 340 (59.6%) | 340 (59.6%) | 1.000 | 31 (60.8%) | 35 (68.6%) | 0.423 |
| Order additional tests (blood glucose test, lipid panel, etc.) for metabolic syndrome (diabetes, dyslipidemia, etc.) | 271 (47.5%) | 284 (49.8%) | 0.370 | 26 (51.0%) | 29 (56.9%) | 0.663 |
| Order additional tests regarding liver fibrosis | 42 (7.4%) | 70 (12.3%) | 0.002 | 6 (11.8%) | 15 (29.4%) | 0.039 |
| Order additional tests to determine whether patient also has CVD | 20 (3.5%) | 45 (7.9%) | <0.001 | 2 (3.9%) | 3 (5.9%) | 1.000 |
| **Education** |  |  |  |  |  |  |
| Provide no further explanation | 30 (5.8%) | 48 (9.2%) | 0.026 | 4 (7.8%) | 6 (11.8%) | 0.683 |
| Explain the increased risk of disease progression into cirrhosis or liver cancer | 338 (65.1%) | 349 (67.2%) | 0.428 | 34 (66.7%) | 40 (78.4%) | 0.264 |
| Explain the increased risk of metabolic diseases (diabetes, dyslipidemia, etc.) | 404 (77.8%) | 395 (76.1%) | 0.481 | 43 (84.3%) | 40 (78.4%) | 0.606 |
| Explain the increased risk of CVD | 111 (21.4%) | 116 (22..4%) | 0.716 | 7 (13.7%) | 15 (29.4%) | 0.080 |
| Explain the increased risk of ischemic stroke | 70 (13.5%) | 82 (15.8%) | 0.252 | 5 (9.8%) | 9 (17.6%) | 0.289 |
| Explain the increased risk of extrahepatic cancer | 68 (13.1%) | 63 (12.1%) | 0.661 | 4 (7.8%) | 7 (13.7%) | 0.505 |

* *P* value was derived from McNemar test.

Abbreviations: MASLD, metabolic dysfunction-associated steatotic liver disease; PCP, primary care physician; CVD, cardiovascular disease.

**Supplementary Table 3.** Changes in the education of patients with MASLD among PCPs and physicians at referral centers before and after receiving educational materials on MASLD based on positive responses to ‘my primary area of practice' as a barrier to the management of MASLD

|  | PCPs  (n = 310) | | | Physicians at referral centers  (n = 28) | | |
| --- | --- | --- | --- | --- | --- | --- |
|  | Initial survey | Follow-up survey | *P** | Initial survey | Follow-up survey | *P** |
| **Management** |  |  |  |  |  |  |
| Briefly mention that the patient has MASLD and do not proceed with any additional measures | 71 (21.0%) | 75 (22.2%) | 0.749 | 9 (32.1%) | 3 (10.7%) | 0.114 |
| Refer patient to the gastroenterology or a tertiary hospital | 153 (45.3%) | 149 (44.1%) | 0.769 | 12 (42.9%) | 13 (46.4%) | 1.000 |
| Take no additional measure if patient has normal AST and ALT levels. | 74 (21.99%) | 75 (22.2%) | 1.000 | 7 (25.0%) | 11 (39.3%) | 0.221 |
| Mention that the patient has fatty liver and recommend lifestyle modification | 155 (45.9%) | 169 (50.0%) | 0.235 | 14 (50.0%) | 17 (60.7%) | 0.505 |
| Order additional tests (blood glucose test, lipid panel, etc.) for metabolic syndrome (diabetes, dyslipidemia, etc.) | 112 (33.1%) | 142 (42.0%) | 0.006 | 13 (46.4%) | 14 (50.0%) | 1.000 |
| Order additional tests regarding liver fibrosis | 21 (6.2%) | 40 (11.8%) | 0.007 | 3 (10.7%) | 8 (28.6%) | 0.182 |
| Order additional tests to determine whether patient also has CVD | 7 (2.1%) | 26 (7.7%) | <0.001 | 1 (3.6%) | 1 (3.6%) | 1.000 |
| **Education** |  |  |  |  |  |  |
| Provide no further explanation | 29 (9.4%) | 38 (12.3%) | 0.253 | 1 (3.6%) | 1 (3.6%) | 1.000 |
| Explain the increased risk of disease progression into cirrhosis or liver cancer | 181 (58.4%) | 198 (63.9%) | 0.129 | 20 (71.4%) | 22 (78.6%) | 0.724 |
| Explain the increased risk of metabolic diseases (diabetes, dyslipidemia, etc.) | 222 (71.6%) | 215 (69.4%) | 0.538 | 22 (78.6%) | 22 (78.6%) | 1.000 |
| Explain the increased risk of CVD | 51 (16.5%) | 73 (23.5%) | 0.017 | 5 (17.9%) | 11 (39.3%) | 0.114 |
| Explain the increased risk of ischemic stroke | 30 (9.7%) | 53 (17.1%) | 0.002 | 3 (10.7%) | 7 (25.0%) | 0.221 |
| Explain the increased risk of extrahepatic cancer | 26 (8.4%) | 31 (10.0%) | 0.522 | 2 (7.1%) | 5 (17.9%) | 0.450 |

* *P* value was derived from McNemar test.

Abbreviations: MASLD, metabolic dysfunction-associated steatotic liver disease; PCP, primary care physician; CVD, cardiovascular disease.

**Supplementary Table 4.** Changes in the education of patients with MASLD among PCPs and physicians at referral centers before and after receiving educational materials on MASLD based on positive responses to ‘patients’ low compliance’ as a barrier to the management of MASLD

|  | PCPs  (n = 535) | | | Physicians at referral centers  (n = 55) | | |
| --- | --- | --- | --- | --- | --- | --- |
|  | Initial survey | Follow-up survey | *P** | Initial survey | Follow-up survey | *P** |
| **Management** |  |  |  |  |  |  |
| Briefly mention that the patient has MASLD and do not proceed with any additional measures | 86 (16.1%) | 105 (19.6%) | 0.085 | 11 (20.0%) | 5 (9.1%) | 0.149 |
| Refer patient to the gastroenterology or a tertiary hospital | 141 (26.4%) | 151 (28.2%) | 0.407 | 17 (30.9%) | 21 (38.2%) | 0.423 |
| Take no additional measure if patient has normal AST and ALT levels. | 114 (21.3%) | 94 (17.6%) | 0.088 | 15 (27.3%) | 14 (25.5%) | 1.000 |
| Mention that the patient has fatty liver and recommend lifestyle modification | 330 (61.7%) | 340 (63.6%) | 0.490 | 33 (60.0%) | 36 (65.5%) | 0.606 |
| Order additional tests (blood glucose test, lipid panel, etc.) for metabolic syndrome (diabetes, dyslipidemia, etc.) | 269 (50.3%) | 280 (52.3%) | 0.442 | 29 (52.7%) | 30 (54.5%) | 1.000 |
| Order additional tests regarding liver fibrosis | 40 (7.5%) | 59 (11.0%) | 0.028 | 6 (10.9%) | 16 (29.1%) | 0.034 |
| Order additional tests to determine whether patient also has CVD | 20 (3.7%) | 42 (7.9%) | 0.002 | 2 (3.6%) | 4 (7.3%) | 0.683 |
| **Education** |  |  |  |  |  |  |
| Provide no further explanation | 29 (5.4%) | 38 (7.1%) | 0.281 | 2 (3.6%) | 6 (10.9%) | 0.221 |
| Explain the increased risk of disease progression into cirrhosis or liver cancer | 352 (65.8%) | 387 (72.3%) | 0.008 | 39 (70.9%) | 42 (76.4%) | 0.606 |
| Explain the increased risk of metabolic diseases (diabetes, dyslipidemia, etc.) | 422 (78.9%) | 418 (78.1%) | 0.793 | 48 (87.3%) | 43 (78.2%) | 0.302 |
| Explain the increased risk of CVD | 114 (21.3%) | 109 (20.4%) | 0.712 | 11 (20.0%) | 19 (34.5%) | 0.080 |
| Explain the increased risk of ischemic stroke | 75 (14.0%) | 91 (17.0%) | 0.130 | 6 (10.9%) | 13 (23.6%) | 0.023 |
| Explain the increased risk of extrahepatic cancer | 63 (11.8%) | 68 (12.7%) | 0.664 | 5 (9.1%) | 9 (16.4%) | 0.343 |

* *P* value was derived from McNemar test.

Abbreviations: MASLD, metabolic dysfunction-associated steatotic liver disease; PCP, primary care physician; CVD, cardiovascular disease.

**Supplementary Table 5.** Changes in the education of patients with MASLD among PCPs and physicians at referral centers before and after receiving educational materials on MASLD based on positive responses to ‘lack of appropriate medication’ as a barrier to the management of MASLD

|  | PCPs  (n = 492) | | | Physicians at referral centers  (n = 52) | | |
| --- | --- | --- | --- | --- | --- | --- |
|  | Initial survey | Follow-up survey | *P** | Initial survey | Follow-up survey | *P** |
| **Management** |  |  |  |  |  |  |
| Briefly mention that the patient has MASLD and do not proceed with any additional measures | 78 (15.9%) | 83 (16.9%) | 0.688 | 12 (23.1%) | 4 (7.7%) | 0.027 |
| Refer patient to the gastroenterology or a tertiary hospital | 124 (25.2%) | 133 (27.0%) | 0.439 | 14 (26.9%) | 20 (38.5%) | 0.181 |
| Take no additional measure if patient has normal AST and ALT levels. | 98 (19.9%) | 84 (17.1%) | 0.207 | 14 (26.9%) | 14 (26.9%) | 1.000 |
| Mention that the patient has fatty liver and recommend lifestyle modification | 309 (62.8%) | 306 (62.2%) | 0.869 | 33 (63.5%) | 34 (65.4%) | 1.000 |
| Order additional tests (blood glucose test, lipid panel, etc.) for metabolic syndrome (diabetes, dyslipidemia, etc.) | 247 (50.2%) | 267 (54.3%) | 0.118 | 28 (53.8%) | 29 (55.8%) | 1.000 |
| Order additional tests regarding liver fibrosis | 36 (7.3%) | 61 (12.4%) | 0.002 | 8 (15.4%) | 16 (30.8%) | 0.099 |
| Order additional tests to determine whether patient also has CVD | 17 (3.5%) | 41 (8.3%) | <0.001 | 2 (3.8%) | 2 (3.8%) | 1.000 |
| **Education** |  |  |  |  |  |  |
| Provide no further explanation | 24 (4.9%) | 39 (7.9%) | 0.045 | 3 (5.8%) | 6 (11.5%) | 0.371 |
| Explain the increased risk of disease progression into cirrhosis or liver cancer | 329 (66.9%) | 358 (72.8%) | 0.022 | 36 (69.2%) | 42 (80.8%) | 0.239 |
| Explain the increased risk of metabolic diseases (diabetes, dyslipidemia, etc.) | 391 (79.5%) | 377 (76.6%) | 0.223 | 45 (86.5%) | 40 (76.9%) | 0.302 |
| Explain the increased risk of CVD | 96 (19.5%) | 104 (21.1%) | 0.497 | 12 (23.1%) | 16 (30.8%) | 0.453 |
| Explain the increased risk of ischemic stroke | 59 (12.0%) | 72 (14.6%) | 0.177 | 7 (13.5%) | 10 (19.2%) | 0.450 |
| Explain the increased risk of extrahepatic cancer | 54 (11.0%) | 65 (13.2%) | 0.248 | 5 (9.6%) | 6 (11.5%) | 1.000 |

* *P* value was derived from McNemar test.

Abbreviations: MASLD, metabolic dysfunction-associated steatotic liver disease; PCP, primary care physician; CVD, cardiovascular disease.

**Supplementary Table 6.** Changes in the education of patients with MASLD among PCPs and physicians at referral centers before and after receiving educational materials on MASLD based on positive responses to ‘absence of a fee for this service’ as a barrier to the management of MASLD

|  | PCPs  (n = 576) | | | Physicians at referral centers  (n = 49) | | |
| --- | --- | --- | --- | --- | --- | --- |
|  | Initial survey | Follow-up survey | *P** | Initial survey | Follow-up survey | *P** |
| **Management** |  |  |  |  |  |  |
| Briefly mention that the patient has MASLD and do not proceed with any additional measures | 92 (16.0%) | 109 (18.9%) | 0.143 | 10 (20.4%) | 2 (4.1%) | 0.013 |
| Refer patient to the gastroenterology or a tertiary hospital | 160 (27.8%) | 170 (29.5%) | 0.433 | 13 (26.5%) | 20 (40.8%) | 0.096 |
| Take no additional measure if patient has normal AST and ALT levels. | 115 (20.0%) | 101 (17.5%) | 0.265 | 12 (24.5%) | 12 (24.5%) | 1.000 |
| Mention that the patient has fatty liver and recommend lifestyle modification | 356 (61.8%) | 348 (60.4%) | 0.610 | 32 (65.3%) | 35 (71.4%) | 0.579 |
| Order additional tests (blood glucose test, lipid panel, etc.) for metabolic syndrome (diabetes, dyslipidemia, etc.) | 292 (50.7%) | 299 (51.9%) | 0.657 | 26 (53.1%) | 30 (61.2%) | 0.522 |
| Order additional tests regarding liver fibrosis | 44 (7.6%) | 72 (12.5%) | 0.002 | 8 (16.3%) | 17 (34.7%) | 0.066 |
| Order additional tests to determine whether patient also has CVD | 21 (3.6%) | 50 (8.7%) | <0.001 | 2 (4.1%) | 3 (6.1%) | 1.000 |
| **Education** |  |  |  |  |  |  |
| Provide no further explanation | 32 (5.6%) | 44 (7.6%) | 0.156 | 1 (2.0%) | 3 (6.1%) | 0.480 |
| Explain the increased risk of disease progression into cirrhosis or liver cancer | 383 (66.5%) | 406 (70.5%) | 0.098 | 34 (69.4%) | 38 (77.6%) | 0.453 |
| Explain the increased risk of metabolic diseases (diabetes, dyslipidemia, etc.) | 456 (79.2%) | 449 (78.0%) | 0.608 | 42 (85.7%) | 41 (83.7%) | 1.000 |
| Explain the increased risk of CVD | 121 (21.0%) | 132 (22.9%) | 0.379 | 12 (24.5%) | 16 (32.7%) | 0.453 |
| Explain the increased risk of ischemic stroke | 78 (13.5%) | 95 (16.5%) | 0.108 | 7 (14.3%) | 9 (18.4%) | 0.683 |
| Explain the increased risk of extrahepatic cancer | 69 (12.0%) | 77 (13.4%) | 0.470 | 5 (10.2%) | 7 (14.3%) | 0.724 |

* *P* value was derived from McNemar test.

Abbreviations: MASLD, metabolic dysfunction-associated steatotic liver disease; PCP, primary care physician; CVD, cardiovascular disease.
